# Comprehensive Chemoproteomics Unveils Selective HMG-CoA Synthase 1 Inhibitors for Targeting Mevalonate Metabolism in Cancer

**DOI:** 10.1021/jacs.6c02556

**Published:** 2026-05-13

**Authors:** Liang Sun, Sang Ah Yi, Brittany Q. Pham, Antoine Mocellin, Sagnik Sen, M. Jason de la Cruz, Alban Ordureau, Heeseon An

**Affiliations:** † Chemical Biology Program, Sloan Kettering Institute, 5803 Memorial Sloan Kettering Cancer Center, New York, New York 10065, United States; ‡ School of Pharmacy, Sungkyunkwan University, Suwon 16419, Republic of Korea; § Department of Biopharmaceutical Convergence, Sungkyunkwan University, Suwon 16419, Republic of Korea; ∥ Department of Pharmacology, Weill Cornell Graduate School of Medical Sciences, New York, New York 10065, United States; ⊥ Cell Biology Program, Sloan Kettering Institute, Memorial Sloan Kettering Cancer Center, New York, New York 10065, United States; # Structural Biology Core, Memorial Sloan Kettering Cancer Center, New York, New York 10065, United States; ∇ Structural Biology Program, Sloan Kettering Institute, Memorial Sloan Kettering Cancer Center, New York, New York 10065, United States; ○ Tri-Institutional PhD Program, Memorial Sloan Kettering Cancer Center, New York, New York 10065, United States

## Abstract

Comprehensive target validation remains a significant bottleneck in chemical probe development, particularly for covalent inhibitors, where off-target reactivity can lead to toxicity. Using HMG-CoA synthase 1 (HMGCS1), an underexplored gatekeeper enzyme in the mevalonate pathway, we demonstrate how integrating orthogonal chemoproteomic methods can provide unbiased, comprehensive insights into the on- and off-target profiles of covalent inhibitors. Our study specifically highlights the limitations of traditional enrichment proteomics in distinguishing high-occupancy binders from low-occupancy binders, and it proposes a solution through a complementary scavenging proteomics approach that analyzes de-enriched fractions, providing target engagement ratios across the proteome. This framework facilitated the development of CNP7, a cyanopyrrolidine that covalently modifies HMGCS1′s catalytic cysteine with remarkable selectivity, as assessed by comprehensive chemoproteomics. A 2.29 Å cryo-EM structure reveals how CNP7 engages the catalytic cysteine within HMGCS1′s hydrophobic pocket. CNP7 treatment decreases HMG-CoA levels and induces global protein deprenylation within 4 h. Notably, CNP7 exhibits cell line-specific anticancer activity patterns that differ from those of statins, suggesting possible pathway node-specific vulnerabilities. Together, our study offers valuable chemical tools to modulate HMGCS1 activity and presents a framework for the rigorous characterization of covalent inhibitors in chemical biology and drug development.

## Graphical abstract

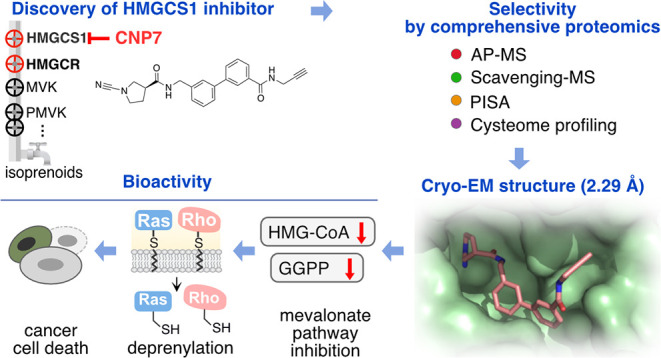

## Introduction

Chemical probes are essential for advancing our understanding of biological pathways and identifying potential therapeutic targets. However, comprehensive target validation remains a significant challenge, especially for covalent probes where off-target reactivity can cause unexpected toxicity and complicate the interpretation of biological outcomes. Several chemoproteomics techniques, such as covalent labeling-based affinity enrichment proteomics, thermal proteome profiling, cysteome profiling, and others, have been developed to map small-molecule targets across various stages of probe development, each with its own advantages and limitations.^1^ For example, affinity-based methods that follow ligand labeling can identify low-abundance targets through enrichment. However, many of these approaches do not provide target engagement rates or show how the small molecule influences the overall proteome. Cysteome profiling is a valuable tool for identifying electrophilic ligands that target specific cysteines in a cell-context-dependent manner, but the limited coverage of cysteine-containing peptides restricts its use to validating off-target reactivity.^2^ Thermal proteome profiling approach relies on changes in protein stability when a ligand binds, which does not require covalent attachment of the ligands to their targets.^3^ Nevertheless, the full extent of false-positive and false-negative rates for this approach remains unknown. Overall, more comprehensive comparisons of different chemoproteomics methods are needed to assess how effectively they identify targets of small-molecule probes. This would significantly assist in choosing the optimal approach at each stage of drug development. In this study, we systematically compare various proteomics methods as we develop a first-in-class covalent inhibitor for HMG-CoA synthase 1 (HMGCS1), a key enzyme in the mevalonate pathway, to evaluate each approach for target ID. Our findings show that integrating scavenging proteomics with affinity-based methods, along with repurposing cysteine profiling to monitor cysteine post-translational modifications, can overcome the limitations of traditional chemoproteomic approaches, thereby providing an unbiased functional readout for small-molecule probes targeting the mevalonate pathway.

Mevalonate pathway (MVP) enzymes convert acetyl-CoA into isopentenyl pyrophosphate, which is an essential precursor for the *de novo* synthesis of cholesterol and for key isoprenoids involved in post-translation modification of proteins.^4,5^ Ensuring that an adequate amount of mevalonate is produced at a given time is crucial for various aspects of cell functions, as excess production of mevalonate and sterol products contributes to the onset of human diseases such as cardiovascular disease and cancer.^6–8^ Accordingly, several enzymes within this pathway have emerged as therapeutic targets for cancer in the past decades.^8–15^ Although they show promise, existing noncovalent inhibitors of MVP enzymes have not yet achieved notable clinical anticancer success, primarily because of their low in vivo efficacy.^16–19^ Therefore, developing small molecules that act through distinct pharmacological mechanisms or targeting rate-limiting enzymes in specific cancer types may lead to more effective anticancer treatments by disrupting mevalonate metabolism.

HMGCS1, the first enzyme in the mevalonate pathway, is a largely overlooked pharmacological target. HMGCS1 has unique features that distinguish it from other MVP enzymes. A key characteristic of HMGCS1 is a catalytic cysteine in the active site that can be leveraged to develop covalent inhibitors and chemical probes, which may provide greater potency and consistency than noncovalent inhibitors. Moreover, our lab showed that HMGCS1 acts as a key control point in mevalonate flux for cell growth by coupling the mechanistic target of rapamycin complex 1 (mTORC1) activity to the mevalonate pathway.^20^ These unique biochemical traits suggest that targeting HMGCS1 with small molecules merits further investigation, particularly for its antiproliferative role in mTORC1-hyperactive cancer cells. Currently, there are few chemical tools available to modulate HMGCS1 activity. Hymeglusin,^21–23^ a natural product inhibitor of HMGCS1, has poor serum stability, leading to significant loss of effectiveness and limiting its use in cellular and in vivo studies.^24^ Ligustilide, another natural product from a plant reported to target HMGCS1 after its epoxidation in the liver, is highly unstable, and the increasing literature linking it to various cellular processes suggests that its reactivity is not specific to HMGCS1.^25,26^ Cysteome profiling studies with various electrophilic small molecules reported so far have failed to identify small molecules or fragments that react with HMGCS1′s catalytic cysteine.^27,28^ Therefore, new chemical approaches are needed to develop an HMGCS1 inhibitor and investigate this important metabolic enzyme.

Here, we introduce CNP7, a first-in-class HMGCS1 inhibitor that binds the catalytic cysteine of HMGCS1 via its cyanopyrrolidine electrophile. Dual activity-based profiling was used to develop CNP7 as a potent and selective HMGCS1 inhibitor candidate, followed by extensive chemoproteomic analyses, including affinity proteomics, thermal proteome profiling assays, and cysteome profiling, to evaluate CNP7′s reactivity in cells. The binding of CNP7 to HMGCS1 was further characterized using a 2.29 Å resolution cryo-EM structure, which rationalizes the observed affinity and binding mode. Cells treated with CNP7 show reduced HMG-CoA, increased levels of unprenylated proteins, and impaired proliferation, which can be rescued by adding geranylgeraniol, a downstream metabolite of HMG-CoA. Overall, this study provides a selective chemical tool for modulating HMGCS1 activity and offers insights into using unbiased chemoproteomics to assess the reactivity of covalent probes and their downstream effects.

## Results & Discussion

### CNP7 is a Potent First-in-Class Inhibitor of HMGCS1

Currently, no inhibitors for HMGCS1 with valid selectivity and prolonged potency are available in the field.^24^ We therefore set out to develop small-molecule inhibitors of HMGCS1. Because HMGCS1 contains a catalytic cysteine in its active site, we first consulted publicly available cysteome profiling data sets that report the engagement of reactive cysteines with various small molecule libraries.^27,28^ However, no such profiling studies detected the HMGCS1 Cys129 (C129) region because the tryptic peptide containing C129 is 46 amino acids in length (Figure S1A). Instead, analysis of the affinity chemoproteomic data sets of cyanopyrrolidine probes, mostly designed to target deubiquitinating enzymes, identified HMGCS1 as an off-target hit.^29–31^ Motivated by these unbiased yet consistent results from independent academic laboratories, we tested labeling of HMGCS1 by the simplest cyanopyrrolidine probe, GK16S, reported by the Gersch lab.^29^ We employed our activity-based probe profiling assay for HMGCS1 using Hymeglusin-Fluorescein (HG-FL), which specifically reacts with HMGCS1 C129 and forms a covalent bond (Figure S1B).^24^ The resulting HMGCS1 ∼ HG-FL adduct displays a fluorescence signal in the gel, thereby allowing us to measure the available catalytic cysteine levels of HMGCS1 in vitro and in cells after small molecule treatment. Incubation of recombinant HMGCS1 with GK16S for 1 h diminished the subsequent labeling of catalytic cysteine by HG-FL, indicating that the catalytic cysteine of HMGCS1 is occupied by GK16S, albeit at low efficiency (Figure S1C). Consistently, a cell-based assay demonstrated that GK16S covalently engages and copurifies with WT HMGCS1, but not with C129A mutant HMGCS1 expressed in HEK293T cells, confirming its specific reactivity toward Cysteine 129 (Figure S1D).

We next developed cyanopyrrolidine derivatives to improve the efficiency of labeling HMGCS1. HMGCS1 has a hydrophobic binding pocket that can accommodate two different substrates, acetyl-CoA and acetoacetyl-CoA (Figure S1E). This observation suggested that adding a large hydrophobic group to the 4-pentyn-1-amine of GK16S may enhance overall binding. We thus synthesized CNP molecules with varying levels of hydrophobicity and compared their binding efficacy to recombinant HMGCS1 using HG-FL activity-based probe *in vitro* (Figure 1A–C). Incorporating a benzyl group (CNP1 and CNP2) improved the labeling efficiency of the catalytic cysteine compared to GK16S (Figure 1B,C). Incorporating a methyl group at the benzylic position with the S configuration nearly abolished the binding, whereas the R configuration maintained the HMGCS1 labeling efficiency (CNP3 vs CNP4). Replacing the phenyl group in CNP1 with a naphthyl moiety (CNP5 and CNP6) did not enhance labeling, while replacing the phenyl group with a biphenyl group (CNP7 and CNP8) resulted in a significant increase in the engagement of the catalytic cysteine. CNP7 displayed the highest labeling efficiency in this *in vitro* assay, which corresponded with the in-cell labeling efficiency assessed by incubating HEK293T cells with the CNP compounds, followed by lysis and incubation with HG-FL (Figure 1D,E). The results of the HG-FL assay indicate that a concentration of 0.5 μM CNP7 charges over 70% of the catalytic cysteine residue of HMGCS1 in HEK293T cells within a 4-h time frame (Figure 1F).

**Figure 1:**
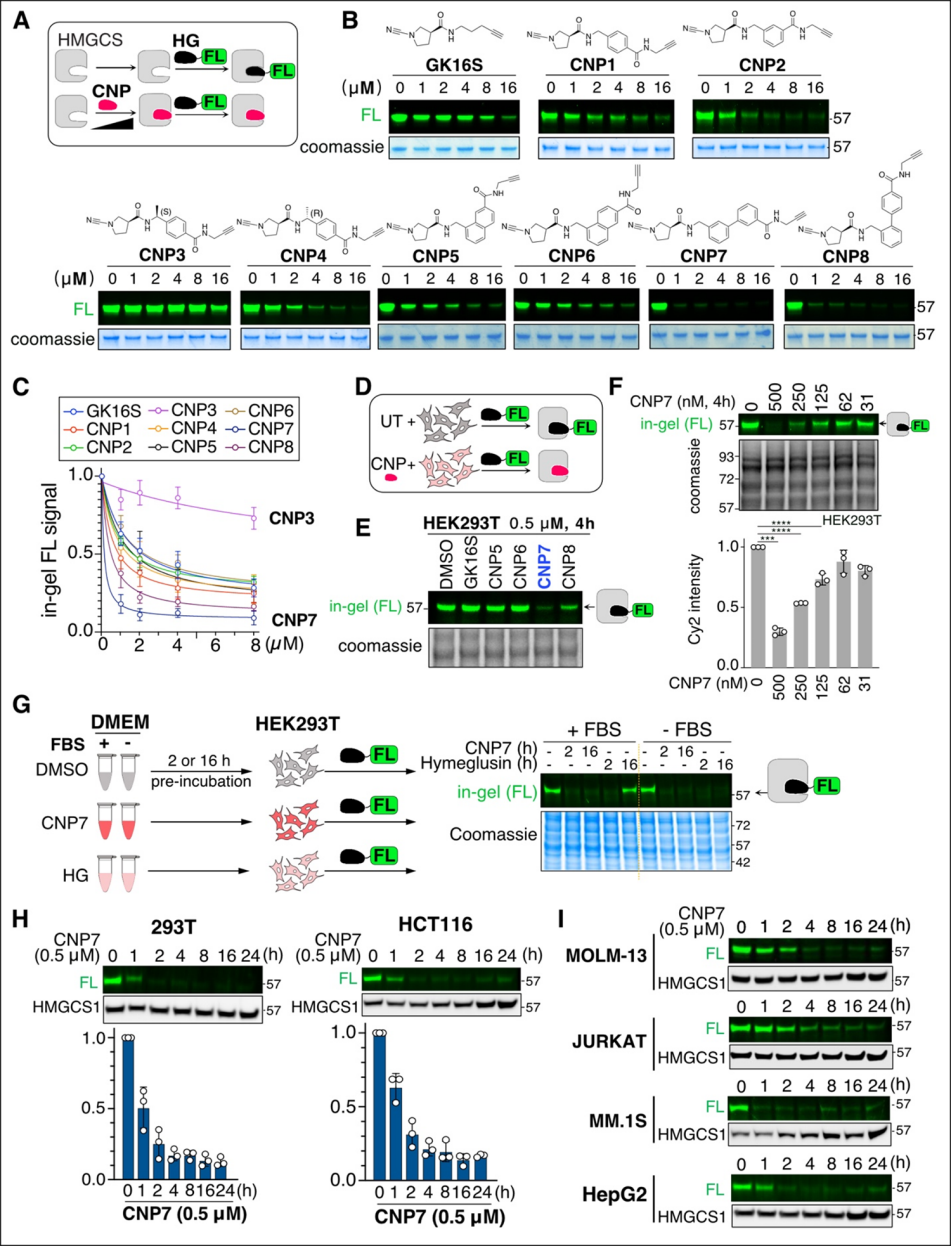
Assessment of cyanopyrrolidines binding to the catalytic cysteine of HMGCS1. (A) A workflow for labeling recombinant HMGCS1 protein with Hymeglusin-fluorescein (HG-FL) activity-based probe after treatment with cyanopyrrolidine derivatives (CNP, 1h). (B) In-gel fluorescence assay using HG-FL probe showed a dose-dependent labeling of recombinant HMGCS1 (1 μM, 1h) by CNP derivatives. (C) Quantification graph of the in-gel fluorescence assay results in panel B. Data is represented as means ± s.d. (*n* = 3 or 5 biological replicates). (D) Scheme of the HG-FL assay for HMGCS1 labeling by CNP in cells. (E) Activity-profiling after treating HEK293T cells with the indicated chemicals shows that CNP7 exhibits the highest efficacy at occupying the catalytic cysteine of HMGCS1. (F) HEK293T cells were treated with increasing concentrations of CNP7 for 4 h, followed by cell lysis, HG-FL treatment, and in-gel fluorescence analysis. The quantification graph is shown at the bottom (means ± s.d. of biological triplicates). (G) DMEM media supplemented with or without 10% FBS were preincubated with 0.5 μM of CNP7 or HG for 2 or 16 h at 37 °C. Then, HEK293T cells were incubated with the media for 2 h, followed by lysis and in vitro reaction with the HG-FL probe. (H) HEK293T and HCT116 cells were treated with CNP7 (0.5 μM) for the indicated time points, followed by lysis and reaction with the HG-FL probe. Subsequently, in-gel fluorescence analysis and immunoblotting with HMGCS1 antibody were performed. Quantification of relative fluorescence intensity from biological triplicates is presented at the bottom (means ± s.d.). (I) The in-gel fluorescence assay using HG-FL, combined with immunoblotting with an anti-HMGCS1 antibody, shows the HMGCS1 catalytic cysteine occupancy results for MOLM-13, Jurkat, MM.1S, and HepG2 cells treated with 0.5 μM CNP7 at different time points.

We previously reported that Hymeglusin has inherently poor serum stability, limiting its usage for pharmacologic studies in tissue culture or animal studies.^24^ We directly compared the serum stability of Hymeglusin and CNP7 by preincubating them in DMEM with or without fetal bovine serum (FBS) before adding them to HEK293T cells. Subsequent activity profiling showed that CNP7 potently reacted with HMGCS1 even after 16 h of preincubation in a serum-containing medium, whereas Hymeglusin completely lost its reactivity toward HMGCS1 (Figure 1G). Consistently, CNP7′s labeling of HMGCS1 persisted after 24 h of incubation in various cell lines (Figure 1H,I).

### Dual Activity-Profiling Assays Evaluate the Potency and Selectivity of CNP Derivatives

Each synthesized CNP molecule contains an alkyne group, which offers a functional handle for click chemistry-based activity profiling in the proteome. After treating cells with CNP7, we divided the cell lysates into two fractions: one was treated with HG-FL to assess intracellular HMGCS1 occupancy. The other underwent click chemistry with an azide probe conjugated to tetramethyl rhodamine (TMR) or Fluorescein (FL) for selectivity assessment (Figure 2A). CNP7 occupied the catalytic cysteine of HMGCS1 within 60 min, and a concomitant increase in TMR signal at three distinct molecular weights was observed (Figure 2B). The primary TMR signal observed near 57 kDa coincided with the band reactive to the anti-HMGCS1 antibody (Figure 2C). To improve the selectivity of CNP7, we further derivatized CNP7 in the region close to the cyanopyrrolidine warhead group (Figure 2D). Subsequent analyses showed that CNP9 or CNP11 did not compromise the potency, whereas CNP9′s diastereoisomer (CNP10) or adding a large substituent to the pyrrolidine ring (CNP12 and CNP13) dramatically reduced the HMGCS1 labeling (Figure 2E). Dual activity profiling showed that CNP11 treatment further increased band intensity around 25 kDa, while CNP9 decreased it compared to CNP7 (Figure 2F). However, CNP9 demonstrated slightly reduced potency in HMGCS1 engagement compared to CNP7 (Figure 2G,H).

**Figure 2:**
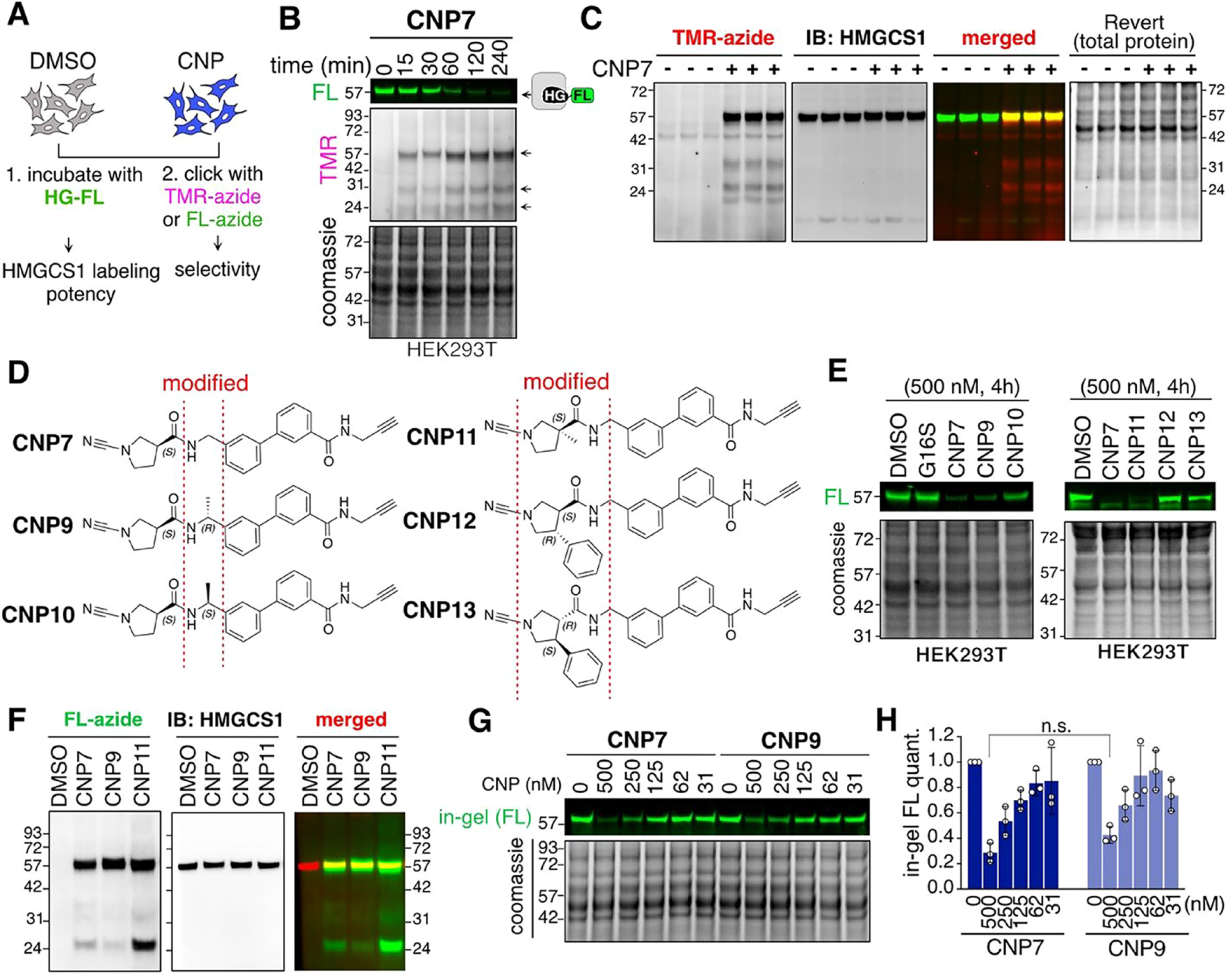
Dual activity-based profiling of CNP derivatives to assess their reactivity and selectivity in cells. (A) A workflow of orthogonal in-gel fluorescence analyses. Cells treated with vehicle (DMSO) or CNP molecules were lysed and divided into two fractions; one was incubated with HG-FL to assess HMGCS1 labeling potency, and the other portion was subjected to copper-catalyzed [3 + 2] cycloaddition reaction (click chemistry) with tetramethyl rhodamine (TMR) or fluorescein (FL)-azide to test their reactivity toward the proteome. (B) Orthogonal in-gel fluorescence assay of 293T cells treated with CNP7 (0.5 μM) for the indicated time points. Arrows: potential CNP7 binders. (C) 293T cells treated with CNP7 (0.5 μM) for 1 h were lysed and subjected to click reaction with TMR-azide, followed by in-gel fluorescence assay and immunoblotting with HMGCS1 antibody. Revert: total protein stain. (D) Chemical structures of CNP7 derivatives. (E) The HG-FL-based in-gel fluorescence assay of 293T cells treated with the indicated compounds (0.5 μM) for 4 h. (F) 293T cells were treated with the indicated compounds (0.5 μM) for 4 h, followed by click chemistry with FL-azide for global reactivity profiling, and immunoblotting with the HMGCS1 antibody. (G) The HG-FL-based activity profiling of 293T cells treated with the indicated concentrations of CNP7 or CNP9 for 4 h. (H) Quantification of relative fluorescence intensity from biological triplicates of panel G (means ± s.d.).

### Complementary Affinity-Proteomics Reveal Exceptional Selectivity of CNP7 and CNP9 for HMGCS1

To identify covalent interactors of CNP7 and CNP9, we first compared the CNP7- and CNP9-enriched proteomes by conjugating them to biotin via click chemistry, followed by streptavidin enrichment and tandem mass tag (TMT)-based quantification (Figure S2A).^29,32^ Proteomics analysis quantified the relative enrichment of 835 proteins (Figure S2B and Table S1). Twelve and 10 proteins were enriched by CNP7 and CNP9, respectively, with statistical significance, of which HMGCS1 showed the strongest enrichment in both cases. Other enriched proteins included a subfamily member of aldehyde dehydrogenases and Cathepsin Z, a cysteine protease. Several proteins, including aldehyde dehydrogenases, DESI1, NIT1, and PARK7, which coprecipitated with CNP7 and CNP9, were previously identified as interactors of cyanopyrrolidine-containing molecules through click-chemistry-based affinity purification mass spectrometry.^29,31,33^ However, this conventional affinity purification mass-spectrometry (AP-MS) data lacks information on the overall percentage of engagement for each protein by CNP probes, and the enrichment result can be affected by protein abundance in HEK293T cells. Additionally, we observed that the in-gel fluorescent signal intensity of some CNP-protein adducts varied depending on the click reaction conditions, including the choice of reducing agents and the dyes conjugated to azide (Figure S2C,D). We also detected ISOC1 enrichment in the streptavidin bead eluate, even when cells were treated with DMSO (Figure S2E,F), which explains the leftward shift of ISOC1 in Figure S2B. This indicates a previously overlooked background effect of click reagents, in addition to earlier reports of the inherent background of click chemistry caused by the formation of thiotriazole protein conjugates when adding alkyne-bearing probes.^34^ Overall, our findings suggest that employing click chemistry in chemoproteomics can lead to artifacts at multiple stages, independent of the cyanopyrrolidine probes’ reactivity.

To overcome these limitations, we synthesized biotin-functionalized CNP7 and CNP9 probes and performed competitive AP-MS analysis that bypasses the click reaction (Figure 3A and Tables S2 and S3). HEK293T cells were pretreated with DMSO, CNP7, or CNP9, then lysed and incubated with the corresponding CNP-biotin probes. Of 482 quantified proteins, six and five proteins showed a significant enrichment in DMSO pretreated cells compared to CNP7 or CNP9 pretreated cells, respectively (Figure 3B). Again, HMGCS1 was detected as one of the strongly enriched proteins in both cases. Notably, some of the earlier hits from direct AP-MS, such as aldehyde dehydrogenase 9 family member A1 (ALDH9A1) and deSUMOylating isopeptidase (DESI1), showed little change in this competitive AP-MS analysis, suggesting that only a small portion of these proteins interact with the CNP probes. We next validated all the hits identified in our competitive AP-MS analysis by immunoblotting (Figure 3C,D). Importantly, comparing the input, elute, and flow-through fractions side by side provided additional information. First, most HMGCS1 in the lysate was pulled down by CNP-biotin in DMSO-pretreated cells, whereas in CNP-pretreated cells, little enrichment was observed. ISOC1 exhibited a similar pattern to HMGCS1, but with less enrichment in the eluate, confirming our competitive AP-MS results for both proteins. Second, BTD, ALDH1A2, ALDH1B1, and ERP44 unexpectedly showed little enrichment in the eluate regardless of the CNP-pretreatment, indicating they are low-occupancy binders with only a small portion of their cellular pools engaged by CNP7/9-biotin probes. Since competitive AP-MS analysis compares only enriched proteins in the eluate using TMT-based mass spectrometry, the resulting ratios may be artificially inflated.

**Figure 3:**
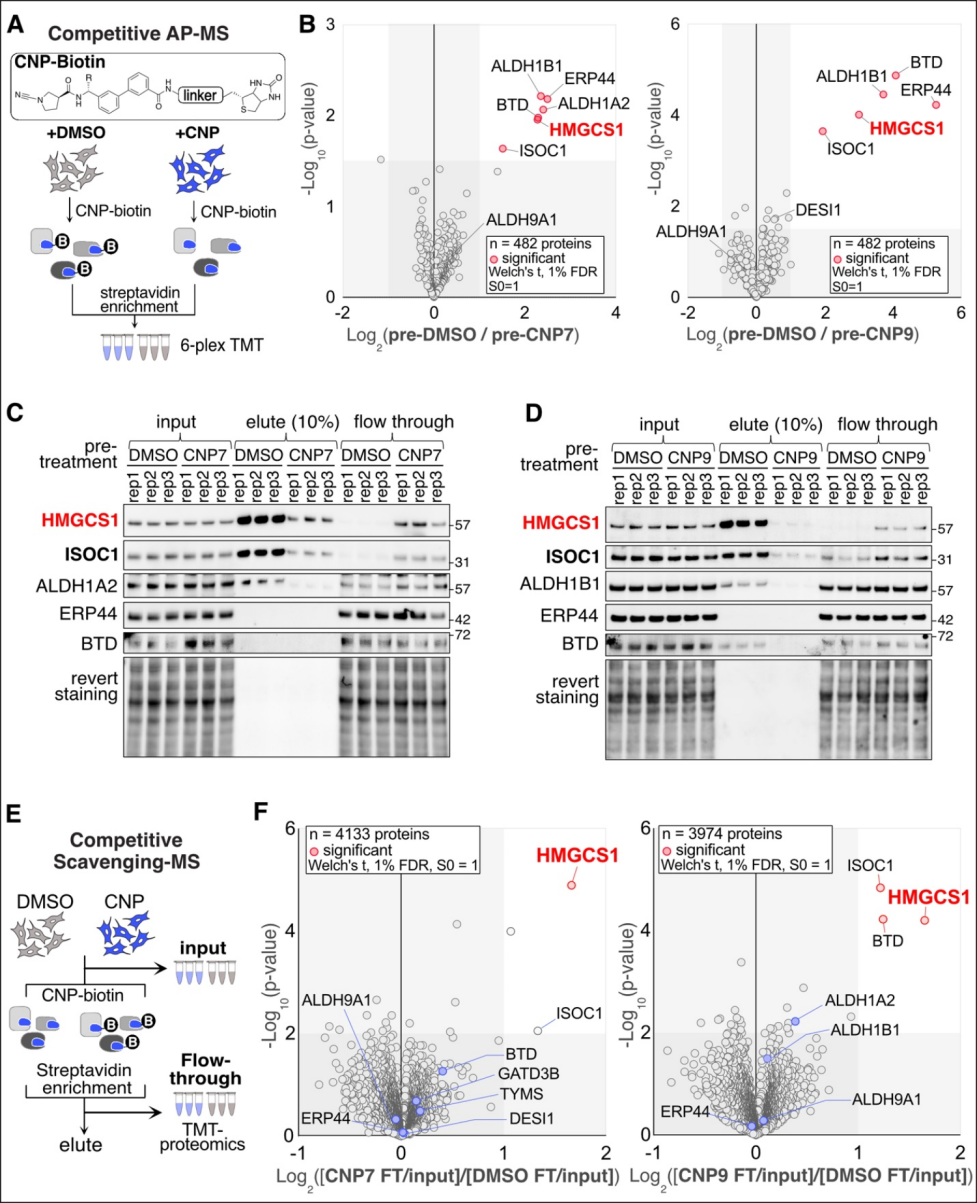
Determining the proteome-wide reactivity of CNP7 and CNP9 (A) A workflow of competitive AP-MS. Lysates of 293T cells pretreated with CNP7 or CNP9 (0.5 μM) for 1 h were incubated with CNP7- or CNP9-biotin (5 μM) for 1 h. After streptavidin bead enrichment, the eluates were analyzed using TMT- proteomics. R: H (CNP7) or CH_3_ (CNP9), Linker: triazole-ethylamine-ethoxy-ethylamine (B) Volcano plots of the – log_10_ (p-value) versus the log_2_ (pre-DMSO/pre-CNP7) or (pre-DMSO/pre-CNP9) treated cells. Two-sided Welch’s *t* test (adjusted to 1% FDR for multiple comparisons, S0 = 1). (C, D) 293T cells treated as described in panel A were probed with the indicated antibodies. Rep: replicate. (E) A workflow of competitive scavenging MS. (F) Volcano plots of the – log_10_(*p*-value) versus the log_2_-transformed ratio of CNP7/DMSO (left) or CNP9/DMSO (right). TMT signal of flow-through (FT) of each protein was normalized to that of the input. *n* = 3 or 4 biological replicates. p-values were calculated by two-sided Welch’s *t* test (adjusted to 1% FDR for multiple comparisons, S0 = 1).

Based on this unexpected discovery, we reasoned that comparing the input and flow-through fractions of the competitive affinity purification steps, which we term competitive scavenging mass spectrometry analysis, could help eliminate low-occupancy hits from AP-MS data and provide additional, complementary insights (Figure 3E and Tables S4 and S5). Indeed, CNP7-biotin scavenging proteomics data demonstrated a strong depletion of HMGCS1 (Figure 3F, left). CNP9-biotin also significantly depleted HMGCS1, followed by ISOC1 and BTD (Figure 3F, right). These proteomic findings were consistent with the immunoblotting results, and other hits identified in both direct and competitive AP-MS showed no significant depletion. Taken together, scavenging proteomic analysis reveals that both CNP7 and CNP9 strongly target HMGCS1, whereas other proteins like BTD and ISOC1 are labeled to a lesser extent under the same conditions.

### Thermal Proteome Profiling of CNP7 and CNP9 Confirms Their Selectivity for HMGCS1

Affinity-based enrichment methods are effective for detecting strong small-molecule interactors, such as those mediated by covalent interactions, but they may overlook weak or noncovalent interactors. To thoroughly evaluate the interactors of CNP7 and CNP9, we used the proteome integral solubility alteration (PISA) analysis, which detects changes in thermal stability caused by small molecule treatment across a wide range of temperatures (Figure 4A,B and Tables S6 and S7).^3,35,36^ The soluble fractions from both HEK293T and HCT116 lysates, treated or left untreated with CNP (10 μM), were subjected to a heat gradient, and the pooled samples were centrifuged and analyzed using TMT-based proteomics (Figure 4B). Because PISA compares the area under the curve, it improves the ability to identify hits compared with using a single temperature. Mass-spectrometry analysis quantified 5218 soluble proteins in HEK293T and 6533 in HCT116 cells (Figures 4C–D and S3A–C). Among these, HMGCS1 was consistently stabilized in both cell lines treated with CNP7 and CNP9. Additionally, phosphodiesterase 6D (PDE6D) was consistently stabilized by CNP7 and CNP9 treatment, although it was not identified as a hit in our prior affinity-based proteomic analysis. Immunoblotting of the soluble fraction of HEK293T cells after a heat gradient showed significant stabilization of HMGCS1 and PDE6D at 5 μM, with HMGCS1 being particularly strongly stabilized (Figure 4E). Reducing the CNP7′s concentration to 0.5 μM consistently stabilized HMGCS1 but was less effective for PDE6D stabilization (Figure 4F). Since PDE6D has a hydrophobic pocket that binds to prenylated or farnesylated proteins, it is possible that CNP7 and CNP9 could bind to the hydrophobic pocket of PDE6D through a noncovalent interaction at high concentration.^37^ In summary, the unbiased PISA assay indicates that CNP7 and CNP9 mainly and strongly bind to HMGCS1. They may also interact noncovalently with other cellular proteins like PDE6D and RIOK2. However, direct binding assays are required to confirm these interactions.

**Figure 4:**
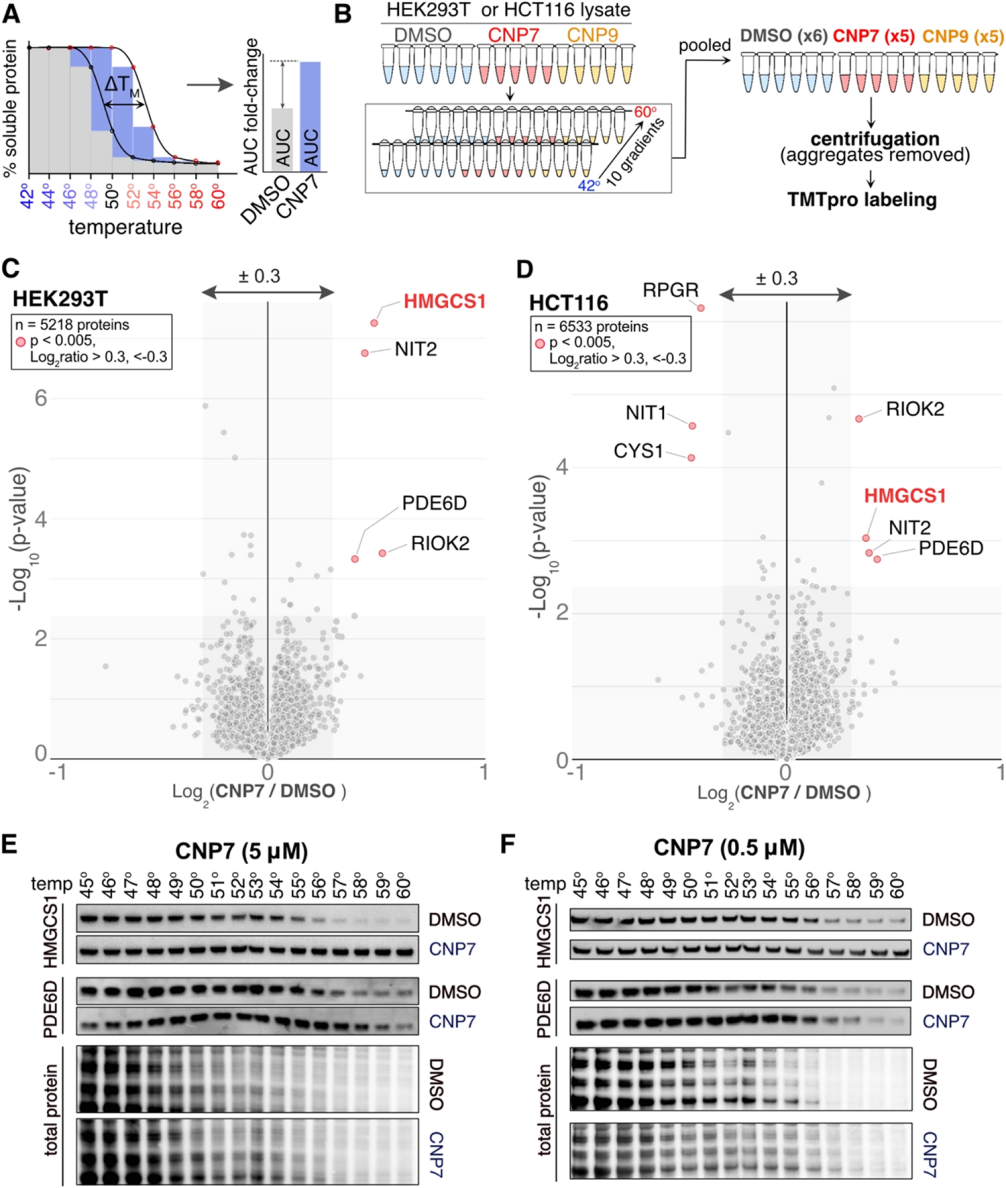
Proteome integral solubility alteration (PISA) assay using CNP7. (A) Schematic of the PISA assay. (B) HEK293T or HCT116 cell lysates were left untreated or treated with CNP7 (10 μM) or CNP9 (10 μM) for 15 min, followed by an application of a heat gradient. The samples were then pooled, centrifuged, digested, and labeled with TMTpro for analysis by mass spectrometry. (C, D) Volcano plots of the – log_10_-transformed *p*-value versus the log_2_-transformed ratio of CNP7/DMSO analyzed from HEK293T cells are shown in panel C, and those for HCT116 are in panel D. *n* = 6 biological replicates for the DMSO-treated condition and 5 for the CNP7-treated condition. p-values were calculated by two-sided Welch’s *t* test (adjusted to 1% FDR for multiple comparisons, S0 = 0.285). (E, F) Immunoblotting to verify the thermal stability changes of HMGCS1 and PDE6D after incubating the HEK293T lysates with CNP7. Total protein, assessed by revert staining, indicates loss of the soluble proteome due to application of the heat gradient.

### Structural Basis for CNP7′s Binding to HMGCS1

We sought to characterize how CNP7 binds to HMGCS1 to better understand its specificity. Recombinant human HMGCS1 protein was incubated with CNP7 to form an HMGCS1 ∼ CNP7 adduct, achieving 90% labeling as determined by the HG-FL assay, prior to cryo-EM grid preparation. Single-particle cryo-electron microscopy (cryo-EM) analysis revealed the complex structure at 2.29 Å resolution (Figures 5, S4, and Table S8). Two HMGCS1 monomers formed a homodimeric complex with C2 symmetry, as previously reported, and the CNP7 appeared in the hydrophobic pocket of HMGCS1 near Cys129 (Figure 5A,B). The cyanamide group of CNP7 reacted with the catalytic Cys129, as expected from our biochemical assays (Figure 5C). The backbone amide of Cys129 and the hydroxy group of Ser377 are in proximity for potential hydrogen bond formation to form an “oxy-anion hole”-like pocket for the imide nitrogen, which can stabilize intermediates during and after the reaction between C129 and the cyano group of CNP7. Histidine 264, part of the catalytic triad, ^95^Glu-^129^Cys-^264^His, forms a strong hydrogen bond with the oxygen of the carbamoyl group in CNP7. Hydrogen bonds that extend through the propargyl amide, Lys273, and Tyr267 at the entrance of the catalytic pocket further contribute to stabilizing the CNP7 in the HMGCS1 pocket (Figure 5D). This may explain why biphenyl group-containing CNP analogs, such as CNP7 and 8, showed better binding properties compared to phenyl or naphthyl analogs. Additionally, extended hydrophobic interactions between CNP7 and neighboring hydrophobic side chains were observed (Figure 5E,F). In summary, the cryo-EM structure of the HMGCS1 ∼ CNP7 adduct confirms that the catalytic cysteine forms a covalent bond with CNP7, and that hydrophilic and hydrophobic interactions throughout the binding pocket of HMGCS1 and CNP7 may help stabilize the CNP7 during and after the reaction with C129.

**Figure 5:**
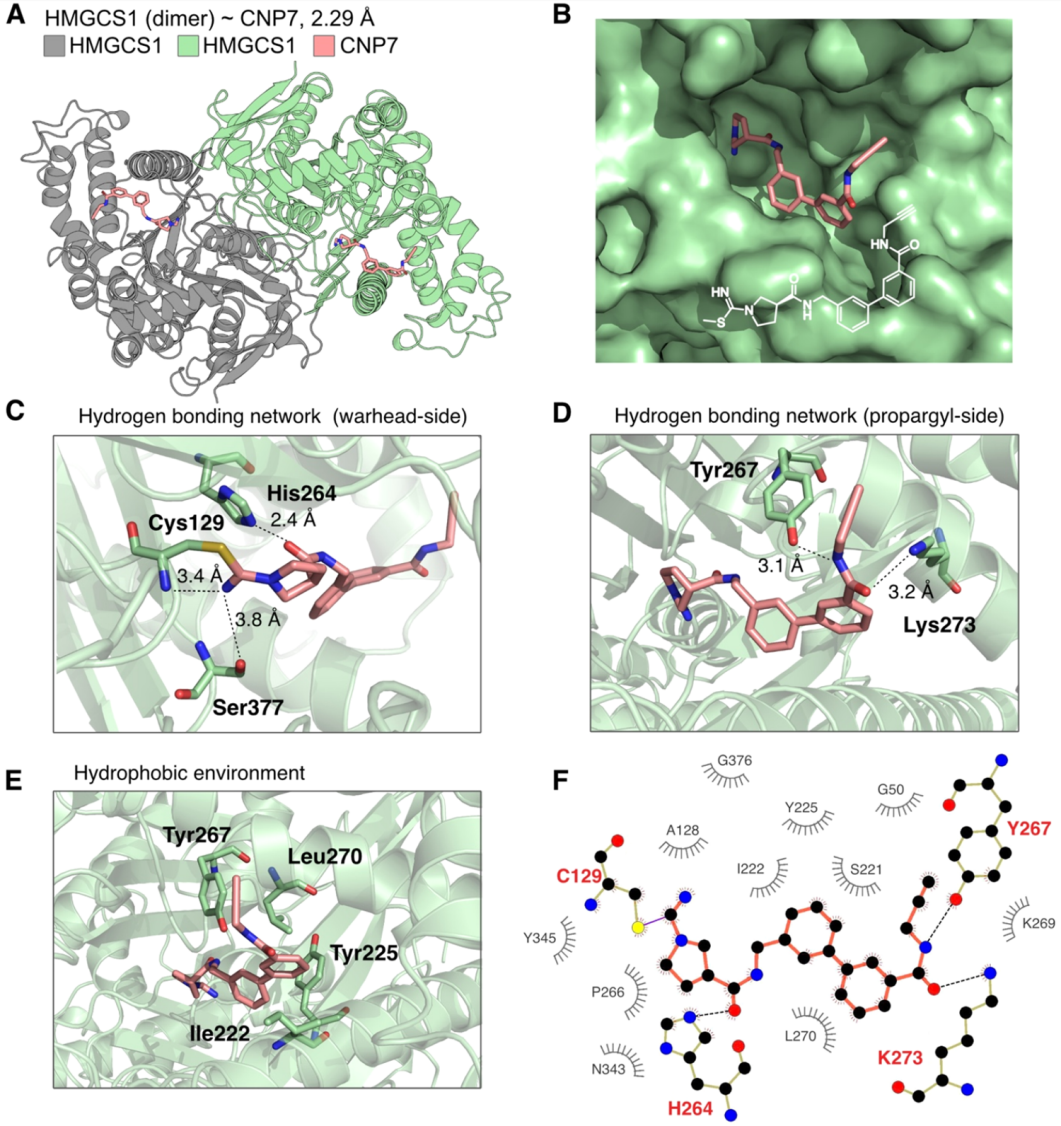
Cryo-EM structure of the HMGCS1 ∼ CNP7 covalent adduct. (A) Structure of HMGCS1 homodimer (gray and green) in complex with CNP7 (pink). (B) Surface model showing CNP7 fitting into the cavity of the HMGCS1 active site. The orientation of CNP7, which forms an iso-thiourea bond with HMGCS1 C129, is displayed in white. (C) Close-up view of the reactive warhead side of CNP7, highlighting the oxyanion hole (Cys129 and Ser377) bound by the iso-thiourea nitrogen. A strong hydrogen bond, represented by a 2.4 Å distance between His264 and the central carbamoyl oxygen, is also observed. Hydrogen bonds are shown as dotted lines, with their lengths indicated. (D) Close-up view of the propargyl amine side of CNP7 near the entrance of the HMGCS1 binding pocket, highlighting the hydrogen bonds between the propargyl amine and Lys273 and Tyr267. (E) Close-up view of the hydrophobic residues, I222, Y225, Y267, and L270, near the biphenyl group of CNP7. (F) 2D depiction of the ligand binding pocket observed in the HMGCS1 ∼ CNP7 structure, highlighting the hydrophobic interactions between HMGCS1 and the core of CNP7.

### CNP7 Reduces Cellular HMG-CoA Levels and Global Protein Prenylation

After confirming the specificity of CNP7 and CNP9, we conducted a series of systematic analyses to assess the pharmacologic effects of HMGCS1 inhibition in cells. Initially, we investigated the impact of CNP molecules on the level of cellular HMG-CoA, the direct product of HMGCS1, using an antibody that identifies proteins modified by a 3-hydroxy-3-methylglutaryl (HMG) moiety. Prior studies reported that inhibiting HMGCR with statins increases intracellular HMG-CoA levels, which leads to the formation of a covalent bond between the HMG moiety and nucleophilic side chains on fatty acid synthase (FASN) through a nonenzymatic reaction (Figure 6A, top).^38,39^ Notably, levels of HMGylated FASN were found to have a positive correlation with intracellular HMG-CoA levels. Indeed, addition of Simvastatin induced an HMGylated protein band in HCT116 cells (Figure 6B, first two lanes). Adding CNP7 resulted in a significant decrease, with 5 μM required to completely inhibit HMGylation over a period of 48 h in HCT116 cells (Figure 6B). This increased requirement for CNP7 was attributed to the substantial upregulation of HMGCS1, arising from the mevalonate pathway inhibition as a negative feedback mechanism. Consistently, the HG-FL labeling assay conducted on the same cell extract indicated substantial engagement of the catalytic cysteine of HMGCS1 by 5 μM CNP7, with a minor level of catalytically active HMGCS1 still detected.

**Figure 6:**
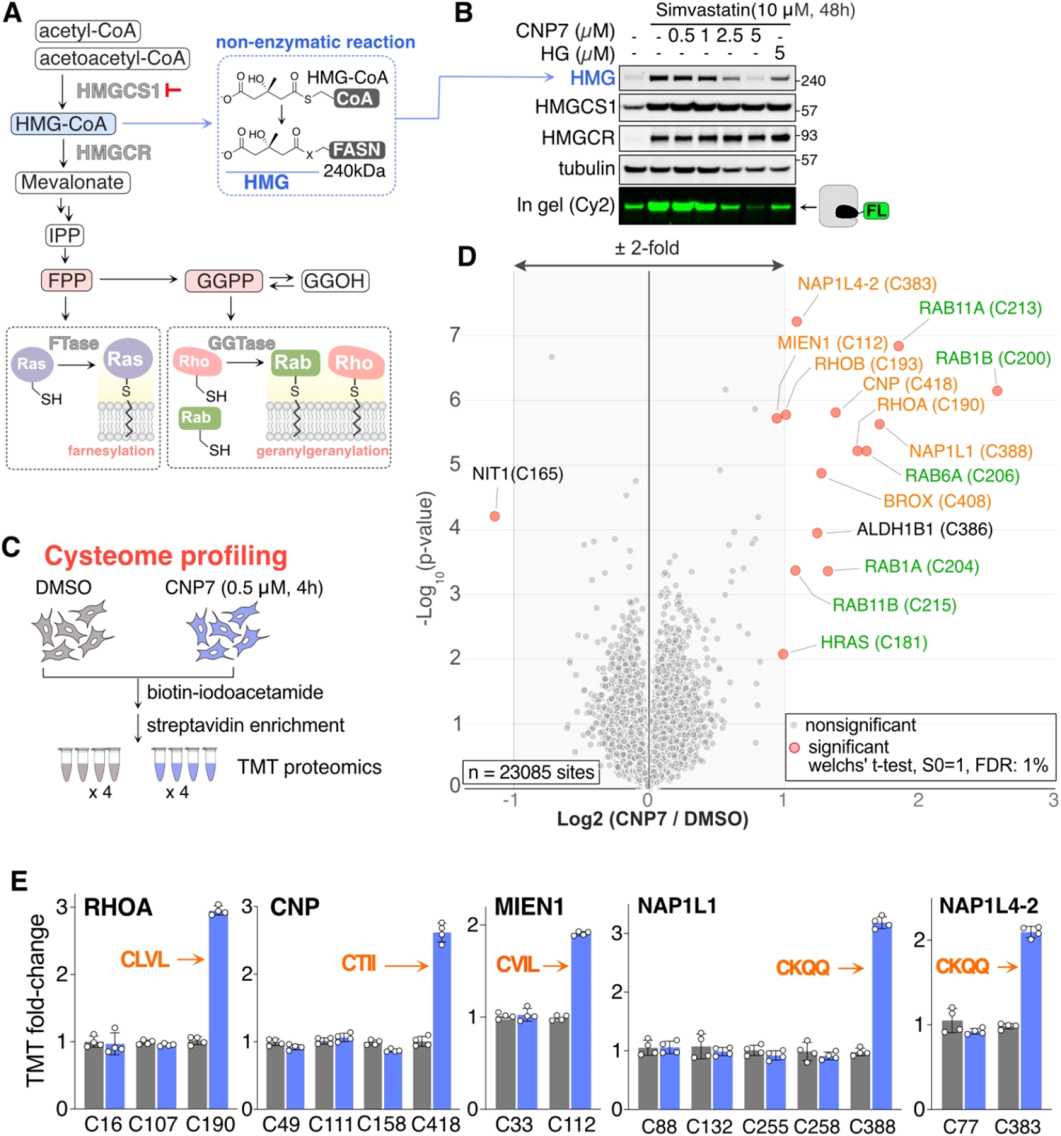
CNP7 treatment lowers HMG-CoA levels and reduces global protein prenylation. (A) A schematic illustrating the mevalonate pathway flux. The changes in levels of downstream metabolites can be assessed through immunoblotting of HMGylated FASN (for HMG-CoA level) or protein prenylation status (for FPP and GGPP production). (B) Lysates of HCT116 cells treated as indicated were incubated with HG-FL probe for 1 h, followed by an in-gel fluorescence assay. The proteins on the gel were then transferred to a PVDF membrane and probed with the indicated antibodies, including an anti-HMG antibody to detect HMGylated FASN. HG: Hymeglusin (C) A workflow of cysteome profiling as a method to detect the prenylation status of intracellular proteins. (D) Volcano plot of the – log_10_-transformed p-value versus the log_2_-transformed ratio of biotinylated peptides derived from CNP7/DMSO-treated cells, prepared as shown in panel C. *P*-values were calculated by two-sided Welch’s *t* test (adjusted to 1% FDR for multiple comparisons, S0 = 1). The statistically significant hits are circled in red (15 proteins). Of those, peptides containing a CAAX motif (geranylgeranylation site) are labeled in orange, and farnesylation sites are marked in green. A total of 23085 cysteine-containing peptides were quantified. *n* = 4 biological replicates. (E) Relative TMT signal intensities of tryptic peptides originating from the same protein indicate that cysteine sites containing the CAAX motif are significantly enriched in CNP7-treated cells, whereas the other sites show no difference. Red: sequence of the CAAX motif identified in the corresponding peptide.

We next investigated how HMGCS1 inhibition affects protein prenylation, a key destination of HMG-CoA metabolites (Figure 6A, bottom). Since protein farnesylation and geranylgeranylation mainly target cysteine side chains, we reasoned that cysteome profiling may detect CNP7-induced changes in the prenylation status of the global proteome (Figure 6C). HEK293T cells were treated with DMSO or 0.5 μM CNP7 for 4 h, followed by cell lysis and biotin-iodoacetamide treatment. Following enrichment, we used TMT-based proteomics and quantified 23,085 cysteine-containing peptides, with no missing values across eight samples (Figure 6D and Table S9). As previously mentioned, a tryptic peptide that includes HMGCS1 C129 is 46 amino acids long and was not identified in our data set. NIT1 C165 was the only quantified site that decreased notably after CNP7 treatment, by about 50%. Conversely, there was a notable rise in peptides from many proteins, mostly small GTPases, that are known to undergo geranylgeranylation (orange labels) or farnesylation (green labels). Among the geranylgeranylation substrates, five proteins were quantified with more than two peptides, but only those with C-terminal peptides containing the CAAX motif, a standard geranylgeranylation site, showed a significant increase in biotin-iodoacetamide labeling upon CNP7 treatment (Figure 6E). This data indicates that a 4-h CNP7 treatment led to the accumulation of unprenylated form of proteins, rather than an increase in their overall protein levels. In conclusion, cysteome profiling uniquely detected the downstream effect of CNP7: loss of prenylation caused by HMGCS1 inhibition. These data reinforce our findings that CNP7 can strongly inhibit HMGCS1, thereby suppressing protein prenylation.

### CNP7 Inhibits Cell Proliferation by Disrupting Geranylgeranyl Pyrophosphate Metabolism

Does CNP7 have an antiproliferative effect? HEK293T cells started showing cell death at 5 μM of CNP7 after 48 h of treatment, but Hymeglusin showed no such effect on cell viability (Figure 7A). Colony formation assays also revealed a significant reduction in both the number and size of HCT116 colonies in the presence of CNP7, but not Hymeglusin (Figure S5A). Supplementing the media with geranylgeraniol (GGOH), which is converted to geranylgeranyl pyrophosphate (GGPP) in cells, completely rescued the cell death caused by CNP7 (Figure 7B). Collectively, CNP7 inhibits HMGCS1, thereby blocking the synthesis of HMG-CoA and isoprenoids. This leads to the death of HEK293T and HCT116 cells over time, primarily due to a lack of cellular geranylgeranyl pyrophosphate, rather than to sterol depletion.^40–42^

**Figure 7:**
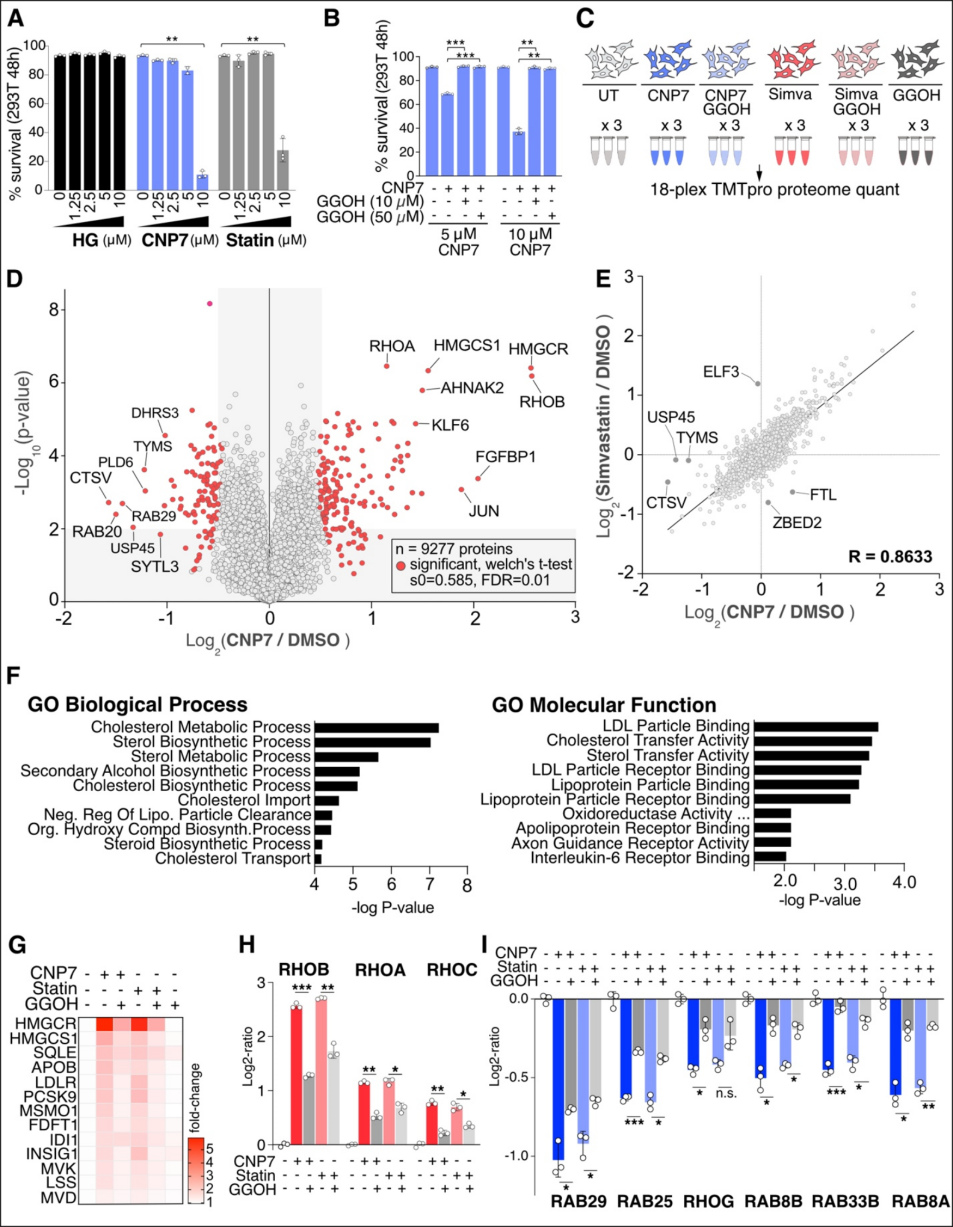
Global proteome alterations following CNP7 or Simvastatin treatment. (A) Cell viability assay of 293T cells after being treated with increasing concentrations of Hymeglusin (HG), CNP7, and Simvastatin (Statin) for 48 h. (B) Reduced viability of 293T cells by CNP7 was rescued by the supplementation of geranylgeraniol (GGOH). (C) A workflow of TMTpro-based global proteome analysis of HCT116 cells treated with CNP7 (5 μM), Simvastatin (10 μM), and/or GGOH (10 μM) for 24 h. (D) Volcano plot of – log10-transformed p-value versus the log2-transformed ratio of CNP7/untreated. *n* = 3 biological replicates. Two-sided Welch’s *t* test (adjusted to 1% FDR for multiple comparisons, S0 = 0.585). (E) Linear regression plot visualizing the relationship between proteome changes by CNP7 (*x*-axis) and Simvastatin (*y*-axis). (F) Gene ontology analyses of the statistically upregulated proteins (G) Fold-change of proteins in the mevalonate/sterol pathway are presented as a heat map. (H, I) Select proteins that increase after CNP7 treatment and are reversed by GGOH supplementation are shown in H, while those that decrease after CNP7 treatment and are reversed by GGOH are shown in I.

To better understand the effect of CNP7 on the global proteome, we analyzed the proteome response after 24 h of CNP7 treatment or combined CNP7 and GGOH treatment in HCT116 cells (Figure 7C). This approach will distinguish proteome changes caused by defects in the geranylgeranyl pathway from other changes. Cells treated with Simvastatin, Simvastatin with GGOH, and GGOH alone were also compared as controls. The subsequent multiplexed proteomic analysis quantified 9277 proteins, with 167 proteins statistically significantly upregulated and 108 downregulated (Figure 7D and Table S10). Importantly, the cells treated with CNP7 and Simvastatin exhibited highly correlated proteome changes, with an r-value of 0.8633 (Figure 7E). Consistently, gene ontology analysis of the significant hits showed enrichment in the sterol-related pathway terms, further supporting its functional specificity (Figure 7F). Several enzymes in the mevalonate pathway were upregulated upon either CNP7 or Simvastatin treatment through a feedback effect, which were reversed by GGOH cotreatment (Figure 7G).

Since GGOH supplementation reversed CNP7-induced cell death, we curated known geranylgeranylation targets, including Rho, Rab, Rac, and Rap proteins, and examined their proteome-level changes (Figure S5B). Among the 86 proteins quantified, RhoA, B, and C proteins showed particularly strong induction by CNP7, which was reversed by GGOH cotreatment (Figure 7H). Conversely, several Rab proteins showed downregulation upon CNP7 treatment, and only a small subset of them were reversed by GGOH treatment (Figure 7I). Other geranylgeranylation targets did not change in abundance. Therefore, it is speculative that these specific Rho and Rab proteins, whose expression levels are significantly changed by CNP7 and restored by GGOH treatment, might play a role in CNP7-mediated cell death. Together, analyses of global proteome changes upon CNP7 treatment support its primary effect on the mevalonate pathway via HMGCS1 inhibition and suggest a possible cause of cell death. We note that these cellular assays used 5 μM CNP7, a higher concentration than the 0.5 μM used for selectivity profiling. Although the strong correlation between CNP7 and Simvastatin-induced proteome changes and the full rescue by GGOH supplementation support on-target activity as the primary driver of cell death, we cannot fully exclude that off-target engagement at this higher concentration contributed to some of the observed effects.

### Susceptibility to CNP7 is Cell Line Dependent

Accumulating studies show that cell susceptibility to statins varies by cell type, with complex underlying mechanisms needing further molecular studies.^8,43^ We evaluated the antiproliferative effect of CNP7 across 18 cancer cell lines and compared it with Simvastatin and Hymeglusin to gain insights into the cellular response upon inhibition of HMGCS1 and HMGCR, the two initial enzymes in the mevalonate pathway (Figures 8A and S6). The area under the curve (AUC) measurement indicated that Hymeglusin showed no adverse effects, confirming its low efficacy in serum-containing media, as we previously reported.^24^ Some CNP7-resistant cell lines also showed resistance to Simvastatin, indicating that resistance to CNP7 is a broad trait against mevalonate pathway inhibition. Interestingly, some cell lines, such as RPE1 and MFE296, were sensitive to CNP7 but less to Simvastatin (Figure 8B). MM1.S and SNU449 cell lines showed similar sensitivity curves that saturated at earlier doses after treatment with CNP7 or Simvastatin. Taken together, our data reveal a clear pattern linking CNP7 with Simvastatin, as well as a unique response characterized by sensitivity solely to CNP7. Future studies involving genetic depletion of HMGCS1 in CNP7-sensitive cell lines will help distinguish on-target from any potential off-target contributions at these higher concentrations.

**Figure 8:**
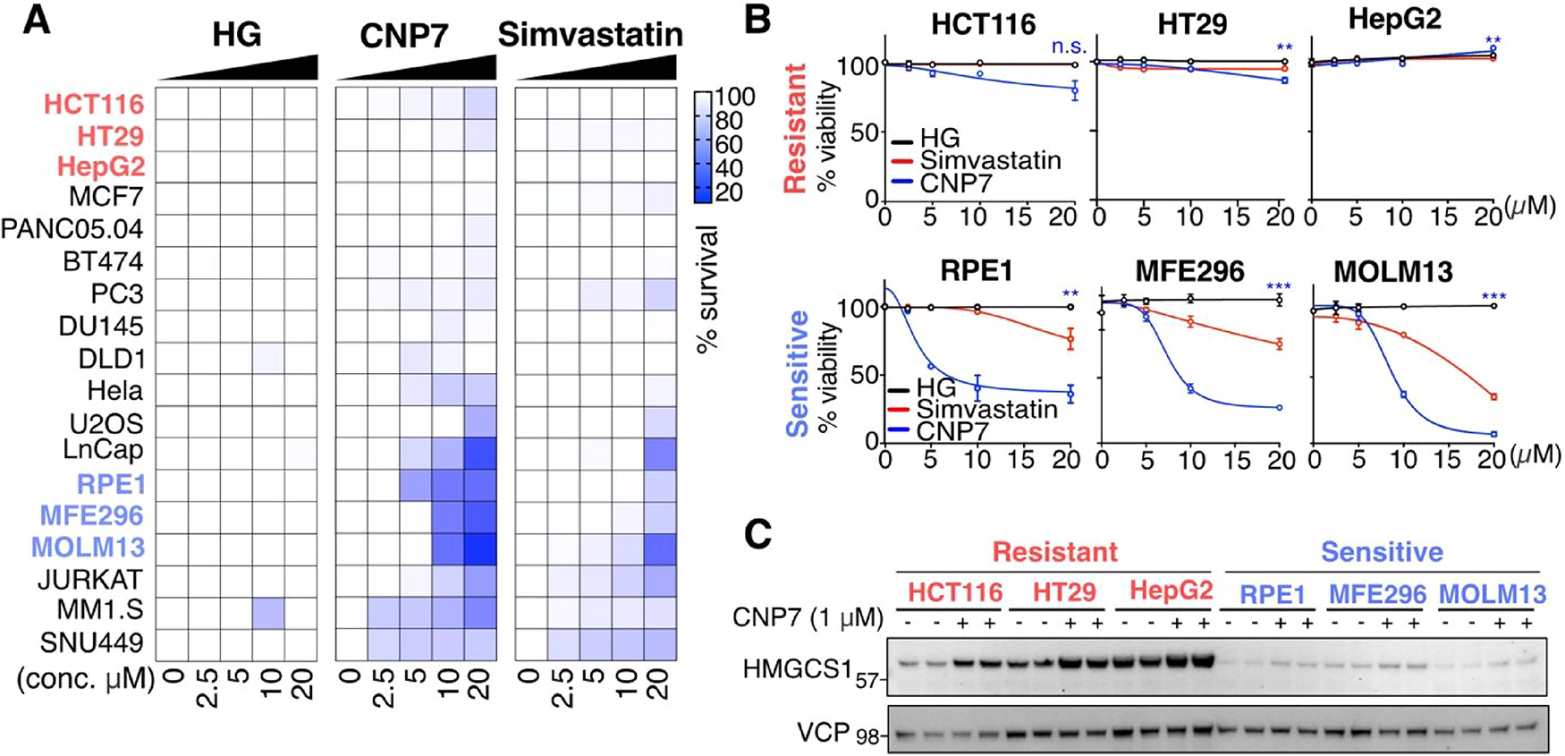
HMGCS1 inhibition by CNP7 exhibits cell line-specific response. (A) Heatmap presentation of the Area Under the Curve (AUC) for 18 cancer cell lines viability after 72 h of treatment with Hymeglusin (HG), CNP7, or Simvastatin. Cell lines resistant to both CNP7 and Simvastatin are highlighted in red, while those sensitive to CNP7 and resistant to Simvastatin are highlighted in blue. Means of *n* = 3 biological triplicates are shown. (B) Line graphs depicting three resistant (HCT116, HT29, and HepG2) and three sensitive cell lines (RPE1, MFE296, and MOLM13) from panel A. Means ± s.d. of biological triplicates. (C) Three resistant cell lines (HCT116, HT29, and HepG2) and three sensitive cell lines (RPE1, MFE296, and MOLM13) were evaluated for expression of HMGCS1 after 24 h of treatment with 1 μM CNP7.

To gain insight into the cause of CNP7-sensitivity and resistance, we measured the HMGCS1 levels across CNP7-sensitive or resistant cell lines using an immunoblotting approach (Figure 8C). Overall, the resistant cell lines compared in this study exhibited higher steady-state HMGCS1 levels, which were further elevated upon CNP7 treatment, as a feedback response. The sensitive cell lines exhibited lower baseline expression, and the increase in expression upon CNP7 treatment was less pronounced. Our combined data suggest a correlation between the overall expression levels of HMGCS1 before and after CNP7 treatment and their susceptibility. However, more cell lines need to be tested to strengthen this correlation.

## Conclusion

In this study, we employed comprehensive proteomic analyses to guide target identification and the on-target effects of the selective HMGCS1 inhibitor, CNP7. Combined information provides multiple layers of insight into the advantages and disadvantages of individual chemoproteomics approaches in evaluating covalent probes. First, our scavenging-MS methodology complements the comparative AP-MS technique, which provides broader coverage of proteome reactivity and helps in ruling out low-occupancy hits from AP-MS hits. PISA data using CNP probes highlights the importance of evaluating noncovalent interactors of reactive small molecules, as these may also engage with other proteins through noncovalent interactions, an often overlooked aspect in developing covalent inhibitors. Further validation is needed to determine if binding to potential off-target proteins triggers a functional response, since some off-targets might act as silent binders. Investigating alterations in the global proteome following prolonged probe treatment may facilitate this assessment. Lastly, native cysteome profiling with biotin iodoacetamide uniquely detected the secondary effect of CNP7, namely, the loss of protein post-translational modification by isoprenoids due to HMGCS1 inhibition. Of note, our cysteome profiling did not quantify CNP7 interactors identified by affinity-enrichment proteomics. Considering that 204,707 cysteine-containing tryptic peptides and over 45,000 ligandable cysteines are reported to exist in theory, the current iodoacetamide-based cysteome profiling approach may have limited coverage for their use in off-target evaluation.^2,44^ Overall, our systematic approaches resulted in the development of first-in-class inhibitors of HMGCS1, which bind to its hydrophobic pocket and react with the catalytic cysteine through a cyanamide moiety.

Cyanamides were first identified to target cysteine proteases, such as cathepsins and dipeptidyl peptidase IV, in the early 2000s.^45–47^ Over the past few years, multiple laboratories have focused on developing cyanopyrrolidines as targeted inhibitors for specific deubiquitinating enzymes (DUBs),^29–31,33,48–50^ building on the results initially reported by Mission Therapeutics.^51^ MTX652, a cyanopyrrolidine derivative that strongly inhibits USP30, is being evaluated in a Phase II clinical trial for acute kidney injury, underscoring the potential of cyanopyrrolidine-based small molecules as therapeutic agents. We found that HMGCS1 often appears as an off-target binder of the cyanopyrrolidine probes in prior studies. This finding prompted us to develop cyanopyrrolidine-based selective inhibitors of HMGCS1 and to thoroughly validate their on- and off-targets through a multilayered approach, including comprehensive chemoproteomics, chemical, structural, and cell biological methods. Using CNP7, we found that targeting HMGCS1 is a promising alternative to statins and exhibits distinct vulnerabilities in specific cell lines. Thus, HMGCS1 inhibitors not only contribute to the pharmacological arsenal for mechanistic studies but also demonstrate potential as anticancer therapeutics by perturbing the mevalonate pathway. Our findings emphasize that targeting individual proteins within the same metabolic pathway results in different cellular responses that influence treatment outcomes, highlighting the need to develop small-molecule tools for individual metabolic enzymes within the pathway. This observation may parallel the small-molecule inhibitors of Raf, Ras, and MEK, each of which produces distinct cellular outcomes.

While our data suggests that CNP7 and CNP9 are highly selective for HMGCS1, there are limitations. First, we found that CNP7 labels other targets, albeit to a lesser degree. Notably, multiple aldehyde dehydrogenase family members appeared enriched in the competitive MS and scavenging MS methods. Improving CNP7′s selectivity and potency could further minimize off-target effects. We think that our 2.29 Å cryo-EM structure of CNP7 bound to HMGCS1 can provide helpful insights for this optimization. Second, the cell line-specific sensitivity of CNP7, distinct from statins, is intriguing and may involve multiple factors, including potential off-target effects at the higher concentrations used in these assays. This calls for further follow-up studies in various cell lines beyond the 19 we tested in this study, and a mechanistic investigation. Third, the isothiourea adduct formed between cyanopyrrolidines and cysteine residues is partially reversible under nondenaturing conditions. We observed slow recovery of free catalytic cysteine in vitro (Figure S7A–C), and the reversibility of HMGCS1 ∼ CNP7 adducts can introduce artifacts during sample preparation for affinity-based proteomics (Figure S7D,E). The extent to which this reversibility affects sustained target engagement warrants further investigation. Despite these limitations, CNP7 will be a valuable tool for investigating the unique pharmacology of targeting HMGCS1, opening many possibilities for its use as a compound, whether as monotherapy or in combination with statins.

## Supplementary Material























## Data Availability

All data are available in the main text or the Supporting Information. The MS TMT proteomic data have been deposited in the MassIVE repository under the data set identifier MSV000099720. Cryo-EM structural study-related data is deposited to the RCSB Protein Data Bank (RCSB PDB), data set ID: D_1000302134 and PDB ID: 9ZAW.

## Notes

Supporting Information file contains supplementary Figures S1–S7, material and methods sections, the chemistry synthetic procedure, and ^1^H and ^13^C NMR spectra of biologically tested compounds (PDF)
Table S1. Direct affinity-proteomics after click chemistry (Figure S2B) (XLSX)
Table S10. Total proteome changes upon CNP7 or Simvastatin (related to Figure 7) (XLSX)
Table S2. Competitive affinity-proteomics using CNP7-biotin probe (related to Figure 3B, Left Panel) (XLSX)
Table S3. Competitive affinity-proteomics using CNP9-biotin probe (related to Figure 3B, Right Panel) (XLSX)
Table S4. Scavenging-proteomics using CNP7-biotin probe (related to Figure 3F, Left) (XLSX)
Table S5. Scavenging-proteomics using CNP9-biotin probe (related to Figure 3F, Right) (XLSX)
Table S6. PISA assay using CNP7 and CNP9 on HCT116 (related to Figures 4 and S3) (XLSX)
Table S7. PISA assay using CNP7 and CNP9 on HEK293T (related to Figures 4 and S3) (XLSX)
Table S8. Cryo-EM data collection, refinement, and validation statistics (PDF)
Table S9. Native cysteome profiling (related to Figure 6E) (XLSX)
