# Supplementary material for: Comprehensive Chemoproteomics Unveils Selective HMG-CoA Synthase 1 Inhibitors for Targeting Mevalonate Metabolism in Cancer
Source: J Am Chem Soc. 2026 May 13;148(20):20705–19. doi: 10.1021/jacs.6c02556 (PMC13220267; doi:10.1021/jacs.6c02556)
Supplement: Supplementary file 1 [file ja6c02556_si_001.pdf]

Comprehensive Chemoproteomics Unveils Selective HMG-CoA Synthase1 Inhibitors for Targeting Mevalonate Metabolism in Cancer

Liang Sun[1,‡], Sang Ah Yi[1,2,3‡], Brittany Q. Pham[1,4], Antoine Mocellin[5], Sagnik Sen[6], M. Jason de la Cruz[6,7], Alban Ordureau[5], and Heeseon An[1,4,8]*

Supporting Information

[1]Chemical Biology Program, Sloan Kettering Institute, Memorial Sloan Kettering Cancer Center, New York, NY, USA.
[2]School of Pharmacy, Sungkyunkwan University, Suwon, Republic of Korea.
[3]Department of Biopharmaceutical Convergence, Sungkyunkwan University, Suwon, Republic of Korea.
[4]Department of Pharmacology, Weill Cornell Graduate School of Medical Sciences, New York, NY, USA.
[5]Cell Biology Program, Sloan Kettering Institute, Memorial Sloan Kettering Cancer Center, New York, NY, USA.
[6]Structural Biology Core, Memorial Sloan Kettering Cancer Center, New York, NY, USA.
[7]Structural Biology Program, Sloan Kettering Institute, Memorial Sloan Kettering Cancer Center, New York, NY, USA.
[8]Tri-Institutional PhD Program, Memorial Sloan Kettering Cancer Center, New York, NY, USA.

[‡]These authors contributed equally to this work

**To whom correspondence should be addressed:* anh@mskcc.org

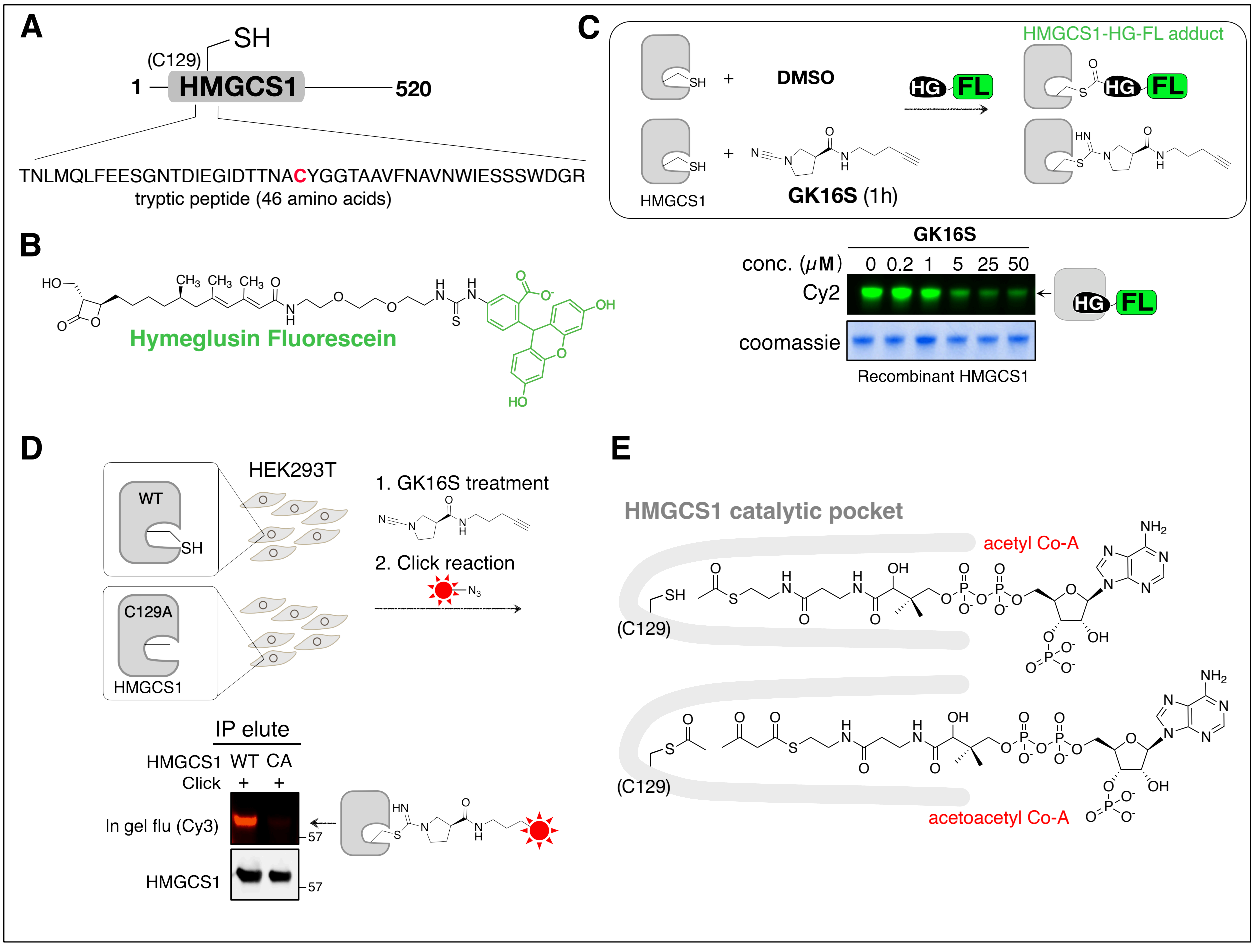

Fig S1. Labeling of the catalytic cysteine in HMGCS1 using GK16S. (A) Sequence of the predicted tryptic peptide that includes the catalytic cysteine of human HMGCS1. (B) Structure of Hymeglusin-Fluorescein (HG-FL) activity-probe. (C) Top: Workflow of HG-FL-based activity profiling using recombinant HMGCS1 (1 μM) and GK16S. Bottom: In-gel fluorescence analysis indicates a GK16S concentration-dependent decrease in fluorescent signaling, suggesting that GK16S labels the catalytic cysteine of HMGCS1. (D) HEK293T cells ectopically expressing WT or C129A mutant HMGCS1-V5 were incubated with GK16S (1 μM, 24h). Then, the cell lysates were subjected to click chemistry with TMR-azide, followed by enrichment of HMGCS1-V5 using anti-V5 beads and copper-mediated click chemistry with azide-TMR. The samples were analyzed by in-gel fluorescence for TMR signal, and then by immunoblotting with an anti-HMGCS1 antibody. (E) Schematics showing the two substrates of HMGCS1.

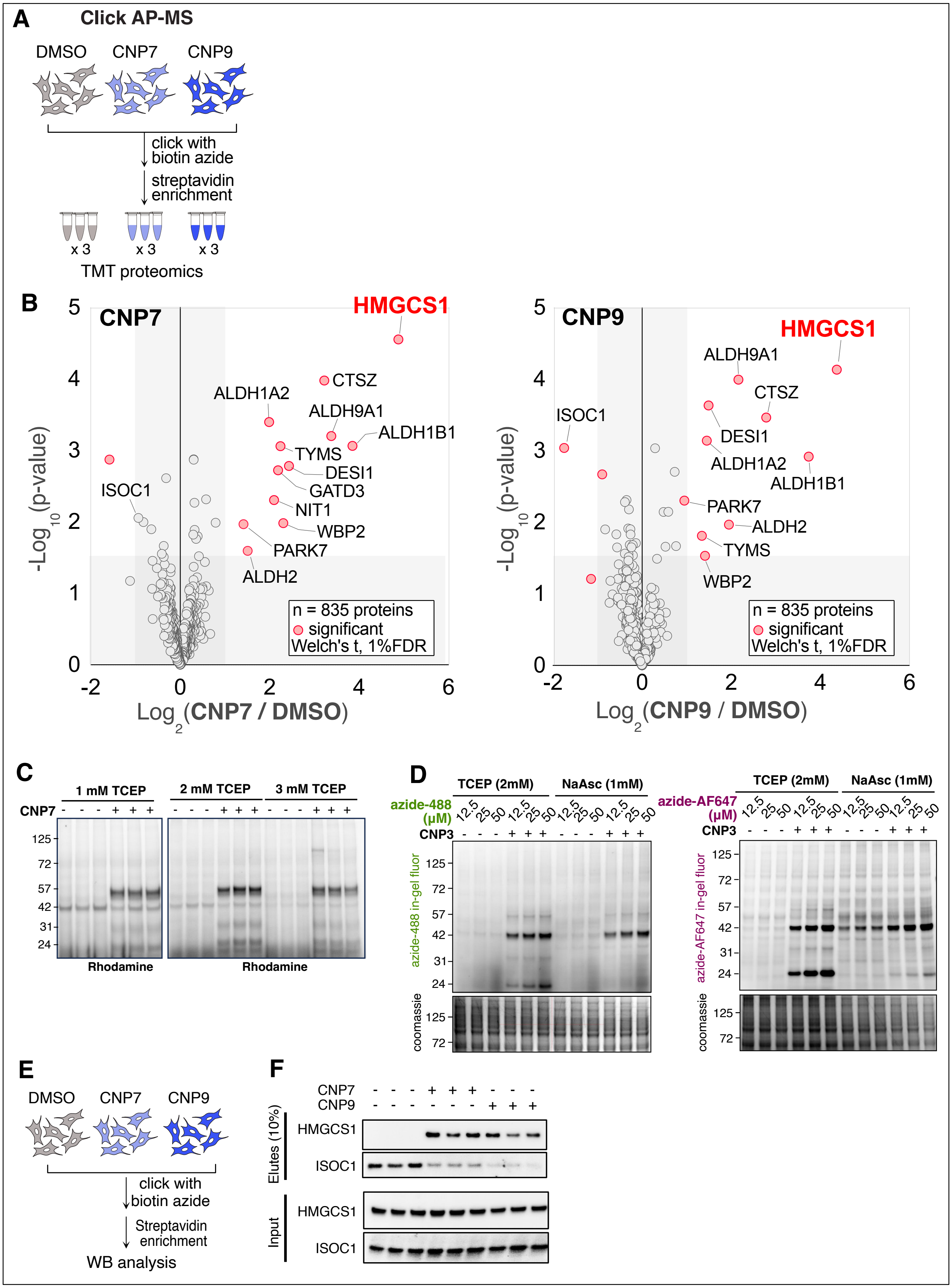
A
Click AP-MS
DMSO
CNP7
CNP9
click with biotin azide
streptavidin enrichment
x 3
TMT proteomics
B
CNP7
HMGCS1
CTSZ
ALDH1A2
ALDH9A1
ALDH1B1
TYMS
DESI1
GATD3
NIT1
WBP2
PARK7
ALDH2
ISOC1
n = 835 proteins
significant
Welch's t, 1%FDR
-Log10 (p-value)
Log2(CNP7 / DMSO)
CNP9
HMGCS1
ALDH9A1
CTSZ
ISOC1
DESI1
ALDH1A2
ALDH1B1
PARK7
ALDH2
TYMS
WBP2
Log2(CNP9 / DMSO)
C
1 mM TCEP
2 mM TCEP
3 mM TCEP
CNP7
Rhodamine
D
TCEP (2mM)
NaAsc (1mM)
azide-488 (μM)
CNP3
azide-488 in-gel fluor
coomassie
azide-AF647 (μM)
azide-AF647 in-gel fluor
E
DMSO
CNP7
CNP9
click with biotin azide
Streptavidin enrichment
WB analysis
F
CNP7
CNP9
Elutes (10%)
HMGCS1
ISOC1
Input

Fig S2. Profiling the reactivity of CNP7 and CNP9 in cells. (A) A workflow of direct affinity precipitation-mass spectrometry (AP-MS) following click chemistry. 293T cells treated with vehicle, CNP7, or CNP9 (0.5 μM) for 1 hour were lysed and subjected to a copper-mediated click reaction with biotin-azide. After streptavidin bead enrichment, the eluates were analyzed through tandem-mass-tag (TMT)-based proteomic analysis. (B) Volcano plots of the $-\log_{10}$-transformed p-value versus the $\log_2$-transformed ratio of CNP7/DMSO (left) or CNP9/DMSO (right) treated cells prepared as presented in panel a. n = 3 biological replicates. p-values were calculated by two-sided Welch's t-test (adjusted to 1% FDR for multiple comparisons, S0=1). (C) HEK293T cell lysates treated with DMSO or CNP7 (0.5 μM) for 4 hours underwent Cu (I)-mediated click reactions with varying TCEP concentrations; in-gel fluorescence was analyzed. (D) HEK293T cells treated with CNP3 (5 μM, 4h) were subjected to click reactions using varying amounts of azide-488 or azide-AF647 dyes, with TECP or sodium ascorbate. The in-gel fluorescence results displayed a markedly different pattern. (E) Workflow of AP-WB experiments involving cells treated with DMSO, CNP7, or CNP9 (0.5 μM for 1 hour), then subjected to click reaction with biotin-azide. (F) AP-WB results of the samples prepared as in panel E show ISOC1 enriched in the DMSO-treated fraction after click chemistry, indicating artificial enrichment through the click-mediated process. This is consistent with the AP-MS results shown in panel B.

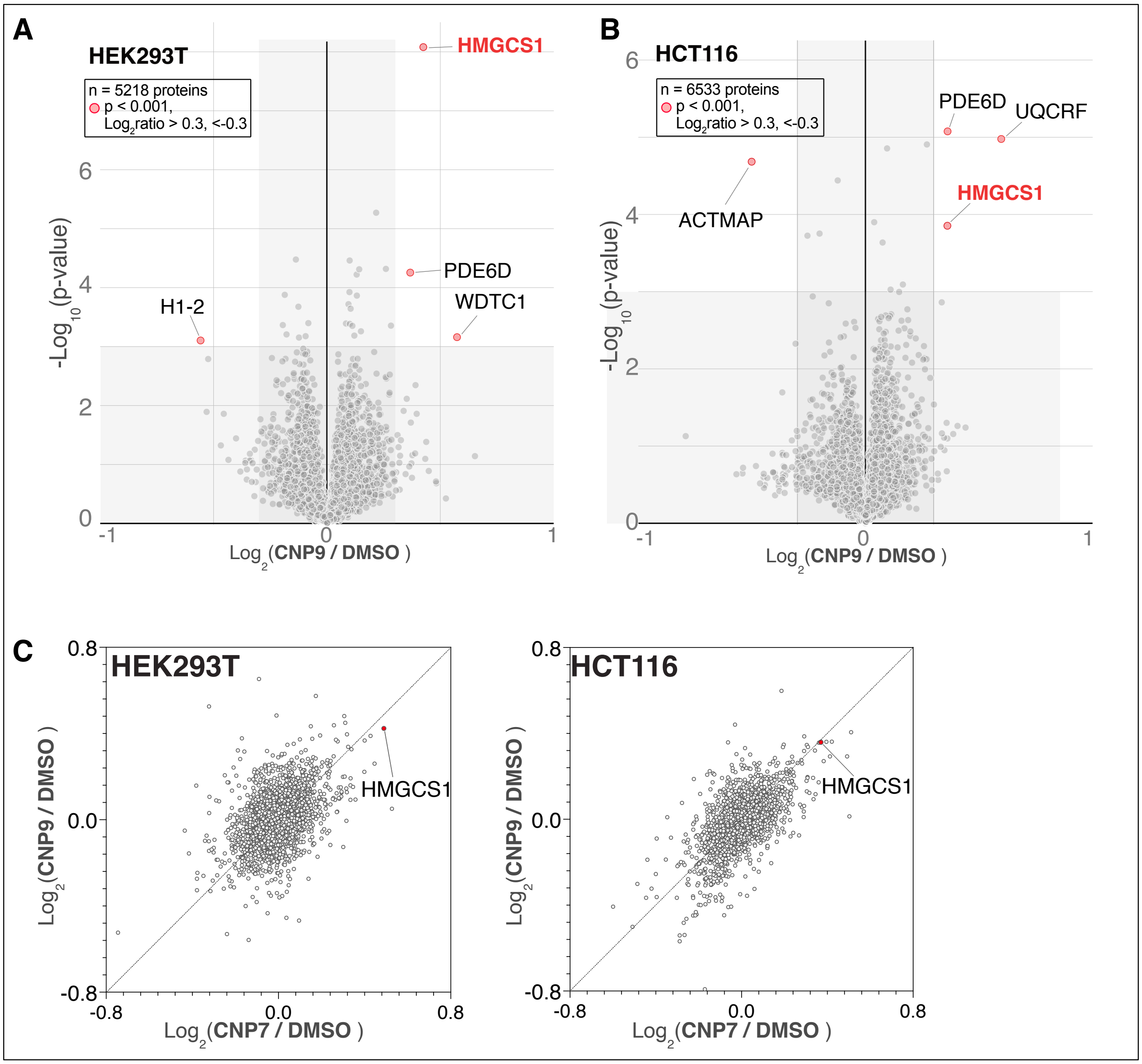

Fig S3. PISA assay upon CNP9 treatment. (A,B) Volcano plots of the –log10-transformed p-value versus the log2-transformed ratio of CNP9/DMSO analyzed from HEK293T cells are shown in panel A, and those for HCT116 are in panel B. n = 6 biological replicates for the DMSO-treated condition and 5 for the CNP7-treated condition. p-values were calculated by two-sided Welch's t-test (adjusted to 1% FDR for multiple comparisons, S0=0.285). (C) The plots of Log_2(CNP7/DMSO) versus Log_2(CNP9/DMSO) are shown in HEK293T on the left and HCT116 on the right.

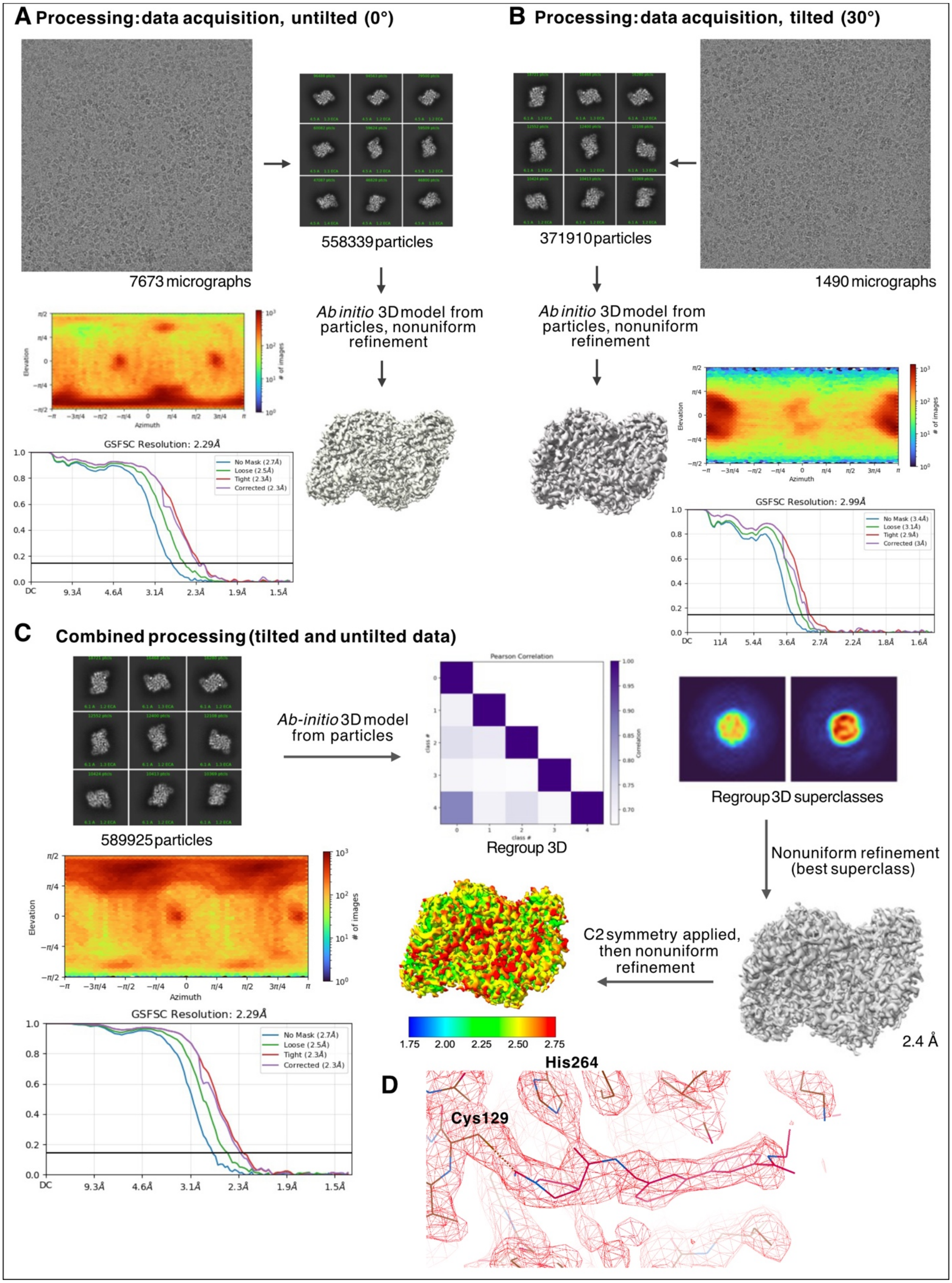
A Processing: data acquisition, untilted (0°)
7673 micrographs
558339 particles
Ab initio 3D model from particles, nonuniform refinement
Elevation
Azimuth
# of images
GSFSC Resolution: 2.29Å
No Mask (2.7Å)
Loose (2.5Å)
Tight (2.3Å)
Corrected (2.3Å)
DC
9.3Å
4.6Å
3.1Å
2.3Å
1.9Å
1.5Å
B Processing: data acquisition, tilted (30°)
371910 particles
1490 micrographs
Ab initio 3D model from particles, nonuniform refinement
GSFSC Resolution: 2.99Å
No Mask (3.4Å)
Loose (3.1Å)
Tight (2.9Å)
Corrected (3Å)
11Å
5.4Å
3.6Å
2.7Å
2.2Å
1.8Å
1.6Å
C Combined processing (tilted and untilted data)
589925 particles
Ab-initio 3D model from particles
Pearson Correlation
Regroup 3D
Regroup 3D superclasses
Nonuniform refinement (best superclass)
C2 symmetry applied, then nonuniform refinement
2.4 Å
1.75
2.00
2.25
2.50
2.75
GSFSC Resolution: 2.29Å
No Mask (2.7Å)
Loose (2.5Å)
Tight (2.3Å)
Corrected (2.3Å)
D
His264
Cys129

Fig S4. Cryo-EM data processing of the HMGCS1~CNP7 covalent adduct. (A,B) Processing scheme of the data collected with the untilted (0°, panel A) and tilted (30°, panel B) modes to compensate for orientation bias. After micrograph selection, particles were picked, classified, and refined into a 3D Coulomb potential map in CryoSPARC. (C) All classes from both angles were combined, yielding a 2.29 Å map (colored here according to local resolution). The Gold Standard Fourier Shell Correlation (GSFSC) curve and an angular distribution plot are displayed in each panel. (D) Density of CNP7 close to Cys129 before modeling.

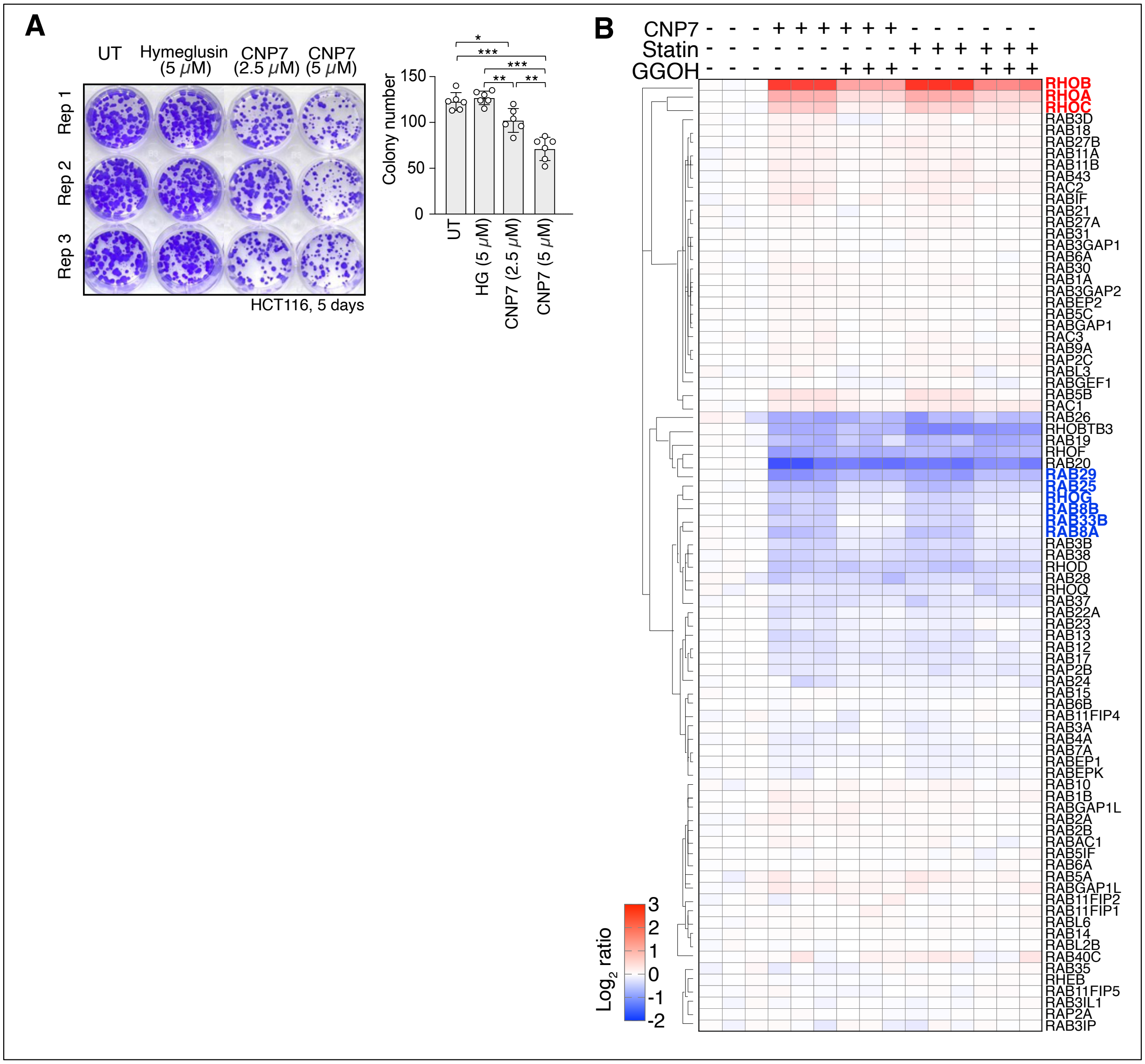

Fig S5. Cellular effects of CNP7 treatment. (A) CNP7 (2.5 µM or 5 µM) treatment for 5 days inhibits the colony-forming activity of HCT116 cells, whereas Hymeglusin (5 µM) shows no effect. Quantification is shown on the right. Colony numbers are presented as means ± s.d. (n = 6 biological replicates for each treatment). (B) Hierarchical clustering of isoprenylation substrates. Proteins that increase after CNP7 treatment and are reversed by GGOH supplementation are shown in red, while those that decrease after CNP7 treatment and are reversed by GGOH are shown in blue. CNP7 (5 µM, 24h), Simvastatin (10 µM, 24h), and/or GGOH (10 µM, 24h).

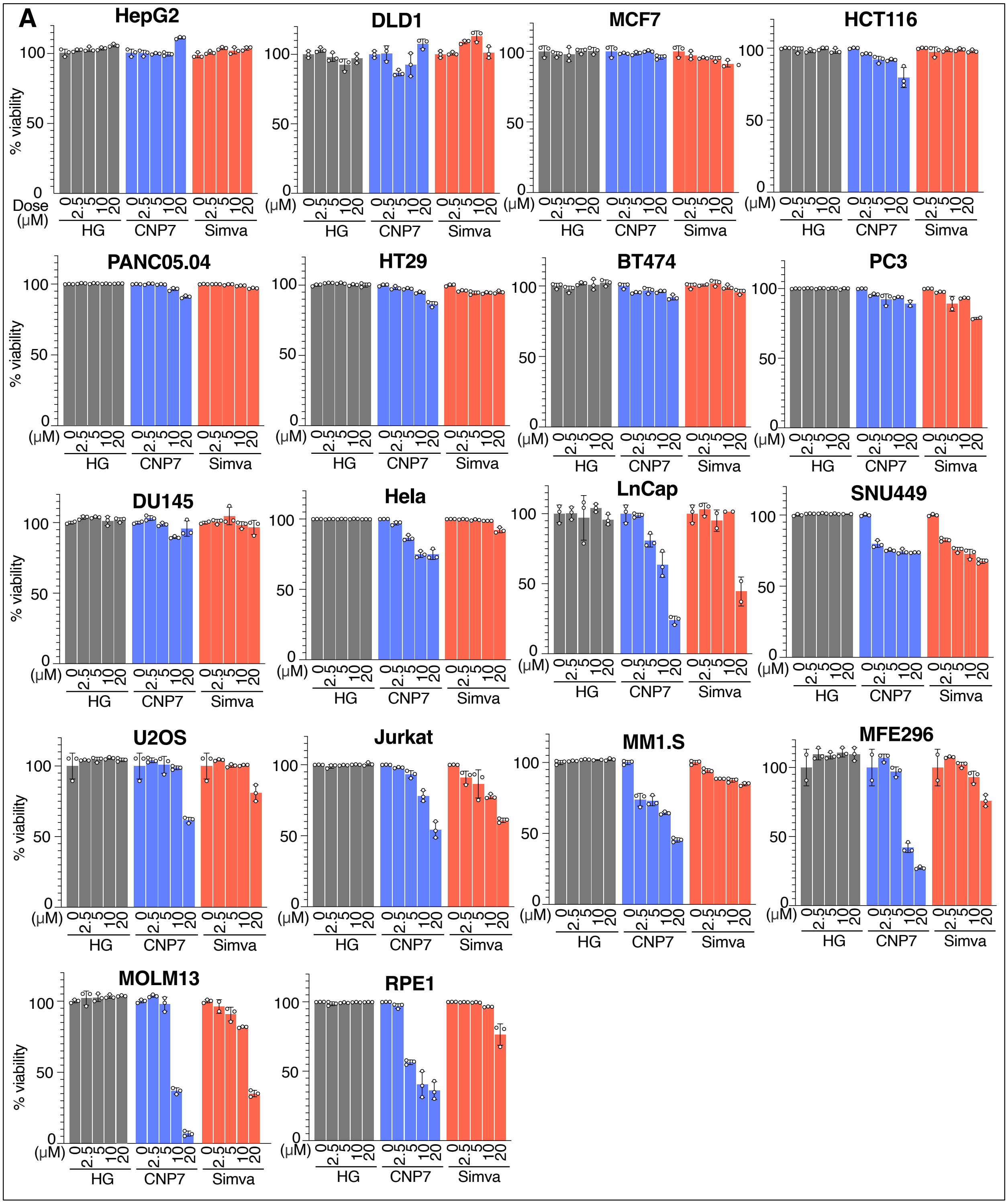

Fig S6. Cell line-specific anti-proliferative effect of CNP7. (A) Cell viability analysis on various cell lines after treatment with Hymeglusin, CNP7, or Simvastatin at the indicated concentrations for 72 hours. Means ± s.d. of biological triplicates.

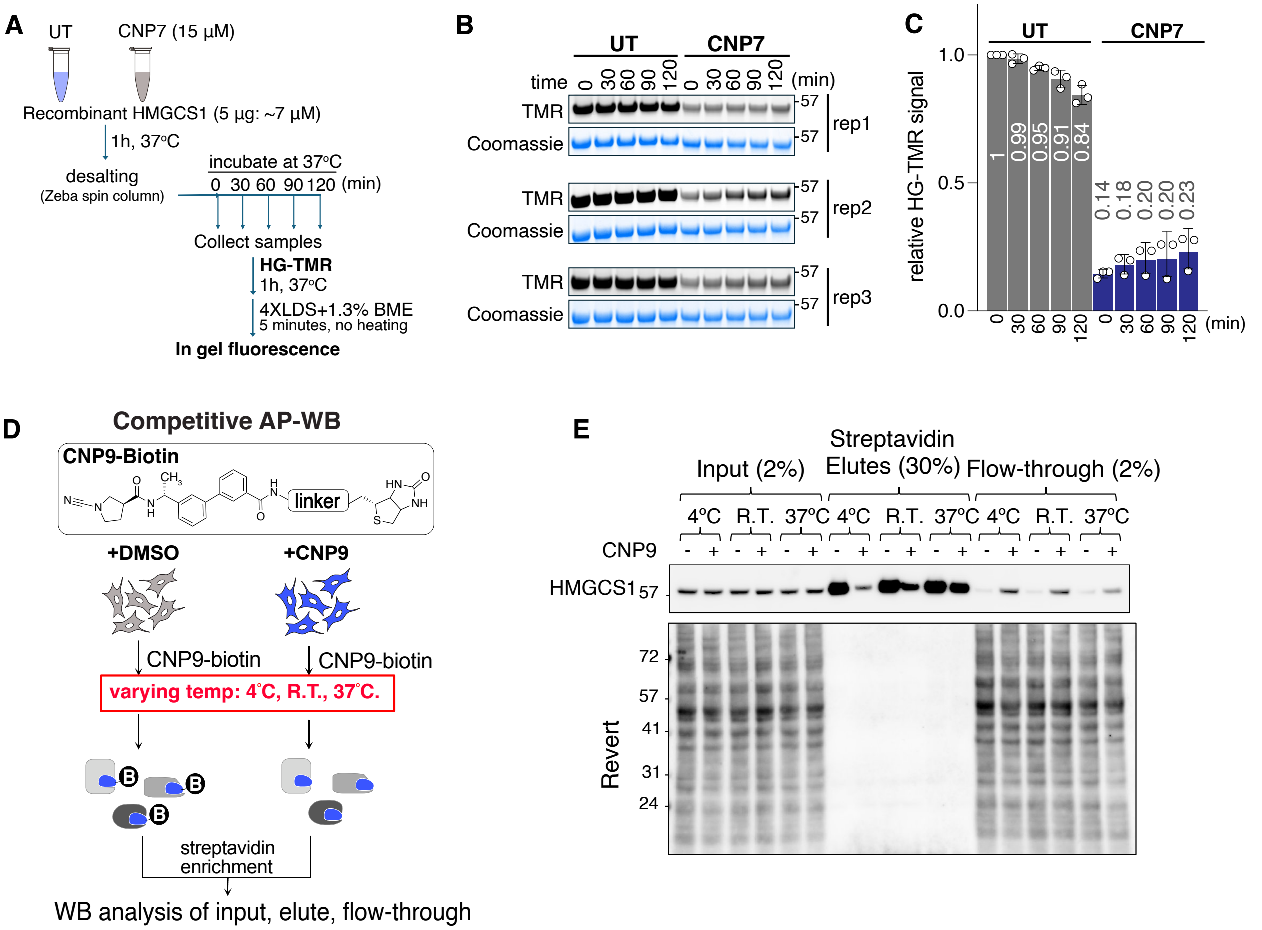

Fig S7. Reversibility of HMGCS1~CNP7 can influence cellular target engagement and impact chemoproteomic results. (A) Workflow of the HMGCS1~CNP7 reversibility test in vitro. (B) Samples from panel A were analyzed through in-gel fluorescence detection of the Hymeglusin-TMR signal. (C) TMR signal quantification from panel B. Three biological replicates (n = 3) are shown as mean ± s.d. Note: The loss of TMR signal in the UT condition over time was not corrected for normalization. (D) A workflow demonstrating reversibility during sample preparation. (E) Immunoblotting results of samples prepared as in panel D are presented. The HMGCS1 levels in the elute of CNP9-pretreated samples varied with the CNP9-biotin incubation temperature. This indicates that, at higher temperatures, the pre-existing HMGCS1~CNP9 complexes formed within the cells could have been reversed during the in vitro incubation.

Biological Methods

Cell lines

HEK293T (human embryonic kidney, fetus, ATCC CCL-3216, RRID: CVCL_0063), HCT116 (human colorectal carcinoma, male, ATCC CCL-247, RRID: CVCL_0291), MCF7 (human breast adenocarcinoma, female, ATCC HTB-22, RRID: CVCL_0031), U2OS (human osteosarcoma, female, ATCC HTB-96, RRID: CVCL_0042), DLD1 (human colorectal adenocarcinoma, male, ATCC CCL-221, RRID: CVCL_0248), HepG2 (human liver hepatocellular carcinoma, male, ATCC HB-8065, RRID: CVCL_0027), PC-3 (human prostate adenocarcinoma, male, ATCC CRL-1435, RRID: CVCL_0035) and MFE296 (human endometrial adenocarcinoma, female, generous gift from Neal Rosen, RRID: CVCL_1406), HT-29 (human colorectal adenocarcinoma, ATCC HTB-38, RRID: CVCL_0320), BT-474 (human breast ductal carcinoma, female, ATCC HTB-20, RRID: CVCL_0179), DU145 (human prostate carcinoma, male, ATCC HTB-81, RRID: CVCL_0105), hTERT RPE-1 (human retinal pigment epithelium, ATCC CRL-4000, RRID: CVCL_4388), and HeLa (human uterine adenocarcinoma, female, ATCC CRM-CCL-2, RRID: CVCL_0030) cells were grown in Dulbecco's modified Eagle's medium (DMEM, high glucose and pyruvate) supplemented with 10% fetal bovine serum and maintained in a 5 % CO_2 incubator at 37 °C. Panc 05.04 (human pancreatic adenocarcinoma, female, ATCC CRL-2557, RRID: CVCL_1637), MOLM13 (human acute myeloid leukemia, RRID: CVCL_2119), Jurkat cells (human acute T cell leukemia, male, ATCC TIB-152, RRID: CVCL_0367), MM.1S (human myeloma, female, ATCC CRL-2974, RRID: CVCL-8792), SNU449 (human liver hepatocellular carcinoma, ATCC CRL-2234, RRID: CVCL_0454), and LNCaP (human prostate carcinoma, male, ATCC CRL-1740, RRID: CVCL_0395) were grown in RPMI supplemented with 10% fetal bovine serum. All cell lines were found to be free of mycoplasma.

Antibodies and chemicals

The following antibodies and reagents were used in this study: HMGCS1 (36877S, Cell Signaling Technology), HMGCR (Ab242315, Abcam), HMG (ABS2108, Sigma Aldrich), phospho-S6 ribosomal protein Ser235,236 (4858S, Cell Signaling Technology), Tubulin (ab7291, Abcam), RhoA (Cell Signaling Technology, 2117T), SREBP2 (abcam, ab30682), trypsin (Promega, V511C), VCP (Sigma Aldrich, HPA012728), RIOK2 (Proteintech, 17410-1-AP), PDE6D (Proteintech, 228573-1-AP), pS6K (Cell Signaling Technology, 9234S), 4EBP1 (Cell Signaling Technology, 9644T), p-4EBP1 (Cell Signaling Technology, 2855T), TSC2 (Cell Signaling Technology, 43087T), PTEN (Cell Signaling Technology, 9559T), IRDye 800CW Goat anti-Rabbit IgG H+L (926-32211, LI-COR), IRDye 800CW Goat anti-Mouse IgG H+L (926-32210, LI-COR), IRDye 680 RD Goat anti-Mouse IgG H+L (926-68070, LI-COR), Bio-Rad Protein Assay Dye Reagent Concentrate (Bio-Rad,#5000006), Lipofectamine 3000 (L3000008, Invitrogen), GGTi298 (Selleck Chemicals, S7466), GGOH (Cayman Chemical Company, 13272), Simvastatin (11-101-5563, Fisher Scientific), Hymeglusin (11899, Cayman Chemical), Benzonase Nuclease HC (71205-3, Millipore), REVERT™ Total Protein Stain kit (LI-COR, P/N926-11010), 1,1,1,3,3,3-Hexafluoro-2-propanol (52517, Sigma), sodium dodecyl sulfate (SDS) (Fisher Scientific, PI28364), hydroxylamine solution (Sigma Cat#438227), chloroacetamide (Fisher Scientific, AAA1523830), Sodium pyruvate (Gibco, 11360-070), cycloheximide (Fisher Scientific, AC357420050). TCEP (Gold Biotechnology), Formic acid (Sigma Aldrich, 94318), TMTpro 18plex Label Reagent (Fisher Scientific, A52045), TMT6 plex (Thermofisher, 90068), InstantBlue (Abcam, ab119211), GK16S (MedChem Express, HY-154914), Biotin-PEG2-azide (MedChem Express, HY-126957), Tris[(1-benzyl-1H-1,2,3-triazol-4-yl)methyl]amine (TBTA, Sigma-

Aldrich, 678937), Carboxyrhodamine 110 Azide, 5-TAMRA Alkyne (CCT-1255), High-capacity streptavidin agarose beads (Pierce, 20361).

Generation of HEK293T cells over-expressing WT and C129A HMGCS1-V5

Stable cell lines were generated according to the previously reported method.[1] HEK293T cells were transfected with pHAGE-HMGCS1-V5 or pHAGE-HMGCS1 (C129A)-V5. Virus-containing media were collected 24 and 48 hours after transfection and filtered with 0.45 μm filters. HEK293T cells were infected with the viral solution, and puromycin selection was performed.

Cell lysis and immunoblotting assay

Corresponding cells were incubated with the corresponding chemicals to ~50-60% confluency. After removing the media, the cells were washed with DPBS twice, then lysed with RIPA buffer or 0.5% NP-40 buffer (50 mM HEPES HCl, 150 mM NaCl, pH 7.4) containing protease inhibitors, 200 μM TCEP, 250 mM $MgCl_2$, and 20 units/ml Benzonase (Millipore, for RIPA buffer only) were added directly onto the cells. The lysates were sonicated (with RIPA buffer only) and centrifuged, and then the concentration of the supernatant was measured by Bradford assay. The whole cell lysate was denatured by the addition of LDS sample buffer supplemented with 50 mM DTT, followed by boiling at 90 °C for 5 minutes. To detect HMGCR and HMG-lysine, the addition of DTT and the boiling steps were omitted. 20 or 30 μg of each lysate (60 or 70 μg for HMGCR and HMG) was loaded onto the 4-12% NuPAGE Bis-Tris gel (Thermo Fisher Scientific), followed by SDS-PAGE with MES SDS running buffer (Thermo Fisher Scientific). The proteins were electro-transferred to PVDF membranes (0.45 μm, Millipore), and then the total protein was stained by Revert total protein stain kit (LI-COR) or Ponceau staining (Thermo Fisher Scientific).

The membrane was then blocked with 5% non-fat milk, incubated with the indicated primary antibodies (4 °C, overnight), washed three times with TBST (total 15 min), and further incubated with fluorescent IRDye secondary antibody (1:20000) for 1 h at room temperature. After three washes with TBST for 15 min, the near-infrared signal was detected using a Chemidoc MP (Biorad) and quantified using Image Lab.

Affinity-purification and scavenging proteomics sample preparation (Fig. 3 and Fig. S2)

For the direct affinity precipitation-mass spectrometry (AP-MS), wildtype HEK293T cells were plated on a 10 cm dish per replicate condition and treated with 0.5 μM of CNP7 or CNP9 for 1 h. Cells were washed with ice-cold PBS three times and lysed with 300 μl of 0.5% NP-40 buffer (50 mM HEPES HCl, 150 mM NaCl, pH 7.4). The lysates were centrifuged (7000 rpm, 1 min), and the protein concentration of the supernatant was measured by Bradford assay reagent. 1 mg of the protein was incubated with click reagents (final concentration: 1 mM $CuSO_4$, 100 mM TBTA ligand, 2 mM TCEP, and 100 μM biotin-azide) for 1 h while rocking at room temperature. MeOH/$CHCl_3$ precipitation was performed, and the precipitated protein disk was resuspended in 200 μl of 1% SDS (50 mM HEPES HCl, 150 mM NaCl, pH 7.4), sonicated, and diluted by adding 600 μl of HEPES-NP-40 buffer (final SDS concentration is < 0.3%). Then, each lysate was added to 15 μl of high-capacity streptavidin beads (Pierce™ High-Capacity Streptavidin Agarose) and incubated for 2 h at 4°C. Input and flow-through (2%) were subjected to the quality control immunoblots and scavenging proteomics. The beads were washed once with HEPES-NP-40 buffer, twice with PBS, and then transferred onto hydrophilic PTFE membrane filter cups (Millipore, UFC30LG25). They were washed twice with water. Completely dried beads were resuspended in 50 μl of hexafluoroisopropanol (HFIP), incubated for 5 min with shaking (500 rpm), and eluted

by centrifugation at 4000 rpm. This elution step was repeated once, and the eluates were combined and dried in a SpeedVac. Trypsin digestion and TMT labeling, followed by reduction and alkylation, were performed as described in the total proteomics analysis section. The competitive AP-MS was performed according to the direct AP-MS protocol, but the cell lysates were incubated with CNP7-biotin or CNP9-biotin (final concentration, 5 μM) for 1 h at 4 °C instead of the click reagents. For the scavenging proteomics, 125 μg of input and flow-through after streptavidin enrichment were subjected to the TMT-based analysis.

Native cysteome profiling sample preparation (Fig. 6D)

HEK293T cells were seeded onto 8 x 10 cm dishes 24 hours before treatment with DMSO or CNP7. On the following day, DMSO (0.1% final) or CNP7 (0.5 μM final) was added to the cells, and the cells were incubated for 4 hours. The cells were then collected by gently pipetting them up in cold DPBS on ice, followed by centrifugation at 4,000 rpm for 1 minute. The cell pellets were washed again with DPBS, then 700 μL of DPBS containing Benzonase and 5 mM $MgCl_2$ was added, followed by sonication once with a tip sonicator. Complete cell lysis was confirmed by observing nuclei with an inverted microscope after trypan blue staining. After the Bradford assay, 350 μg of protein from each sample was diluted to a final volume of 350 μl with DPBS, yielding a protein concentration of 1 mg/ml. Then, 7 μl of 10 mM biotin-iodoacetamide stock solution was added to achieve a final concentration of 200 μM, and the mixture was incubated in the dark for 1 hour on a tumbling rotor. The reaction mixture was then incubated with DTT (10 mM final) in the dark for 15 minutes, after which 25 mM (final concentration) iodoacetamide was added and incubated for another 15 minutes. Proteins were then precipitated by mixing in a 4:4:1 ratio of H_2O: MeOH: Chloroform, followed by vigorous vortexing and centrifugation at 13,000 rpm for 5 minutes. The

protein disc was rinsed with cold methanol, dried, and digested overnight at 37 °C on a thermomixer with trypsin (1:175 trypsin-to-protein ratio) in 175 µL EPPS buffer (200 mM, pH 8.5). Complete digestion was confirmed by running an SDS-PAGE gel of a small aliquot (20 µg) of the reaction mixture. The samples were then diluted with RIPA to achieve a total volume of 1 mL (330 µg/mL peptide concentration), incubated with 10 µL of high-capacity streptavidin beads (Pierce) per sample for 2 hours at room temperature, and washed three times with RIPA buffer. The complete enrichment of biotin-labeled peptides was confirmed by comparing the input and flow-through fractions using dot blotting with streptavidin-IRD800 conjugate. After confirmation, the biotin-labeled peptides were eluted into a Lobind tube using 50 µL of HFIP, performed twice. The combined eluate was then dried in a SpeedVac, resuspended in 20 µL of EPPS, and labeled with 5 µL of TMT reagents for 1 hour. The sample was then quenched with 1% NH_2OH. The remaining steps are similar to those for TMT-proteomic sample preparation, and the sample was fractionated according to the manufacturer's instructions using the High pH reversed-phase peptide fractionation kit (Pierce Cat# 84868) to produce six fractions. It was then subjected to C18 StageTip desalting before MS analysis.

Sample preparation for TMT/TMTpro-based proteomics analysis

The total proteomics analyses were performed based on the previously reported method.[1] Briefly, protein samples were reduced by incubation with 5 mM TCEP at 55 ºC for 10 min. After cooling down to room temperature, the lysates were reacted with chloroacetamide solution (final conc. 20 mM) at room temperature for 15 min, followed by chloroform/methanol precipitation (Fig. 3F and Fig. 7).[2] Samples were resuspended in 100 mM EPPS (pH 8.5) containing 0.1% RapiGest and digested at 37ºC overnight with Trypsin (100:1 protein-to-protease ratio). Tandem mass tag

labeling of each sample was performed by adding the reagent along with acetonitrile to achieve a final acetonitrile concentration of approximately 30% (v/v). Following incubation at room temperature for 1 h, the reaction was quenched with hydroxylamine to a final concentration of 1% (v/v) for 15 min. The TMT-labeled samples were pooled together at a 1:1 ratio. For Fig. 3B and Fig. S2B, HFIP in the eluates was evaporated, followed by trypsin digestion, TMT labeling, and quenching with hydroxylamine. The TMT-labeled tryptic peptides were then reacted with TCEP (5 mM, 10 min) in this case and chloroacetamide solution (final concentration 20 mM) at room temperature for 15 min. The samples were vacuum centrifuged to near dryness, subjected to C18 solid-phase extraction, dried again via vacuum centrifugation, and reconstituted in 5% acetonitrile, 1% formic acid for LC-MS/MS analysis. For Fig. 7, the dried TMTpro-labeled samples were resuspended in 100 μL of 10 mM NH_4HCO_3, pH 8.0, and off-line fractionated using basic pH reversed-phase High-performance liquid chromatography (HPLC) (Agilent LC1260 Infinity II, equipped with a degasser and wavelength detector (set at 214 nm)).[3] Peptides were subjected to a 60 min linear gradient from 8% to 40% acetonitrile in 10 mM NH_4HCO_3, pH 8.0, at a flow rate of 0.6 mL/min over an Aeris peptide XB C18 column (Phenomenex; 2.6 μm, 100 Å, 4.6 mm ID and 250 mm in length). The 96 resulting fractions were then pooled in a non-continuous manner into 24 fractions for mass spectrometry analysis. Fractions were vacuum centrifuged to near dryness. Each consolidated fraction was desalted using a Stage Tip, dried again via vacuum centrifugation, and reconstituted in 5% acetonitrile and 1% formic acid for LC-MS/MS processing.

Whole cell lysate-based PISA assays (Fig. 4)

The PISA proteomics analyses were performed based on the previously reported lysate-based assay methods.[4-6] HEK293T or HCT116 cells were grown in DMEM with 10% iron-fortified

bovine calf serum and 1% Pen/Strep, kept in a 5% CO_2 incubator at 37ºC. Cells on 10 cm plates were harvested at 80% confluency using trypsin. They were then washed twice with PBS, collected as cell pellets, and snap-frozen in liquid nitrogen. Before the PISA assay, the cells were resuspended in lysis buffer (200 mM EPPS, 150 mM NaCl, 5 mM EGTA, and protease inhibitors, pH 7.2), then lysed by multiple freeze-thaw cycles in liquid nitrogen and by vortexing to shear DNA. The lysate was cleared by centrifugation for 30 min at 21,130 x g. The supernatant was collected, kept on ice, and its concentration was measured by Bradford assay. Finally, the lysate was diluted to 2 mg/mL with lysis buffer and divided into aliquots.

Treatment buffers were prepared by adding 10 mM stock solutions of CNP7 or CNP9 to the lysis buffer (20 µM) or to an equal volume of DMSO for controls, and were then kept on ice. Each protein lysate sample was mixed 1:1 with the corresponding treatment buffer, incubated at room temperature for 15 minutes, and then cooled on ice. For each condition, 30 µL of lysate or buffer was aliquoted into 10 PCR tubes, organized into sets of ten separated strips. Each strip was placed in a BioRad C1000 Touch thermal cycler at 21 ºC for 3 minutes, then subjected to a 3-minute incubation at a specific temperature, across a temperature gradient from 42 to 60 ºC in 2 ºC steps. Afterward, the samples were equilibrated at 21 ºC for an additional 3 minutes. From each set of ten tubes, 25 µL was collected and pooled into a protein LoBind tube, yielding 16 samples, which were centrifuged for 1 hour at 21,130 x g. Finally, 50 µL of each supernatant was immediately frozen at -20 ºC overnight.

PISA sample preparation and TMTpro labeling

Each sample was reduced with a final concentration of 5 mM TCEP for 10 minutes at 55ºC, then alkylated using 20 mM iodoacetamide for 10 minutes at room temperature in the dark. The reduced and alkylated samples were subsequently desalted using SP3 beads (Cytiva).[2] Bead-bound proteins were digested in a solution containing 0.1 M EPPS, 0.1% RapiGest, with 0.75 µg of Trypsin overnight at 37ºC on a thermomixer set to 1200 rpm. Half of the digested sample was transferred to a protein LoBind tube, where TMTpro labeling was then performed. After labeling at room temperature for 1 hour, a ratio check was conducted, followed by quenching the samples with 0.5% hydroxylamine for 10 minutes at room temperature. The TMTpro-labeled samples were combined at a 1:1 ratio. The sample was vacuum-centrifuged to near dryness, then subjected to C18 solid-phase extraction and dried again. The dried TMTpro-labeled sample was resuspended in 100 µL of 10 mM NH_4HCO_3, pH 8.0, and off-line fractionated using basic pH reversed-phase High-performance liquid chromatography (HPLC) (Agilent LC1260 Infinity II, equipped with a degasser and wavelength detector (set at 214 nm)). Peptides were subjected to a 60 min linear gradient from 8% to 40% acetonitrile in 10 mM NH_4HCO_3, pH 8.0, at a flow rate of 0.6 mL/min over an Aeris peptide xb C18 column (Phenomenex; 2.6 µm, 100 Å, 4.6 mm ID and 250 mm in length). The 96 resulting fractions were then pooled into 18 fractions for mass spectrometry analysis. Fractions were vacuum centrifuged to near dryness. Each consolidated fraction was desalted via Stage Tip, dried again via vacuum centrifugation, and reconstituted in 5% acetonitrile, 1% formic acid for LC-MS/MS processing.

Mass spectrometry data acquisition

Mass spectrometry data were acquired using an Orbitrap Eclipse Tribrid mass spectrometer (Thermo Fisher Scientific, San Jose, CA) connected to an UltiMate 3000 RSLCnano UHPLC

(Thermo Fisher Scientific, San Jose, CA). Peptides were separated on a 100 µm inner diameter microcapillary column packed in-house with ~ 30 cm of HALO Peptide ES-C18 resin (2.7 µm, 160 Å, Advanced Materials Technology, Wilmington, DE) with a gradient consisting of 5 %-40 % (ACN, 0.1% FA). 3/10 of each fraction was loaded onto the column for analysis. Proteome analysis used Multi-Notch MS^3-based TMT quantification[7] to reduce ion interference compared to MS^2 quantification[8], combined with the FAIMS Pro Interface (using previously optimized 3 CV parameters for TMT multiplexed samples[9]), and integrated with newly implemented Real-Time Search analysis software.[10, 11] The scan sequence began with an MS^1 spectrum (Orbitrap analysis; resolution 120,000 at 200 Th; mass range 400−1500 m/z; maximum injection time 50 ms; automatic gain control (AGC) target) $4x10^5$). For MS^2 analysis, precursors were selected based on a cycle time of 1.25 sec/CV method (FAIMS CV=-40/-60/-80). MS^2 analysis consisted of collision-induced dissociation (quadrupole ion trap analysis; Rapid scan rate; AGC $1.0x10^4$; isolation window 0.5 Th; normalized collision energy (NCE) 35; maximum injection time 35 ms). Monoisotopic peak assignment was used, and previously interrogated precursors were excluded using a dynamic window (180 s ±10 ppm). Following the acquisition of each MS^2 spectrum, a synchronous-precursor-selection (SPS) API-MS^3 scan was collected on the top 10 most intense ions b or y-ions matched by the online search algorithm in the associated MS^2 spectrum.[10, 11] MS^3 precursors were fragmented by high-energy collision-induced dissociation (HCD) and analyzed using the Orbitrap (NCE 45 (TMTpro); NCE 65 (TMT); AGC $2.5x10^5$; maximum injection time 200 ms, resolution was 50,000 (or 15000 for Fig. 3B right, and Fig. S2B) at 200 Th). Closeout was set at three peptides per protein (or two peptides per protein per fraction for Fig. 7), so that MS^3s were no longer collected for proteins having two peptide spectrum matches (PSMs) that passed

quality filters.[11] Cystome profiling data (Fig. 6D) were acquired using the double-shot analysis method.[12]

Proteomics Data Analysis

Mass spectra were processed using a Comet-based (2020.01 rev. 4) software pipeline.[13, 14] Spectra were first converted to mzXML, and monoisotopic peaks were re-assigned using Monocle software.[15] MS^2 spectra were matched with peptide sequences with a composite sequence database including the Human Reference Proteome UniProt database (Proteome ID: UP000005640, release 2024_05, containing canonical and isoform entries), as well as sequences of common contaminants. This database was concatenated with one composed of all protein sequences in the reverse order. Analysis was performed using a 50 ppm precursor ion tolerance. Static modifications included TMTpro tags (+304.207 Da) (or TMT tags (+229.163 Da) for Fig. 3B, S3, and 6E) on lysine residues and peptide N termini, along with carbamidomethylation of cysteine residues (+57.021 Da). Oxidation of methionine residues (+15.995 Da) was set as a variable modification. For Fig. 6E, an additional biotin alkyne modification of Cysteine residues was included, adding +357.172 Da. Peptide-spectrum matches (PSMs) were adjusted to a 1% false discovery rate (FDR).[16] PSM filtering was performed using a linear discriminant analysis[17], while considering the following parameters: Comet log expect, different sequence delta Comet log expect, missed cleavages, peptide length, charge state, precursor mass accuracy, and fraction of ions matched. For protein-level comparisons, PSMs were identified, quantified, and collapsed to a 1% peptide false discovery rate (FDR) and then collapsed further to a final protein-level FDR of 1%.[18] To generate the smallest set of proteins required to account for all observed peptides, the principles of parsimony were applied. For TMT-based reporter ion quantitation, the summed signal-to-noise (S:N) ratio for each

TMT channel was first extracted based on the closest matching centroid to the expected mass of the TMT reporter ion (integration tolerance of 0.003 Da, or 0.03 for Fig. 3B, and Fig. S3B). Isotopic impurities of the different TMT and TMTpro reagents provided by the manufacturer's specifications were used to adjust reporter ion intensities. Proteins were quantified by summing reporter ion signal-to-noise measurements across all matching PSMs, resulting in a ''summed signal-to-noise'' measurement. PSMs with poor quality, MS^3 spectra missing reporter ion channels, isolation specificity below 0.5, or TMT reporter summed signal-to-noise ratio under an average of 10 per sample, or with no MS^3 spectra, were excluded from quantification.

Protein quantification values were exported for further analysis in Microsoft Excel and Perseus.[19] In Perseus, a two-way Welch's t-test analysis was performed to compare two datasets, using the S0 parameter of 0.585 for Fig. 7, 0.285 for Fig. 4 and Fig. S3, or of 1 for the rest proteomic datasets as a minimal fold change cutoff, and correction for multiple comparisons was achieved using the permutation-based FDR method, both of which are built-in functions of Perseus software. For AP-MS (Fig. 3B and Fig. S3B), each TMT 6-plex channel was normalized to the streptavidin ion intensity, which was eluted after the HFIP treatment. For whole-cell (Fig. 7), PISA (Fig. 4 and Fig. S3), Cysteome (Fig. 6D), or flow-through (Fig. 3F) proteome analyses, each reporter ion channel was summed across all quantified proteins and normalized, assuming equal protein loading across all samples.

Supplementary Tables list all quantified proteins and the TMT reporter ratio associated with control channels used for quantitative analysis. The MS TMT proteomic data have been deposited

in the MassIVE repository under the dataset identifier MSV000099720. (TEMPORARY REVIEWER ACCESS | Username: “MSV000099720 _reviewer”; Password: “LiangYi_2025”)

Gene ontology analysis (Fig. 7)

Enrichment analysis of significantly upregulated proteins by CNP7 treatment was conducted by adding the protein lists to the Enrichr web server (https://maayanlab.cloud/Enrichr/). Among the gene set libraries in Enrichr, ‘GO Biological Process 2023’ and ‘GO Molecular Function 2023’ were curated with –log P-value.

Recombinant protein purification

For bacterial expression of HMGCS1 proteins, BL21 cells were transformed with the pNIC28-Bsa4-6xHis-TEV-HMGCS1(aa16-455) vector and cultured in 1 L of Terrific broth at 37 °C. When the OD600 reached ~1.0, the protein was induced with 0.5 mM IPTG overnight at 18 °C. Harvested cells were homogenized in lysis buffer (50 mM HEPES, pH 7.5, 150 mM NaCl, 1% Triton-X, 0.06% BME, 1 tablet of protease inhibitor) and centrifuged at 15000 g. The supernatant was applied to the Ni-NTA resin (1 ml) and washed with high-salt buffer (50 mM HEPES, 500 mM NaCl, 1 M urea, 20 mM imidazole, pH 7.5) and low-salt buffer (50 mM HEPES, 150 mM NaCl, pH 7.5). The protein was then eluted with elution buffer (50 mM HEPES, 150 mM NaCl, 500 mM imidazole, pH 7.5). The eluent was applied to a Superdex 75 column, pre-equilibrated with a low-salt buffer. The final protein yield was 5 mg/L of culture. For cryo-EM sample preparation, 50 mL of a 1 L bacterial culture was harvested, and the protein was purified as described above. The concentration of the SEC-purified protein was determined using a Nanodrop spectrophotometer, and a final concentration of 1 mg/mL was selected for CNP7 labeling. For labeling, 200 μL of the

1 mg/mL protein solution was incubated with 50 µM CNP7 at 37 °C for 1 h. Subsequently, 10 µL of the labeled protein was incubated with 85 µM HG-FL for 30 minutes to assess small-molecule labeling efficiency. After confirming that more than 90% of HMGCS1 was labeled by CNP7, the sample was immediately subjected to Cryo-EM grid preparation.

Cryo-EM grid preparation and data acquisition

TEM grids of the HMGCS1~CNP7 complex were prepared by applying 3 µL of protein/CNP7 conjugate to UltrAuFoil 300 mesh R1.2/1.3 grids (Quantifoil Micro Tools GmbH, Jena, Germany), which were plasma-cleaned for 120 s at 0.37 mbar and 15 mA in a 'Pelco easiGlow' glow discharge system (Ted Pella, Inc., Redding, CA). Grids were blotted for 3 s with 100% humidity at 4°C, then flash-frozen in liquid ethane using a Vitrobot Mark IV (Thermo Fisher Scientific, Eindhoven, Netherlands). Images were collected using a Krios G4 transmission electron microscope (Thermo Fisher Scientific) with a spherical aberration coefficient of 2.7 mm and operated at 300 kV. The microscope was equipped with a Selectris X (Thermo Fisher Scientific) energy filter and operated using a slit width of 10 eV. Images were recorded on a Falcon 4i (Thermo Fisher Scientific) direct electron detector using the EPU software platform (Thermo Fisher Scientific), with exposures collected at a defocus every 0.1 µm within a defocus range of -0.6 to -1.7 µm. 9163 micrographs were collected at an electron dose rate of 11.6 e^{-} $pixel^{-1}$ s^{-1} with a total exposure time of 2.93 s, giving a total exposure dose of 60 e^{-} $Å^{-2}$ s^{-1}.

Cryo-EM image processing

EER images from both tilted and untilted data collection sessions were processed in CryoSPARC version 4.7.1.[20] CryoSPARC Live was used for drift correction, CTF fitting, exposure curation,

particle picking and extraction, and initial 2D classification. Particles were extracted from micrographs at a box size of 300 pixels and Fourier-cropped to 100 pixels. Further 2D classification and heterogeneous ab-initio 3D map generation were performed on curated particles in CryoSPARC workspaces. The best particles from ab-initio 3D classes were then unbinned and used as input to non-uniform refinement jobs to obtain high resolution maps. For untilted data, 558339 curated particles yielded a 2.29 Å map; for tilted data, 371910 particles yielded a 2.99 Å map. These two particle sets were combined in a new workspace and re-extracted at a 256-pixel box size. Subsequent 2D classification and heterogeneous ab-initio reconstruction jobs yielded five 3D classes. The resulting classes appeared similar, so they were subjected to a Rebalance 3D job in order to obtain two superclasses. The particle set and volume from the best of these two superclasses were further refined to produce a 2.4 Å map from 589925 particles. Since the HMGCS1 complex is a homodimer, C2 symmetry was applied using CryoSPARC's Volume Alignments Tool, then refined in a final non-uniform refinement job into a 2.29 Å map. All map resolutions were estimated in CryoSPARC using the Fourier shell correlation criterion of 0.143.[21] Cryo-EM structural study-related data is deposited to the RCSB Protein Data Bank (RCSB PDB), dataset ID: D_1000302134 and PDB ID: 9ZAW.

Model building and refinement

PDB ID: 2P8U was chosen as the base model, where all extra chains were deleted, leaving only the two dimer chains of HMCGS1. The model and restraints files for the ligand were generated using Phenix eLBOW.[22, 23] The protein model was docked into the map using UCSF ChimeraX.[24, 25] Coot [26] was used to dock the ligand and create the protein-ligand linkage. Real-space refinement

of the model with respect to the map and comprehensive validation of the final structure were performed in Phenix.[22]

In-gel fluorescence analysis using Hymeglusin-fluorescence (HG-FL)

HG-FL-mediated in-gel fluorescence assay was performed based on the previously reported method.[27] Briefly, 1 μM recombinant HMGCS1 protein or cell lysates (30 or 35 μg in 20 μL NP40 lysis buffer) were incubated with HG-FITC (5 μM final) at 4 °C for 1 h, followed by SDS-PAGE, and the fluorescent signal was detected by Chemidoc MP (Biorad).

Screening of CNP molecules for recombinant HMGCS1 labeling (Fig. 1)

Each CNP compound was prepared as a 1:2 serial dilution (10, 20, 40, 80, and 160 μM) from a 5 mM stock. For each condition, 1 μL of the CNP dilution was mixed with 9 μL recombinant HMGCS1 (1 μM) and incubated for 1 h at 37°C. HG-FL (1 μL of 50 μM) was then added, and the mixture was incubated for 30 min at 37°C. Samples were resolved by SDS-PAGE, and in-gel fluorescence was imaged using a ChemiDoc MP (Bio-Rad).

Dual activity-based profiling assay (Fig. 2)

HEK293T cells were seeded in 6-well plates and allowed to adhere for 24 h. The next day, cells were treated with either 0.1% (v/v) DMSO or 0.5 μM CNP derivatives and incubated for 4 h; for time-course experiments, cells were harvested at the indicated time points. Cells were collected by gentle resuspension in ice-cold DPBS and pelleted by centrifugation at 1,500 rpm for 3 min. Pellets were lysed in buffer (50 mM HEPES, 150 mM NaCl, pH 7.5, 0.25% NP-40) supplemented with protease inhibitors. Protein concentration was determined by Bradford assay and normalized to 2

mg mL^{-1}. Normalized lysates were split into two portions. One portion was analyzed by in-gel fluorescence using HG-FL as described above. The second portion was subjected to the Copper(I)-catalyzed Azide-Alkyne Cycloaddition (CuAAC or "click") reaction with either TMR-azide or fluorescein (FL)-azide under the following final conditions: 1 mM $CuSO_4$, 2 mM TCEP, 100 μM TBTA, and 100 μM azide dye; reactions were incubated for 1 h at room temperature. Click reactions were quenched with 4× LDS sample buffer containing 1.25% β-mercaptoethanol (BME) and incubated for 5 min at room temperature. Samples were resolved by SDS-PAGE, and in-gel fluorescence was recorded on a ChemiDoc MP system (Bio-Rad). To detect HMGCS1, the proteins were electro-transferred to PVDF membranes (0.45 μm, Millipore), and then the total protein was stained by Revert total protein stain kit (LI-COR) or Ponseau S solution. The membrane was then blocked with 5% non-fat milk, incubated with the indicated primary antibodies (4°C, overnight), washed three times with TBST (total 15 min), and further incubated with fluorescent IRDye secondary antibody (1:20000) for 1 h at room temperature. After a wash with TBST for 15 min, the near-infrared signal was detected using a Chemidoc MP (Biorad)

Cell viability assay

Cell viability was evaluated using DAPI staining (MedChemExpress) and flow cytometry. Each respective cell line was cultured as described above and plated in triplicate per drug dose in 24-well plates. After the 48 or 72-hour treatment period, cells were harvested using trypsin, resuspended in sorting buffer (1X DPBS, 1 mM EDTA, 25 mM HEPES, 1% FBS, final pH 7.3-7.5) with 0.125 μg/mL DAPI, and filtered through a 35 μm cell strainer cap. Flow cytometry was performed on either the SONY SH800 (Sony Biotechnology) or the LSR Fortessa (BD

Biosciences), and analysis was done using the FlowJo software. Graphs were processed in Prism after exporting cells' median intensity values.

Colony formation assay

To evaluate the ability of single cancer cells to form colonies, cells were plated at 1,500 cells/well in 6-well plates (day 0). At day 5 and day 8, the cells were treated with the corresponding chemicals. At day 10, the cells were stained with a crystal violet working solution, which contains 0.5% crystal violet and 4% paraformaldehyde in PBS. Numbers of colonies were counted for each well ($n = 2$ or 3) and presented as mean ± s.d.

Statistical analysis

P values in the volcano plots of the proteomics data were analyzed by a two-sided Welch's t-test, which was adjusted for multiple comparisons. Comparisons of the quantified data for immunoblotting, colony formation were performed by unpaired Student's t-test; Statistical significance was judged based on p-values; $*p < 0.05$; $**p < 0.01$; $***p < 0.001$.

Synthetic Procedures

General Details

All reagents were purchased from commercial sources and used as received unless stated otherwise. Solvents used for all synthetic steps were named as follows. EA: ethyl acetate, DCM: dichloromethane, MeOH: methanol, DMF: dimethylformamide, THF: tetrahydrofuran, ACN: acetonitrile, H_2O: water. Analytical TLC has been performed on Silicycle SiliaPlate TLC plates and visualized under UV light (254 nm) or by staining with potassium permanganate. Flash chromatography was carried out on Sigma silica gel (200-300 mesh). Automated column chromatographic purification was carried out on a CombiFlash Rf system (Teledyne Isco). All new compounds were characterized by UPLC-SQD-LC-MS (Waters). (4.6 mm ×150 mm 5 µm C18 column; 10 µL injection; 10-100% CH_3CN/H_2O, linear-gradient, with constant 0.1% v/v TFA additive; 8 min run; ESI; positive ion mode; UV detection at 190-500 nm)

Synthetic procedure of Cyanopyrrolidine derivatives

Synthesis of CNP7

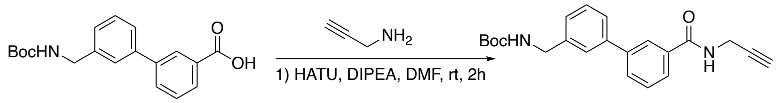

In a 5 mL round-bottom flask, 3′-(((*tert*-butoxycarbonyl)amino)methyl)-[1,1′-biphenyl]-3-carboxylic acid (50 mg, 0.15 mmol) was dissolved in 5 mL of DMF. DIPEA (58 mg, 0.45 mmol) and HATU (85 mg, 0.225 mmol) were added, and the reaction mixture was stirred at room temperature for 30 min. Propargylamine (9 mg, 0.165 mmol) was then added, and the mixture was stirred overnight. The reaction was quenched with water and extracted with ethyl acetate. The organic layer was dried over anhydrous sodium sulfate and concentrated under reduced pressure. The crude product was purified by silica gel column chromatography on a CombiFlash system, eluting with a gradient of 0–50% ethyl acetate in hexanes, to afford the product as a white solid (40 mg, 68%).

^{1}H NMR (500 MHz, $CDCl_3$) δ 7.97 (s, 1H), 7.74 (d, J = 7.7 Hz, 1H), 7.68 (d, J = 7.8 Hz, 1H), 7.52 – 7.42 (m, 3H), 7.39 (t, J = 7.6 Hz, 1H), 7.28 (d, J = 7.4 Hz, 1H), 6.72 (s, 1H), 5.04 (s, 1H), 4.36 (d, J = 6.1 Hz, 2H), 4.26 (dd, J = 5.2, 2.6 Hz, 2H), 2.28 (t, J = 2.6 Hz, 1H), 1.46 (s, 9H).

^{13}C NMR (126 MHz, $CDCl_3$) δ 167.16, 156.03, 141.45, 140.51, 139.67, 134.34, 130.42, 129.19, 129.06, 126.86, 126.21, 125.95, 125.91, 79.57, 77.31, 77.05, 76.80, 71.86, 60.42, 44.67, 29.82, 28.43, 14.20.

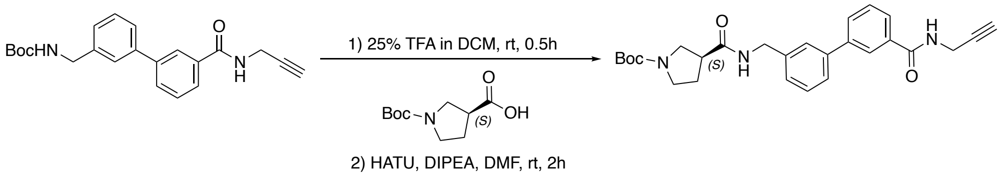

In a 5 mL round-bottom flask, *tert*-butyl ((3′-(prop-2-yn-1-ylcarbamoyl)-[1,1′-biphenyl]-3-yl)methyl)carbamate (30 mg, 0.11 mmol) was dissolved in 3 mL of DCM/TFA (3:1, v/v) and stirred at room temperature for 30 min. The solvents were removed under reduced pressure, and the crude product was used directly without further purification. The residue was dissolved in 3 mL of DMF, followed by the addition of DIPEA (42 mg, 0.45 mmol) and HATU (62 mg, 0.165 mmol). After stirring for 30 min at room temperature, (*S*)-1-(tert-butoxycarbonyl)pyrrolidine-3-carboxylic acid (26 mg, 0.12 mmol) was added, and the reaction mixture was stirred overnight. The reaction was quenched with water and extracted with ethyl acetate. The organic layer was washed with brine, dried over anhydrous sodium sulfate, and concentrated under reduced pressure. The crude product was purified by silica gel column chromatography on a CombiFlash system, eluting with a gradient of 0–100% ethyl acetate in hexanes, to afford the product as a white solid. (32 mg, 57%)

^{1}H NMR (500 MHz, $CDCl_3$) δ 7.87 (d, J = 13.9 Hz, 1H), 7.65 (d, J = 6.5 Hz, 1H), 7.53 (t, J = 7.2 Hz, 1H), 7.40 – 7.22 (m, 5H), 7.19 – 7.09 (m, 1H), 6.90 (t, J = 5.9 Hz, 1H), 4.34 (dd, J = 14.1, 5.8 Hz, 2H), 4.13 (dd, J = 5.4, 2.6 Hz, 2H), 3.46 (q, J = 10.0 Hz, 1H), 3.40 – 3.27 (m, 2H), 3.14 (dd, J = 29.4, 7.5 Hz, 1H), 2.83 (p, J = 8.2 Hz, 1H), 2.16 (t, J = 2.6 Hz, 1H), 2.10 – 2.01 (m, 1H), 1.39 – 1.35 (m, 1H), 1.33 (s, 9H).

^{13}C NMR (126 MHz, $CDCl_3$) δ 171.21, 154.41, 141.14, 140.53, 138.98, 134.37, 130.27, 129.22, 129.01, 126.25, 126.04, 79.73, 79.48, 77.33, 77.08, 76.82, 71.53, 60.41, 48.71, 44.67, 43.53, 29.69, 29.32, 28.54, 28.47, 21.05, 14.19.

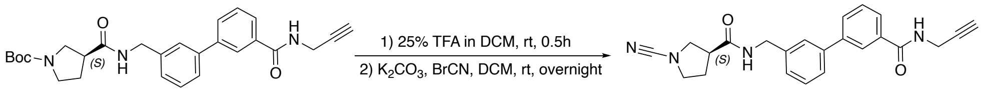

In a 5 mL round-bottom flask, *tert*-butyl (*S*)-3-(((3′-(prop-2-yn-1-ylcarbamoyl)-[1,1′-biphenyl]-3-yl)methyl)carbamoyl)pyrrolidine-1-carboxylate (30 mg, 0.065 mmol) was dissolved in 3 mL of DCM/TFA (3:1, v/v) and stirred at room temperature for 30 min. The solvents were removed under reduced pressure, and the residue was dissolved in 3 mL of DCM on ice. Potassium carbonate (36 mg, 0.26 mmol) and a 3 M solution of cyanogen bromide in DCM (26 µL, 0.08 mmol) were added, and the reaction mixture was stirred overnight at room temperature. The reaction was quenched with water (20 mL) and extracted with ethyl acetate (3 × 20 mL). The combined organic layers were dried over anhydrous sodium sulfate, filtered, and concentrated under reduced pressure. The crude product was purified by silica gel column chromatography using 0–100% ethyl acetate in hexanes as the eluent on a CombiFlash system. (16 mg, 53%)

^{1}H NMR (500 MHz, DMSO) δ 9.06 (t, J = 5.5 Hz, 1H), 8.62 (t, J = 5.9 Hz, 1H), 8.14 (s, 1H), 7.90 – 7.78 (m, 2H), 7.67 – 7.55 (m, 3H), 7.47 (t, J = 7.6 Hz, 1H), 7.29 (d, J = 7.6 Hz, 1H), 4.45 – 4.32 (m, 2H), 4.10 (dd, J = 5.5, 2.6 Hz, 2H), 3.56 (t, J = 8.6 Hz, 1H), 3.49 – 3.42 (m, 2H), 3.41 – 3.35 (m, 1H), 3.15 (t, J = 2.6 Hz, 1H), 3.07 (p, J = 7.5 Hz, 1H), 2.17 – 2.07 (m, 1H), 2.05 – 1.94 (m, 1H).

^{13}C NMR (126 MHz, DMSO) δ 171.88, 166.29, 140.65, 140.51, 140.00, 134.98, 130.05, 129.57 (2C), 127.16, 126.94, 126.29, 125.93(2C), 117.75, 81.75, 73.42, 52.96, 50.54, 43.70, 42.75, 30.02, 29.01.

HRMS *m/z* for $C_{23}H_{23}N_4O_2^+$ ($[M+H]^+$) calculated: 387.1821, found: 387.1825

Synthesis of CNP9

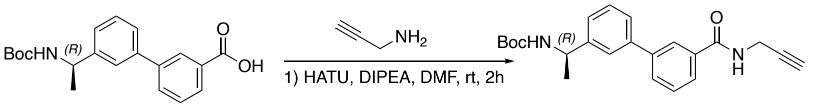

In a 5 mL round-bottom flask, (*R*)-3'-(1-((*tert*-butoxycarbonyl)amino)ethyl)-[1,1'-biphenyl]-3-carboxylic acid (30 mg, 0.09 mmol) was dissolved in 5 mL of DMF. DIPEA (35 mg, 0.27 mmol) and HATU (68 mg, 0.18 mmol) were added, and the reaction mixture was stirred at room temperature for 30 min. Propargylamine (6 mg, 0.11 mmol) was then added, and the mixture was stirred overnight. The reaction was quenched with water and extracted with ethyl acetate. The organic layer was dried over anhydrous sodium sulfate and concentrated under reduced pressure. The crude product was purified by silica gel column chromatography on a CombiFlash system, eluting with a gradient of 0–50% ethyl acetate in hexanes, to afford the product as a white solid (22 mg, 61%).

^{1}H NMR (500 MHz, $CDCl_3$) δ 7.98 (t, J = 2.0 Hz, 1H), 7.74 (d, J = 7.8 Hz, 1H), 7.71 (d, J = 7.8 Hz, 1H), 7.49 (dd, J = 16.0, 8.3 Hz, 3H), 7.42 (d, J = 7.6 Hz, 1H), 7.31 (d, J = 7.7 Hz, 1H), 6.53 (s, 1H), 4.88 (d, J = 27.2 Hz, 2H), 4.28 (dt, J = 4.9, 2.3 Hz, 2H), 2.29 (t, J = 2.6 Hz, 1H), 1.52 – 1.39 (m, 12H).

^{13}C NMR (126 MHz, $CDCl_3$) δ 167.11, 141.73, 140.50, 134.34, 130.54, 129.18, 129.07, 126.06, 125.94, 125.82, 125.25, 124.82, 79.51, 77.28, 77.23, 77.03, 76.78, 71.93, 30.93, 29.85, 28.40, 22.91.

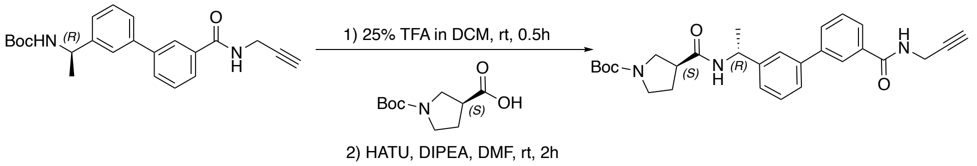

In a 5 mL round-bottom flask, *tert*-butyl (*R*)-(1-(3'-(prop-2-yn-1-ylcarbamoyl)-[1,1'-biphenyl]-3-yl)ethyl)carbamate (20 mg, 0.05 mmol) was dissolved in 3 mL of DCM/TFA (3:1, v/v) and stirred at room temperature for 30 min. The solvents were removed under reduced pressure, and the crude product was used directly without further purification. The residue was dissolved in 3 mL of DMF, followed by the addition of DIPEA (23 mg, 0.15 mmol) and HATU (38 mg, 0.1 mmol). After stirring for 30 min at room temperature, (*S*)-1-(tert-butoxycarbonyl)pyrrolidine-3-carboxylic acid (14 mg, 0.06 mmol) was added, and the reaction mixture was stirred overnight. The reaction was quenched with water and extracted with ethyl acetate. The organic layer was washed with brine, dried over anhydrous sodium sulfate, and concentrated under reduced pressure. The crude product was purified by silica gel column chromatography on a CombiFlash system, eluting with a gradient of 0–100% ethyl acetate in hexanes, to afford the product as a white solid. (18 mg, 53%)

^{1}H NMR (500 MHz, $CDCl_3$) δ 8.00 (s, 1H), 7.73 (dd, J = 24.5, 7.4 Hz, 2H), 7.55 – 7.47 (m, 3H), 7.43 (t, J = 7.7 Hz, 1H), 7.31 (d, J = 7.7 Hz, 1H), 6.63 (d, J = 28.2 Hz, 1H), 5.94 (d, J = 7.2 Hz, 1H), 5.18 (d, J = 6.3 Hz, 1H), 4.32 – 4.24 (m, 2H), 3.69 – 3.44 (m, 3H), 3.31 (q, J = 8.3 Hz, 1H), 2.84 (s, 1H), 2.29 (t, J = 2.6 Hz, 1H), 2.06 (d, J = 4.7 Hz, 1H), 1.62 (s, 5H), 1.44 (s, 9H).

^{13}C NMR (126 MHz, $CDCl_3$) δ 167.07, 141.53, 140.76, 134.41, 130.50, 129.37, 129.13, 126.42, 125.52, 124.98, 79.54, 79.51, 77.28, 77.23, 77.02, 76.77, 71.90, 48.67, 29.85, 28.49, 22.05.

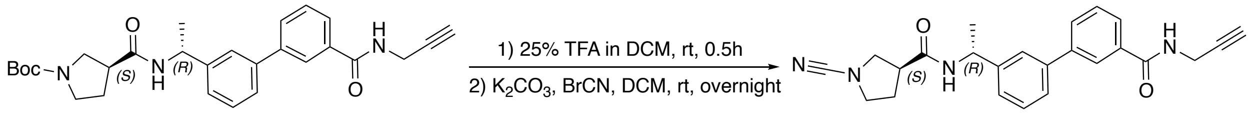

In a 5 mL round-bottom flask, *tert*-butyl (*S*)-3-(((*R*)-1-(3'-(prop-2-yn-1-ylcarbamoyl)-[1,1'-biphenyl]-3-yl)ethyl)carbamoyl)pyrrolidine-1-carboxylate (18 mg, 0.038 mmol) was dissolved in 3 mL of DCM/TFA (3:1, v/v) and stirred at room temperature for 30 min. The solvents were removed under reduced pressure, and the residue was dissolved in 3 mL of DCM on ice. Potassium carbonate (22 mg, 0.26 mmol) and a 3 M solution of cyanogen bromide in DCM (15 μL, 0.08 mmol) were added, and the reaction mixture was stirred overnight at room temperature. The reaction was quenched with water (20 mL) and extracted with ethyl acetate (3 × 20 mL). The combined organic layers were dried over anhydrous sodium sulfate, filtered, and concentrated under reduced pressure. The crude product was purified by silica gel column chromatography using 0–100% ethyl acetate in hexanes as the eluent on a CombiFlash system. (5 mg, 28%)

^{1}H NMR (600 MHz, DMSO) δ 9.07 (t, J = 5.6 Hz, 1H), 8.57 (d, J = 7.9 Hz, 1H), 8.12 (s, 1H), 7.84 (dd, J = 16.1, 8.0 Hz, 2H), 7.65 (s, 1H), 7.59 (q, J = 7.7 Hz, 2H), 7.46 (t, J = 7.7 Hz, 1H), 7.34 (d, J = 7.7 Hz, 1H), 5.01 (p, J = 7.1 Hz, 1H), 4.10 (dd, J = 5.6, 2.5 Hz, 2H), 3.58 – 3.50 (m, 1H), 3.46 – 3.36 (m, 3H), 3.16 (t, J = 2.5 Hz, 1H), 3.06 (p, J = 7.5 Hz, 1H), 2.12 – 2.04 (m, 1H), 1.94 – 1.85 (m, 1H), 1.41 (d, J = 7.1 Hz, 3H).

^{13}C NMR (151 MHz, DMSO) δ 170.96, 166.32, 145.95, 140.80, 140.02, 134.97, 130.12, 129.51(2C), 126.89, 125.98, 125.74(2C), 124.87, 117.77, 81.75, 73.43, 52.87, 50.52, 48.60, 43.59, 30.05, 29.01, 23.16.

HRMS *m/z* for $C_{24}H_{25}N_4O_2^+$ ($[M+H]^+$) calculated: 401.1978, found: 401.1980

Synthesis of other Cyanopyrrolidine Derivatives.

The synthesis of all the other Cyanopyrrolidine derivatives was performed as described earlier.

CNP1

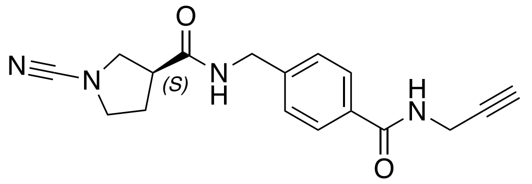

^{1}H NMR (500 MHz, DMSO) δ 8.89 (t, J = 5.6 Hz, 1H), 8.61 (t, J = 5.9 Hz, 1H), 7.82 (d, J = 8.4 Hz, 2H), 7.33 (d, J = 8.2 Hz, 2H), 4.34 (d, J = 4.6 Hz, 2H), 4.05 (dt, J = 3.9, 2.0 Hz, 2H), 3.56 (dd, J = 9.3, 7.8 Hz, 1H), 3.47 – 3.41 (m, 2H), 3.41 – 3.36 (m, 1H), 3.12 (t, J = 2.5 Hz, 1H), 3.06 (p, J = 7.4 Hz, 1H), 2.14 – 2.06 (m, 1H), 2.02 – 1.94 (m, 1H).

^{13}C NMR (126 MHz, DMSO) δ 171.94, 166.16, 143.34, 132.88, 127.85 (2C), 127.46 (2C), 117.74, 81.84, 73.29, 52.95, 50.52, 43.63, 42.26, 29.94, 28.93.

HRMS *m/z* for $C_{17}H_{19}N_4O_2^+$ ($[M+H]^+$) calculated: 311.1503, found: 311.1508

CNP2

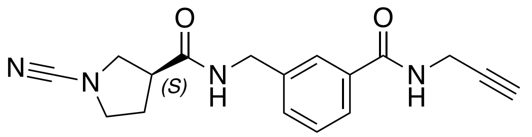

^{1}H NMR (500 MHz, DMSO) δ 8.93 (t, J = 5.6 Hz, 1H), 8.61 (t, J = 6.0 Hz, 1H), 7.79 – 7.69 (m, 2H), 7.43 (d, J = 7.0 Hz, 2H), 4.37 – 4.30 (m, 2H), 4.05 (dd, J = 5.6, 2.5 Hz, 2H), 3.60 – 3.51 (m, 1H), 3.47 – 3.35 (m, 3H), 3.12 (t, J = 2.5 Hz, 1H), 3.06 (p, J = 7.5 Hz, 1H), 2.10 (td, J = 12.5, 7.3 Hz, 1H), 2.04 – 1.92 (m, 1H).

^{13}C NMR (126 MHz, DMSO) δ 171.89, 166.34, 139.99, 134.41, 130.70, 128.86, 126.84, 126.10, 117.74, 81.80, 73.29, 52.92, 50.52, 43.67, 42.54, 29.99, 28.96.

HRMS *m/z* for $C_{17}H_{19}N_4O_2^+$ ($[M+H]^+$) calculated: 311.1503, found: 311.1508

CNP3

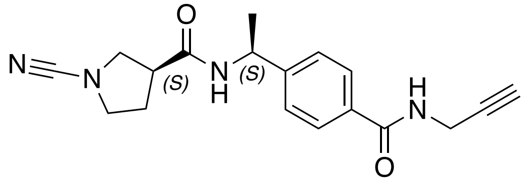

^{1}H NMR (600 MHz, DMSO) δ 8.87 (t, J = 5.6 Hz, 1H), 8.54 (d, J = 7.8 Hz, 1H), 7.81 (d, J = 8.1 Hz, 2H), 7.38 (d, J = 8.1 Hz, 2H), 4.94 (p, J = 7.2 Hz, 1H), 4.05 (dd, J = 5.6, 2.5 Hz, 2H), 3.53 (dd, J = 9.3, 7.8 Hz, 1H), 3.43 – 3.35 (m, 3H), 3.12 (t, J = 2.5 Hz, 1H), 3.04 (p, J = 7.3 Hz, 1H), 2.09 – 2.03 (m, 1H), 1.92 – 1.83 (m, 1H), 1.36 (d, J = 7.0 Hz, 3H).

^{13}C NMR (151 MHz, DMSO) δ 171.03, 166.19, 148.61, 132.76, 127.85 (2C), 126.20 (2C), 117.75, 81.84, 73.28, 52.84, 50.48, 48.32, 43.53, 29.99, 28.91, 22.72.

HRMS *m/z* for $C_{18}H_{21}N_4O_2^+$ ($[M+H]^+$) calculated: 325.1659, found: 325.1665

CNP4

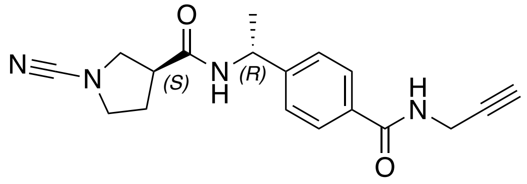

^{1}H NMR (600 MHz, DMSO) δ 8.87 (t, J = 5.6 Hz, 1H), 8.54 (d, J = 7.8 Hz, 1H), 7.81 (d, J = 8.1 Hz, 2H), 7.38 (d, J = 8.1 Hz, 2H), 4.94 (p, J = 7.2 Hz, 1H), 4.05 (dd, J = 5.6, 2.5 Hz, 2H), 3.53 (dd, J = 9.3, 7.8 Hz, 1H), 3.44 – 3.34 (m, 3H), 3.12 (t, J = 2.5 Hz, 1H), 3.04 (p, J = 7.3 Hz, 1H), 2.06 (ddd, J = 12.8, 5.4, 2.3 Hz, 1H), 1.87 (dq, J = 12.4, 7.5 Hz, 1H), 1.36 (d, J = 7.0 Hz, 3H).

^{13}C NMR (151 MHz, DMSO) δ 171.03, 166.19, 148.61, 132.76, 127.85 (2C), 126.20 (2C), 117.75, 81.84, 73.28, 52.84, 50.48, 48.32, 43.53, 29.99, 28.91, 22.72.

HRMS *m/z* for $C_{18}H_{21}N_4O_2^+$ ($[M+H]^+$) calculated: 325.1659, found: 325.1665

CNP5

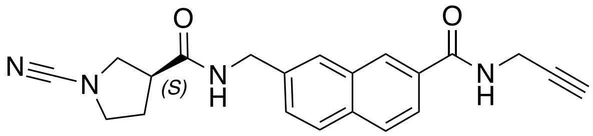

1H NMR (600 MHz, DMSO) δ 9.14 (t, J = 5.5 Hz, 1H), 8.65 (t, J = 5.6 Hz, 1H), 8.59 (d, J = 1.7 Hz, 1H), 8.04 (d, J = 8.5 Hz, 1H), 7.97 (dd, J = 8.5, 1.6 Hz, 1H), 7.91 (d, J = 8.2 Hz, 1H), 7.59 (dd, J = 8.2, 7.0 Hz, 1H), 7.49 (d, J = 7.0 Hz, 1H), 4.92 – 4.77 (m, 2H), 4.15 (dd, J = 5.5, 2.5 Hz, 2H), 3.56 (dd, J = 9.3, 7.8 Hz, 1H), 3.52 – 3.44 (m, 2H), 3.38 (dt, J = 8.9, 7.3 Hz, 1H), 3.17 (t, J = 2.5 Hz, 1H), 3.11 (p, J = 7.3 Hz, 1H), 2.11 (dtd, J = 12.7, 7.4, 5.4 Hz, 1H), 2.03 (dq, J = 12.4, 7.5 Hz, 1H).

^{13}C NMR (151 MHz, DMSO) δ 171.99, 166.51, 136.19, 135.04, 131.65, 130.44, 129.18, 127.74, 127.71, 125.95, 124.58, 123.62, 117.78, 81.76, 73.50, 52.97, 50.53, 43.64, 40.66, 30.08, 29.08.

HRMS *m/z* for $C_{21}H_{21}N_4O_2^+$ ([M+H]$^+$) calculated: 361.1665, found: 361.1667

CNP6

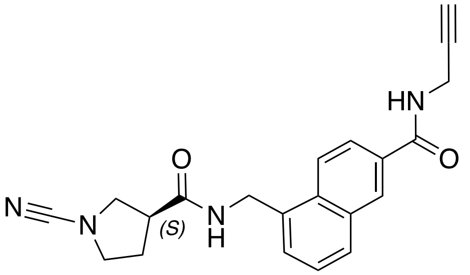

^{1}H NMR (500 MHz, DMSO) δ 9.08 (t, J = 5.7 Hz, 1H), 8.69 (t, J = 6.1 Hz, 1H), 8.45 (s, 1H), 8.05 – 7.89 (m, 3H), 7.80 (s, 1H), 7.49 (d, J = 8.4 Hz, 1H), 4.48 (d, J = 6.1 Hz, 2H), 4.17 – 4.05 (m, 2H), 3.57 (d, J = 9.2 Hz, 1H), 3.52 – 3.36 (m, 3H), 3.20 – 3.03 (m, 2H), 2.18 – 2.07 (m, 1H), 2.07 – 1.96 (m, 1H).

^{13}C NMR (126 MHz, DMSO) δ 171.98, 166.43, 139.33, 134.63, 131.68, 131.37, 129.60, 128.19, 127.96, 127.09, 125.57, 124.81, 117.76, 81.81, 73.37, 53.01, 50.55, 43.73, 42.83, 29.97, 29.07.

HRMS *m/z* for $C_{21}H_{21}N_4O_2^+$ ([M+H]$^+$) calculated: 361.1665, found: 361.1663

CNP8

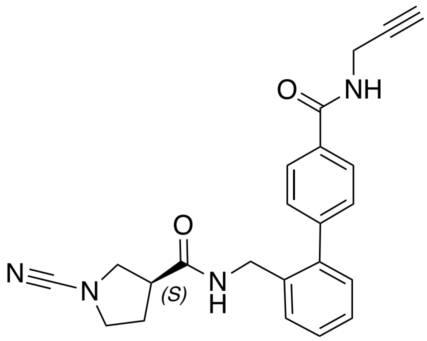

^{1}H NMR (500 MHz, DMSO) δ 8.99 (t, J = 5.6 Hz, 1H), 8.44 (t, J = 5.5 Hz, 1H), 7.97 – 7.87 (m, 2H), 7.47 (d, J = 8.2 Hz, 2H), 7.45 – 7.34 (m, 3H), 7.29 – 7.21 (m, 1H), 4.27 – 4.16 (m, 2H), 4.09 (dt, J = 4.0, 2.0 Hz, 2H), 3.49 (dd, J = 9.3, 7.8 Hz, 1H), 3.43 – 3.33 (m, 3H), 3.13 (t, J = 2.5 Hz, 1H), 2.99 (p, J = 7.3 Hz, 1H), 2.03 (dtd, J = 12.7, 7.3, 5.4 Hz, 1H), 1.89 (dq, J = 12.5, 7.5 Hz, 1H).
^{13}C NMR (126 MHz, DMSO) δ 171.62, 166.09, 143.87, 140.51, 136.48, 132.99, 130.12, 129.48 (2C), 128.63, 128.45, 127.72 (2C), 127.62, 117.72, 81.81, 73.31, 52.88, 50.48, 43.50, 43.46, 29.91, 29.00.

HRMS *m/z* for $C_{23}H_{23}N_4O_2^+$ ([M+H]$^+$) calculated: 387.1821, found: 387.1824

CNP10

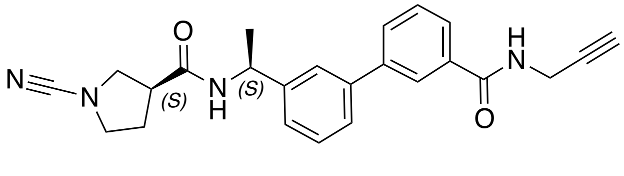

^{1}H NMR (600 MHz, DMSO) δ 9.07 (t, J = 5.6 Hz, 1H), 8.55 (d, J = 8.0 Hz, 1H), 8.13 (t, J = 1.8 Hz, 1H), 7.85 (ddt, J = 14.0, 7.7, 1.3 Hz, 2H), 7.66 (s, 1H), 7.59 (q, 2H), 7.46 (t, J = 7.6 Hz, 1H), 7.34 (d, J = 7.6 Hz, 1H), 5.01 (p, 1H), 4.10 (dd, J = 5.5, 2.5 Hz, 2H), 3.53 (dd, J = 9.3, 7.8 Hz, 1H),

3.43 (td, J = 8.4, 5.4 Hz, 1H), 3.40 – 3.33 (m, 2H), 3.16 (t, J = 2.5 Hz, 1H), 3.06 (q, J = 7.3 Hz, 1H), 2.14 – 2.05 (m, 1H), 2.03 – 1.94 (m, 1H), 1.42 (d, J = 7.0 Hz, 3H).

^{13}C NMR (151 MHz, DMSO) δ 170.97, 166.32, 145.81, 140.80, 140.03, 134.96, 130.16, 129.56, 129.52, 126.91, 125.98, 125.85, 125.78, 125.04, 117.74, 81.76, 73.43, 52.92, 50.53, 48.66, 43.63, 29.99, 29.01, 23.13.

HRMS *m/z* for $C_{24}H_{25}N_4O_2^+$ ($[M+H]^+$) calculated: 401.1978, found: 401.1982

CNP11

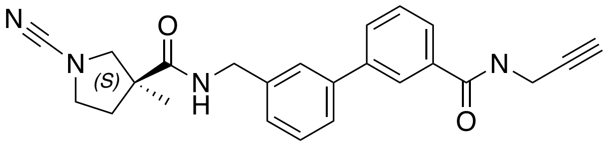

^{1}H NMR (500 MHz, DMSO) δ 9.06 (t, J = 5.5 Hz, 1H), 8.49 (t, J = 5.9 Hz, 1H), 8.13 (s, 1H), 7.83 (dd, J = 21.4, 7.8 Hz, 2H), 7.66 – 7.54 (m, 3H), 7.46 (t, J = 7.7 Hz, 1H), 7.27 (d, J = 7.6 Hz, 1H), 4.40 (d, J = 6.0 Hz, 2H), 4.10 (dd, J = 5.5, 2.5 Hz, 2H), 3.77 (d, J = 9.4 Hz, 1H), 3.52 – 3.45 (m, 1H), 3.41 – 3.34 (m, 1H), 3.20 – 3.13 (m, 2H), 2.35 – 2.25 (m, 1H), 1.87 – 1.79 (m, 1H), 1.32 (s, 3H).

^{13}C NMR (126 MHz, DMSO) δ 174.21, 166.29, 140.91, 140.68, 139.92, 135.00, 129.99, 129.60, 129.53, 126.91, 126.79, 125.96, 125.93, 125.76, 117.79, 81.74, 73.42, 58.83, 49.79, 49.71, 42.80, 35.75, 29.01, 22.48.

HRMS *m/z* for $C_{24}H_{25}N_4O_2^+$ ($[M+H]^+$) calculated: 401.1978, found: 401.1979

CNP12

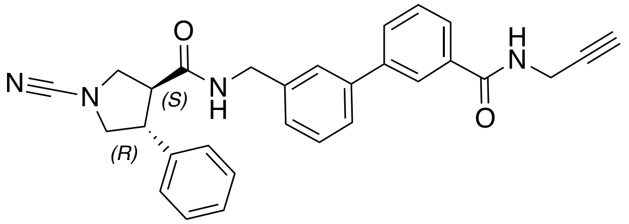

^1H NMR (500 MHz, DMSO) δ 9.06 (t, J = 5.5 Hz, 1H), 8.61 (t, J = 5.8 Hz, 1H), 8.09 (t, J = 1.8 Hz, 1H), 7.86 (dt, J = 7.8, 1.4 Hz, 1H), 7.73 (dt, J = 7.9, 1.4 Hz, 1H), 7.61 – 7.51 (m, 2H), 7.44 (t, J = 1.9 Hz, 1H), 7.35 – 7.29 (m, 3H), 7.28 – 7.24 (m, 2H), 7.22 – 7.18 (m, 1H), 6.97 – 6.93 (m, 1H), 4.43 (dd, J = 15.3, 6.4 Hz, 1H), 4.19 (dd, J = 15.3, 5.2 Hz, 1H), 4.11 (dd, J = 5.5, 2.5 Hz, 2H), 3.89 – 3.79 (m, 2H), 3.63 (td, J = 10.1, 7.7 Hz, 1H), 3.53 (t, J = 9.1 Hz, 1H), 3.47 (dd, J = 10.1, 9.0 Hz, 1H), 3.23 (dt, J = 10.1, 8.7 Hz, 1H), 3.15 (t, J = 2.5 Hz, 1H).

^{13}C NMR (126 MHz, DMSO) δ 170.35, 166.29, 140.67, 140.17, 139.97, 138.65, 134.94, 130.06, 129.53, 129.38, 128.97 (2C), 128.10 (2C), 127.65, 126.89, 126.81, 126.09, 125.98, 125.83, 117.36, 81.76, 73.41, 56.54, 53.89, 50.97, 48.46, 42.65, 29.02.

HRMS *m/z* for $C_{29}H_{27}N_4O_2^+$ ($[M+H]^+$) calculated: 463.2134, found:463.2127

CNP13

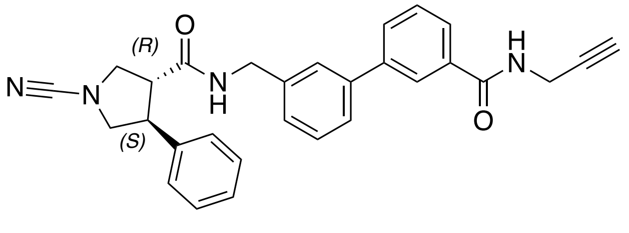

^1H NMR (600 MHz, DMSO) δ 9.07 (t, J = 5.6 Hz, 1H), 8.62 (t, J = 6.0 Hz, 1H), 8.08 (s, 1H), 7.86 (d, J = 7.8 Hz, 1H), 7.73 (d, J = 7.9 Hz, 1H), 7.63 – 7.52 (m, 2H), 7.44 (s, 1H), 7.32 (dd, J = 15.0, 7.3 Hz, 3H), 7.26 (t, J = 7.5 Hz, 2H), 7.20 (t, J = 7.2 Hz, 1H), 6.94 (d, J = 7.7 Hz, 1H), 4.43 (dd, J

= 15.3, 6.5 Hz, 1H), 4.19 (dd, J = 15.3, 5.2 Hz, 1H), 4.11 (dd, J = 5.6, 2.5 Hz, 2H), 3.84 (dt, J = 17.3, 9.1 Hz, 2H), 3.68 – 3.59 (m, 1H), 3.53 (t, J = 9.1 Hz, 1H), 3.51 – 3.45 (m, 1H), 3.23 (q, J = 8.7 Hz, 1H), 3.16 (t, J = 2.5 Hz, 1H).

^{13}C NMR (126 MHz, DMSO) δ 170.34, 166.29, 140.66, 140.16, 139.96, 138.62, 134.92, 130.07, 129.54, 129.38, 128.97 (2C), 128.11 (2C), 127.66, 126.89, 126.80, 126.08, 125.98, 125.83, 117.37, 81.75, 73.43, 56.52, 53.87, 50.96, 48.46, 42.64, 29.01.

HRMS *m/z* for $C_{29}H_{27}N_4O_2^+$ ($[M+H]^+$) calculated: 463.2134, found:463.2127

Synthesis of CNP7-Biotin.

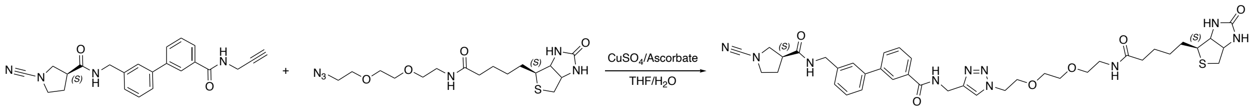

Sodium ascorbate (16.1 mg, 0.081 mmol, 1.5 equiv.) and $CuSO_4.5H_2O$ (26.9 mg, 0.108 mmol, 2 equiv.) were completely dissolved in water (1 mL, 0.07 M). The mixture was then added to a solution of (*S*)-1-cyano-N-((3'-(prop-2-yn-1-ylcarbamoyl)-[1,1'-biphenyl]-3-yl)methyl)pyrrolidine-3-carboxamide (10.0 mg, 0.026 mmol, 1 equiv.) and N-(2-(2-(2-azidoethoxy)ethoxy)ethyl)-5-((4*S*)-2-oxohexahydro-1H-thieno[3,4-d]imidazol-4-yl)pentanamide (10.3 mg, 0.026 mmol, 1 equiv.) in THF (1 mL). The reaction was allowed to proceed at room temperature overnight. The reaction mixture was then dissolved in the brine, and extraction was performed with 20% Methanol in DCM. The combined organic layers were dried over sodium sulfate, then concentrated under reduced pressure. The residue was purified by column chromatography (0-30% Methanol in DCM) to provide the final product as a white solid. (3.8 mg, 19%)

CNP7-Biotin

^{1}H NMR (500 MHz, MeOD) δ 7.44 (t, J = 1.8 Hz, 1H), 7.32 (s, 1H), 7.20 – 7.09 (m, 2H), 6.95 – 6.84 (m, 3H), 6.77 (t, J = 7.7 Hz, 1H), 6.63 (d, J = 7.6 Hz, 1H), 4.00 (s, 2H), 3.90 (t, J = 5.0 Hz, 2H), 3.77 (d, J = 8.9 Hz, 3H), 3.58 (dd, J = 7.9, 4.4 Hz, 1H), 3.21 (t, J = 5.0 Hz, 2H), 2.98 – 2.83 (m, 7H), 2.81 – 2.72 (m, 3H), 2.59 (t, J = 5.6 Hz, 2H), 2.50 – 2.40 (m, 2H), 2.24 – 2.17 (m, 1H), 2.00 (d, J = 12.7 Hz, 1H), 1.56 – 1.42 (m, 7H), 1.06 – 0.81 (m, 4H), 0.70 (p, J = 7.5 Hz, 2H), 0.61 (s, 1H).

^{13}C NMR (151 MHz, DMSO) δ 172.60, 170.96, 166.50, 163.16, 145.93, 140.74, 140.06, 135.28, 129.96, 129.50, 126.91, 125.98, 125.72, 125.69, 124.89, 123.86, 117.77, 69.93, 69.86, 69.60, 69.20, 61.49, 59.64, 55.88, 52.88, 50.51, 49.74, 48.62, 43.59, 40.52, 38.87, 35.55, 35.36, 30.04, 28.65, 28.50, 25.72, 23.16.

HRMS *m/z* for $C_{39}H_{51}N_{10}O_6S^+$ ($[M+H]^+$) calculated: 787.3714, found: 787.3711

CNP9-Biotin

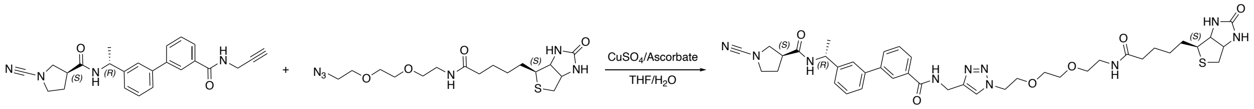

CNP9-Biotin was synthesized using the same synthetic procedure as for CNP7-Biotin, with CNP9 substituted for CNP7.

^{1}H NMR (600 MHz, DMSO) δ 9.19 (s, 1H), 8.57 (d, J = 7.9 Hz, 1H), 8.15 (d, J = 2.1 Hz, 1H), 7.97 (s, 1H), 7.88 (d, J = 7.4 Hz, 1H), 7.83 – 7.78 (m, 2H), 7.64 (d, J = 1.9 Hz, 1H), 7.58 (q, J = 8.0 Hz, 2H), 7.45 (t, J = 7.7 Hz, 1H), 7.33 (d, J = 7.7 Hz, 1H), 6.39 (d, J = 34.9 Hz, 2H), 5.00 (t, J = 7.3 Hz, 1H), 4.55 (d, J = 5.7 Hz, 2H), 4.50 (t, J = 5.3 Hz, 2H), 4.33 – 4.25 (m, 1H), 4.12 (d, J = 2.6 Hz, 1H), 3.80 (t, J = 5.3 Hz, 2H), 3.70 (s, 1H), 3.63 – 3.59 (m, 1H), 3.56 – 3.50 (m, 4H), 3.47 – 3.36 (m, 5H), 3.15 (q, J = 5.7 Hz, 2H), 3.07 (q, J = 7.4 Hz, 2H), 2.83 – 2.78 (m, 1H), 2.57 (d, J

= 12.4 Hz, 1H), 2.10 – 2.03 (m, 3H), 1.90 (dd, J = 12.3, 4.8 Hz, 1H), 1.59 (q, J = 9.0 Hz, 1H), 1.49 (dd, J = 14.2, 6.4 Hz, 2H), 1.41 (d, J = 7.0 Hz, 3H), 1.31 – 1.22 (m, 3H).

^{13}C NMR (151 MHz, DMSO) δ 172.60, 170.96, 166.50, 163.16, 145.93, 140.74, 140.06, 135.28, 129.96, 129.50, 126.91, 125.98, 125.72, 125.69, 124.89, 123.86, 117.77, 69.93, 69.86, 69.60, 69.20, 61.49, 59.64, 55.88, 52.88, 50.51, 49.74, 48.62, 43.59, 40.52, 38.87, 35.55, 35.36, 30.04, 28.65, 28.50, 25.72, 23.16, 14.56.

HRMS *m/z* for $C_{40}H_{53}N_{10}O_{6}S^{+}$ ([M+H]$^{+}$) calculated: 801.3870, found: 801.3869

CNP1

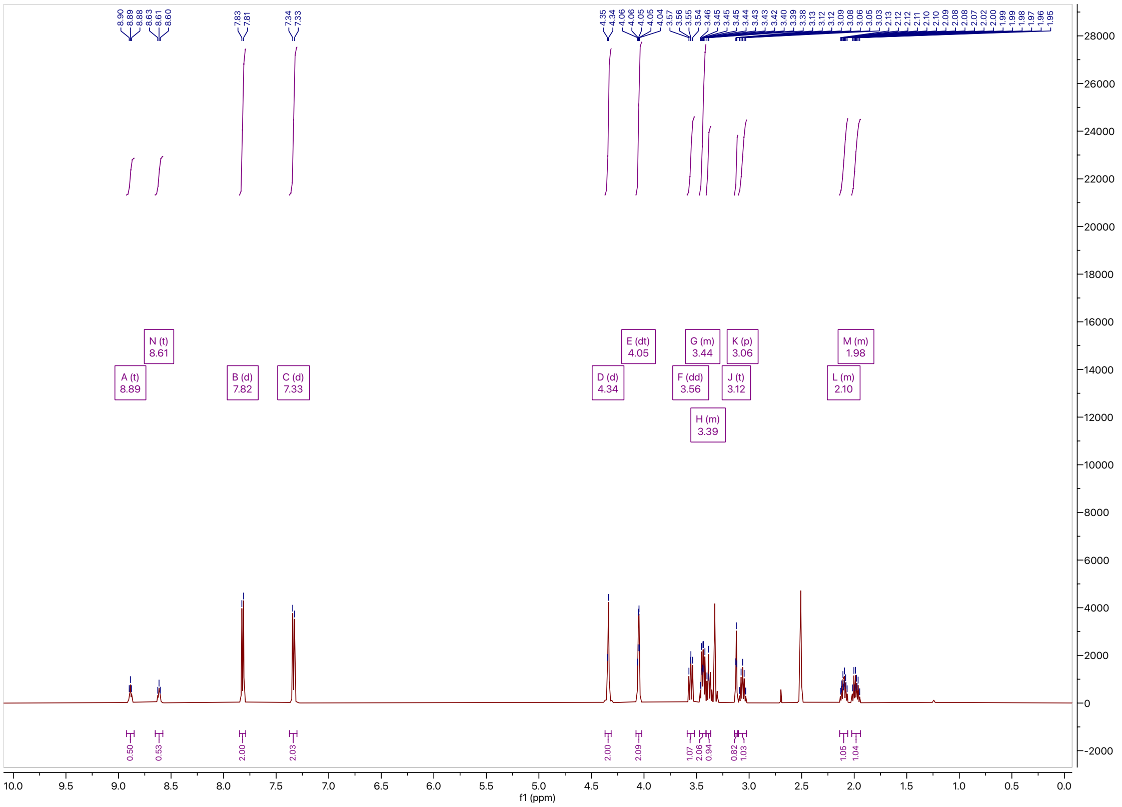

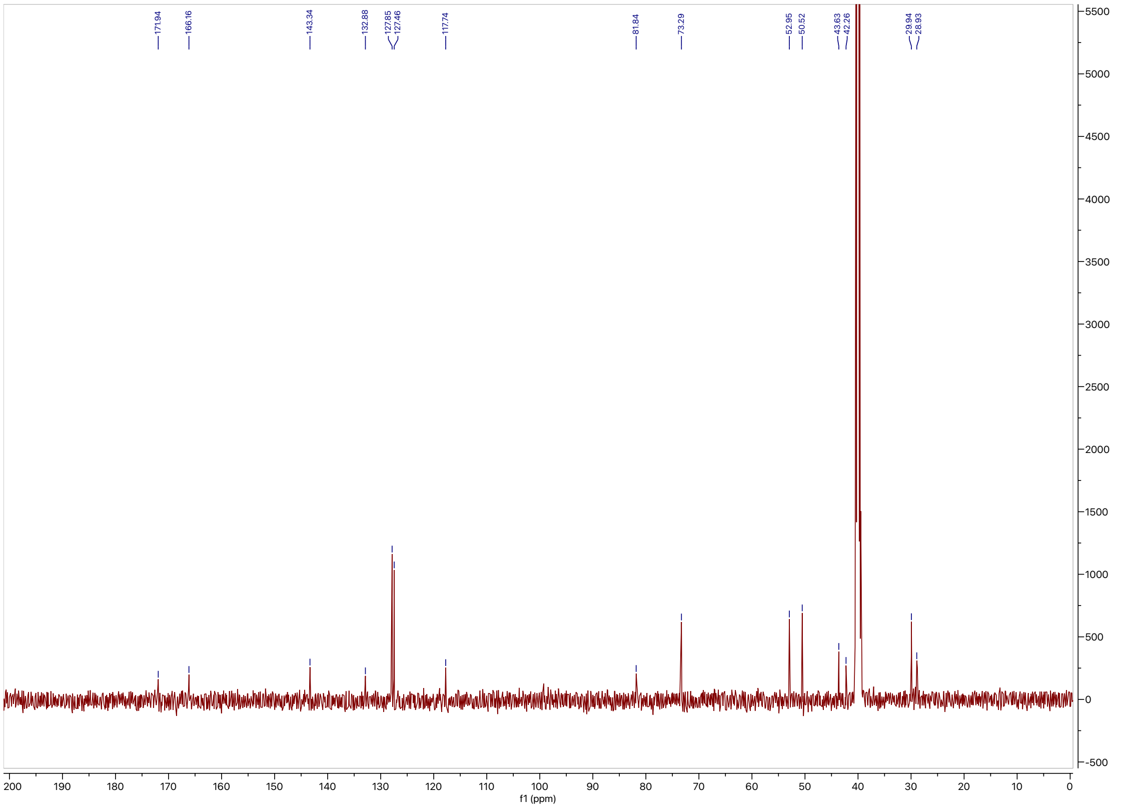

CNP2

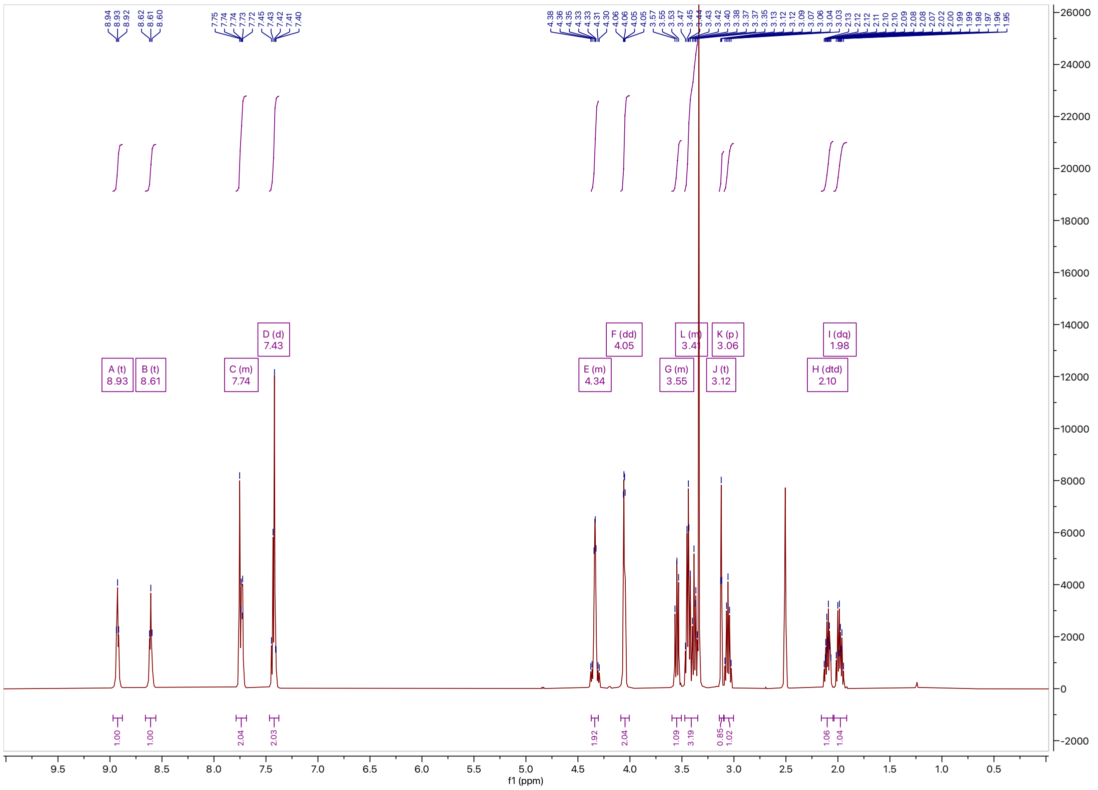
A (t) 8.93
B (t) 8.61
C (m) 7.74
D (d) 7.43
E (m) 4.34
F (dd) 4.05
G (m) 3.55
L (m) 3.41
J (t) 3.12
K (p) 3.06
H (dtd) 2.10
I (dq) 1.98
f1 (ppm)

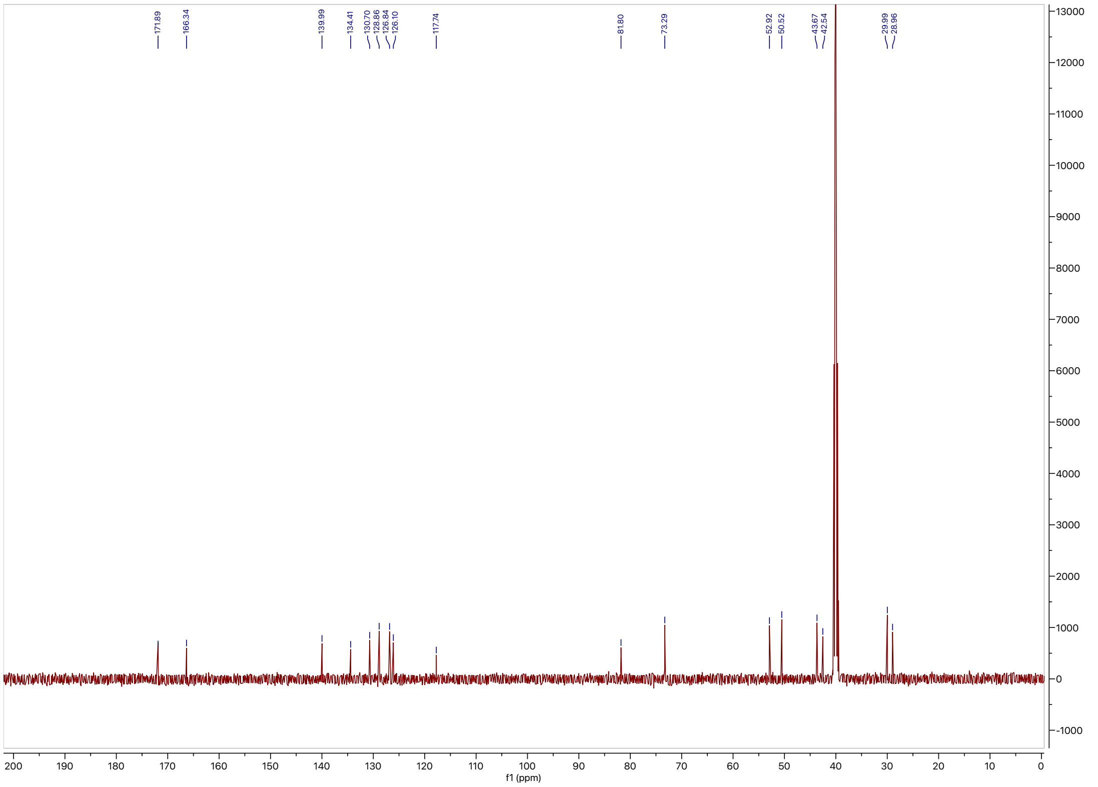
171.89
166.34
139.99
134.41
130.70
128.86
126.84
126.10
117.74
81.80
73.29
52.92
50.52
43.67
42.54
29.99
28.96
f1 (ppm)

CNP3

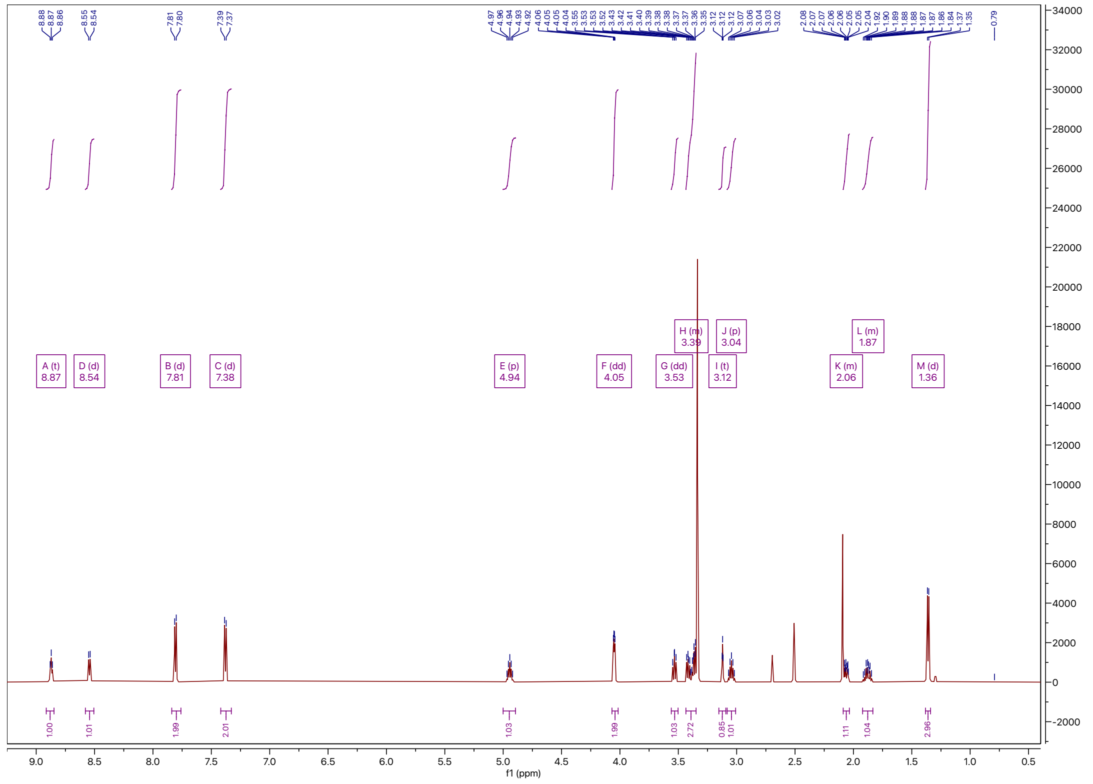

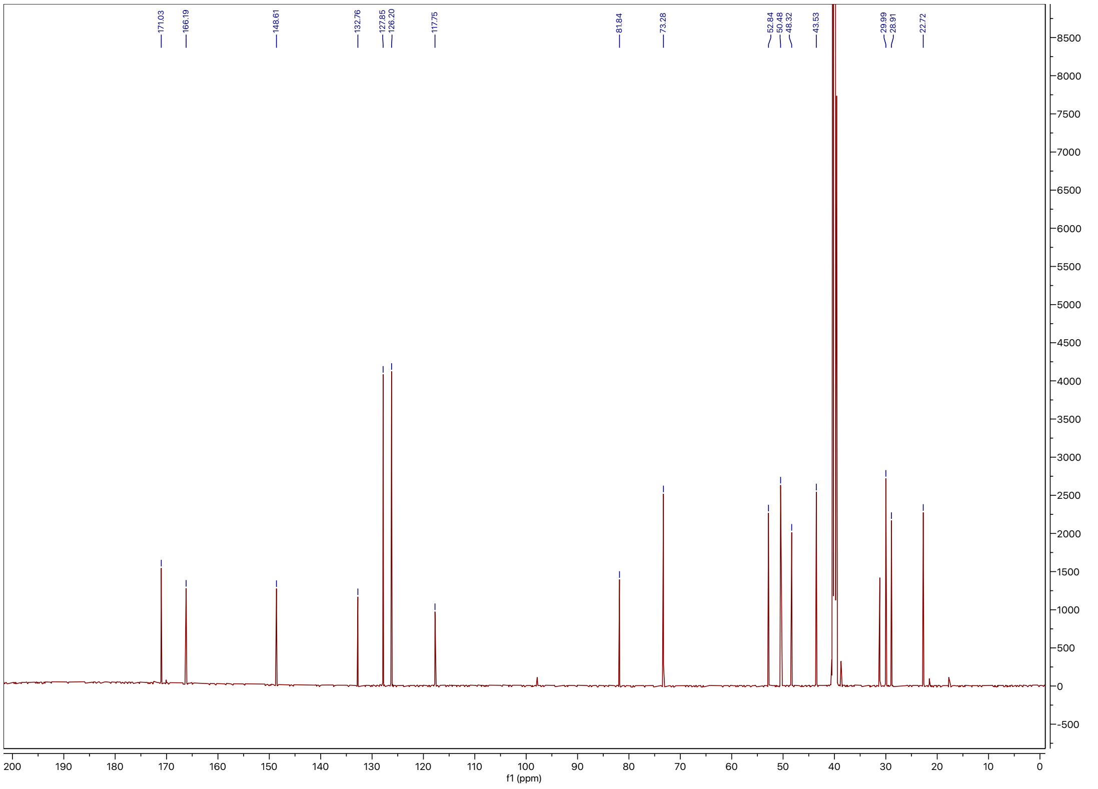

CNP4

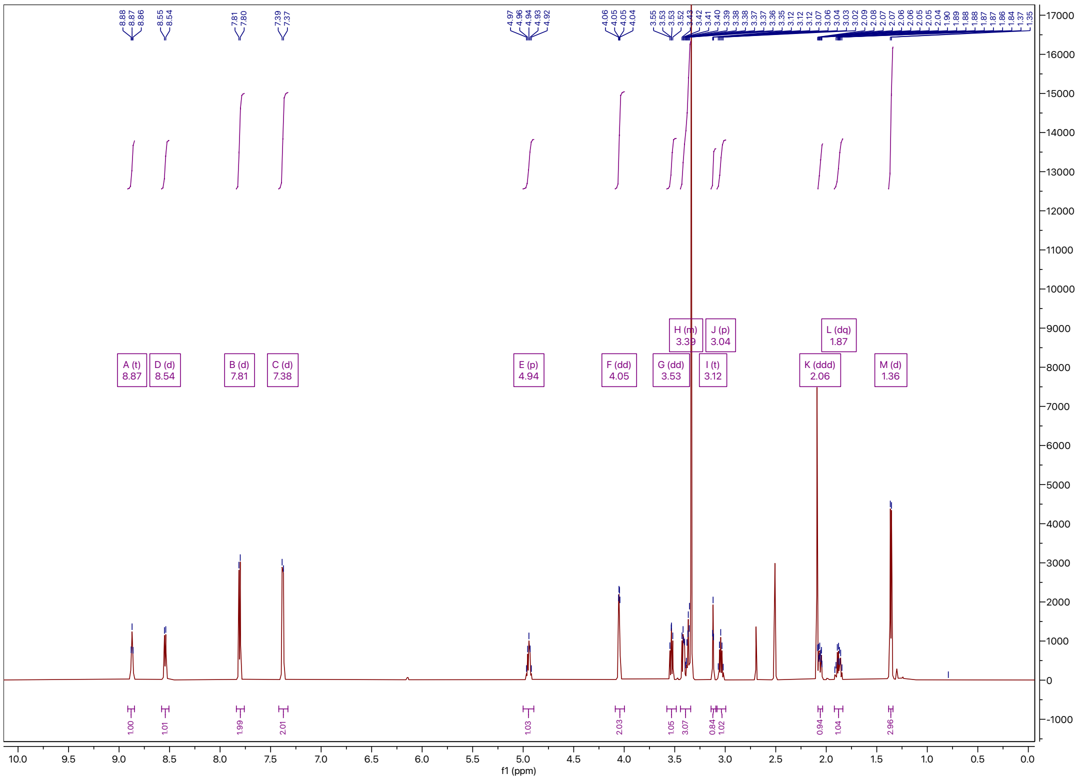
A (t)
8.87
D (d)
8.54
B (d)
7.81
C (d)
7.38
E (p)
4.94
F (dd)
4.05
G (dd)
3.53
H (m)
3.39
I (t)
3.12
J (p)
3.04
K (ddd)
2.06
L (dq)
1.87
M (d)
1.36
f1 (ppm)

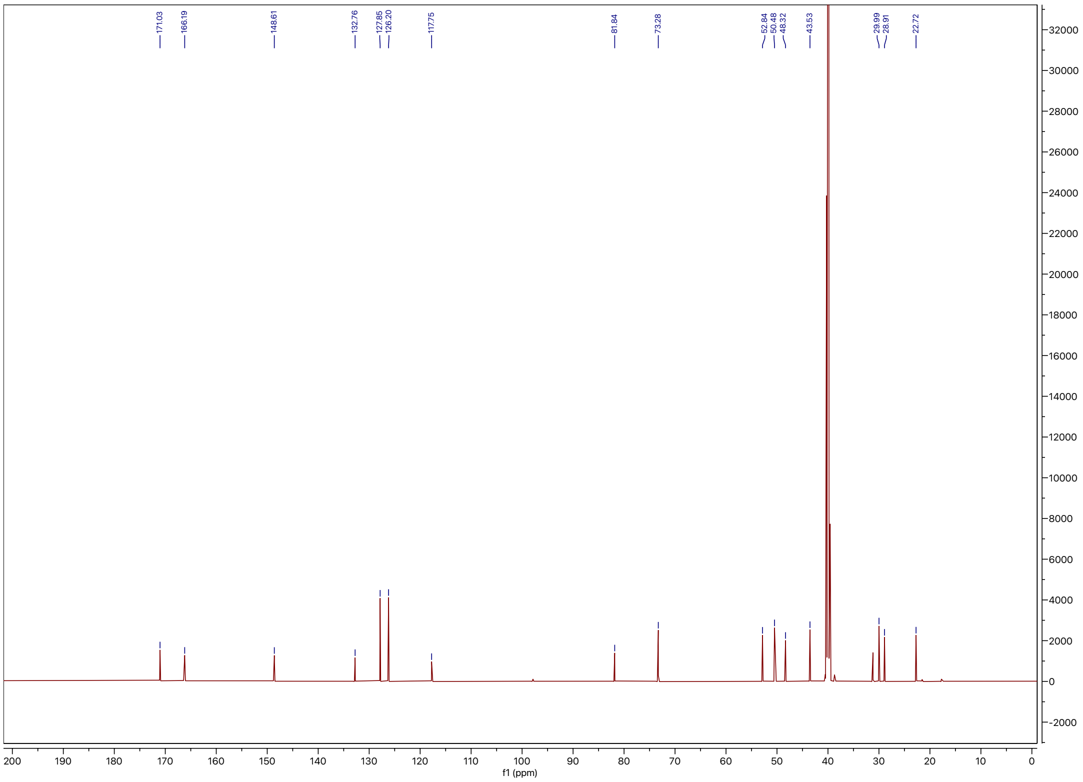
171.03
166.19
148.61
132.76
127.85
126.20
117.75
81.84
73.28
52.84
50.48
48.32
43.53
29.99
28.91
22.72
f1 (ppm)

CNP5

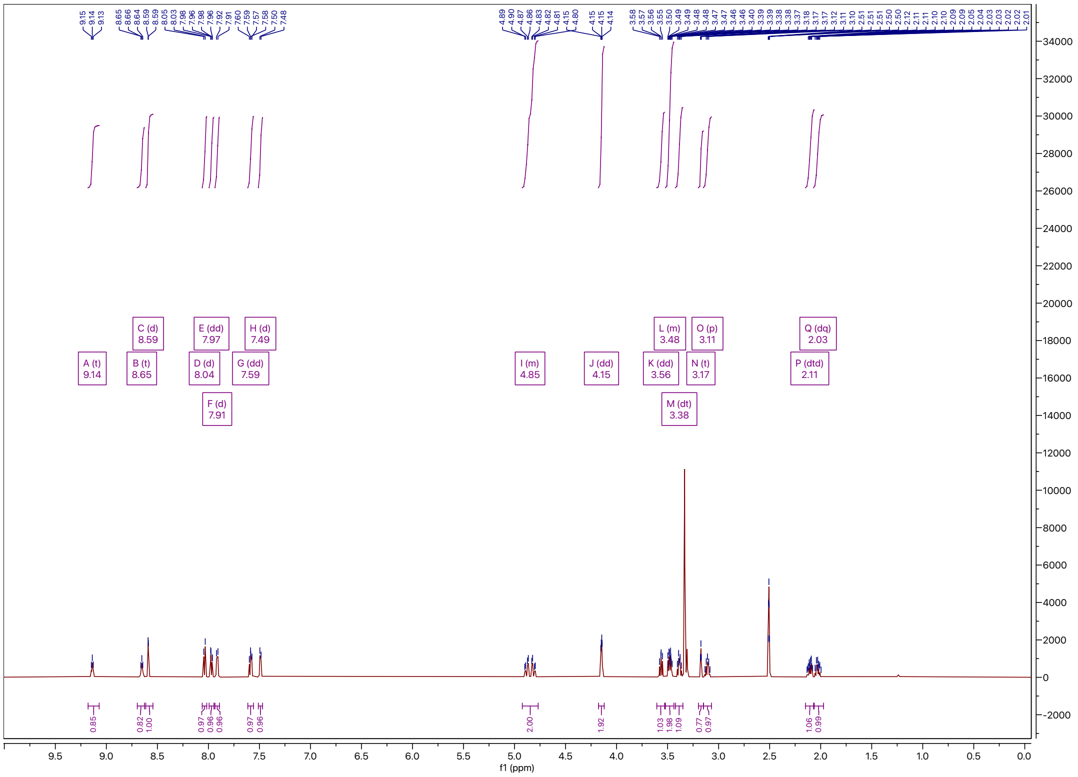

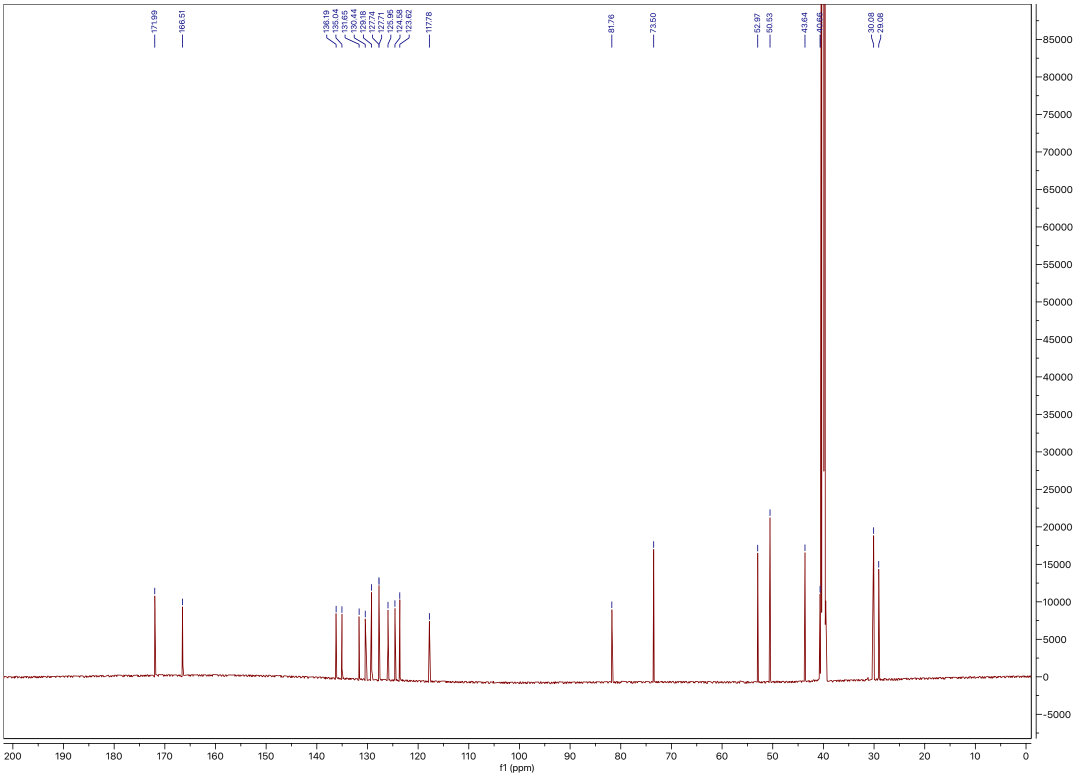

CNP6

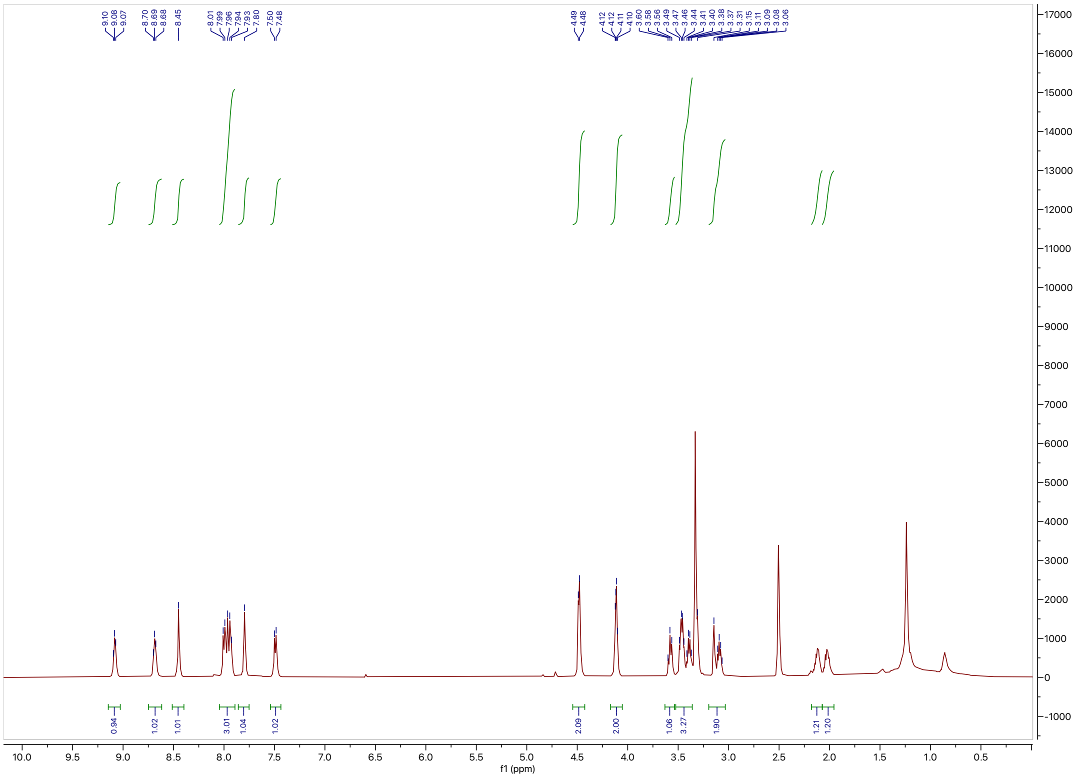

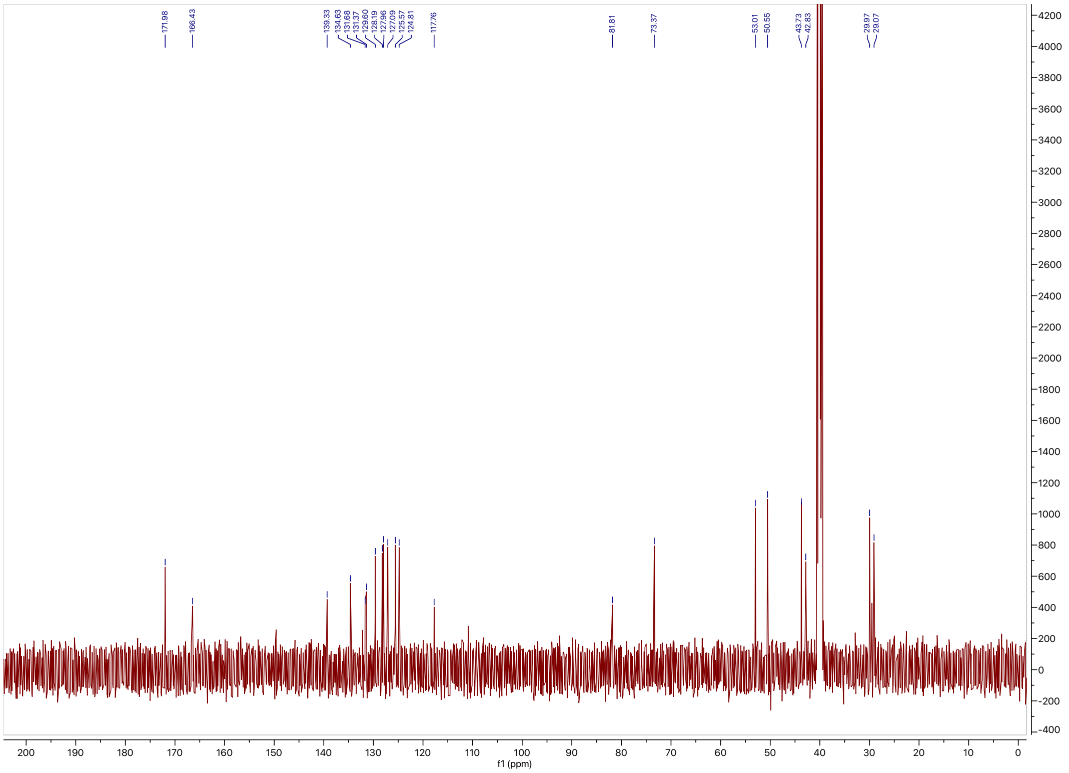

CNP7

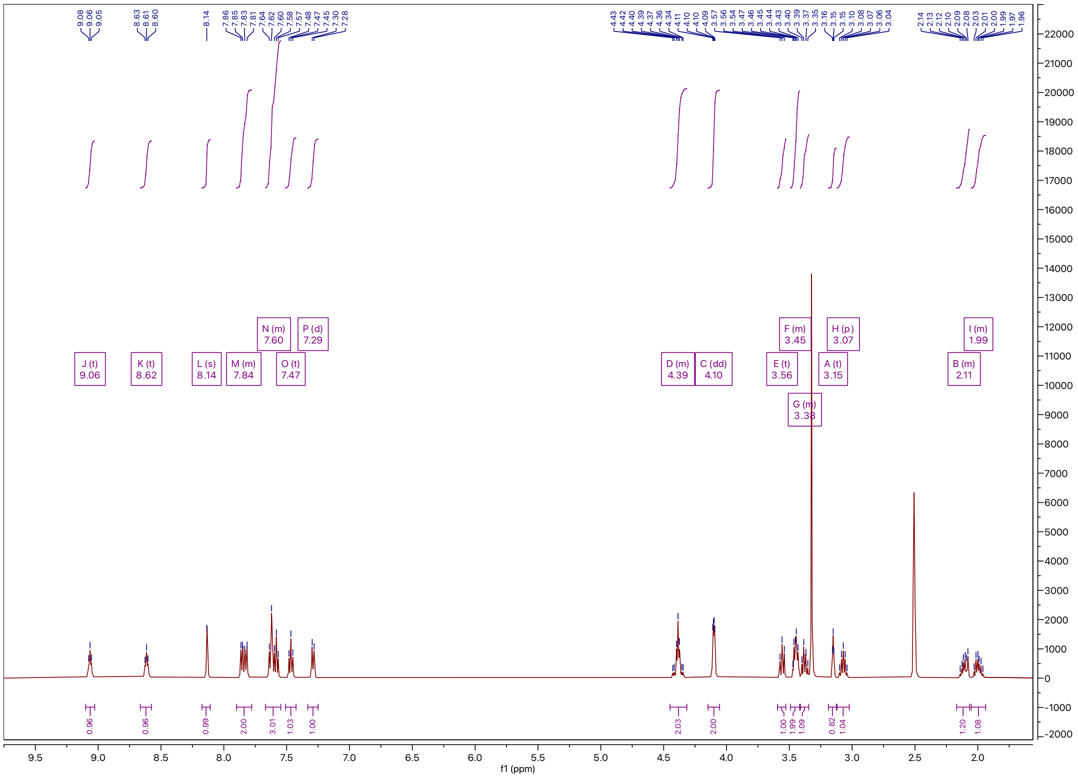

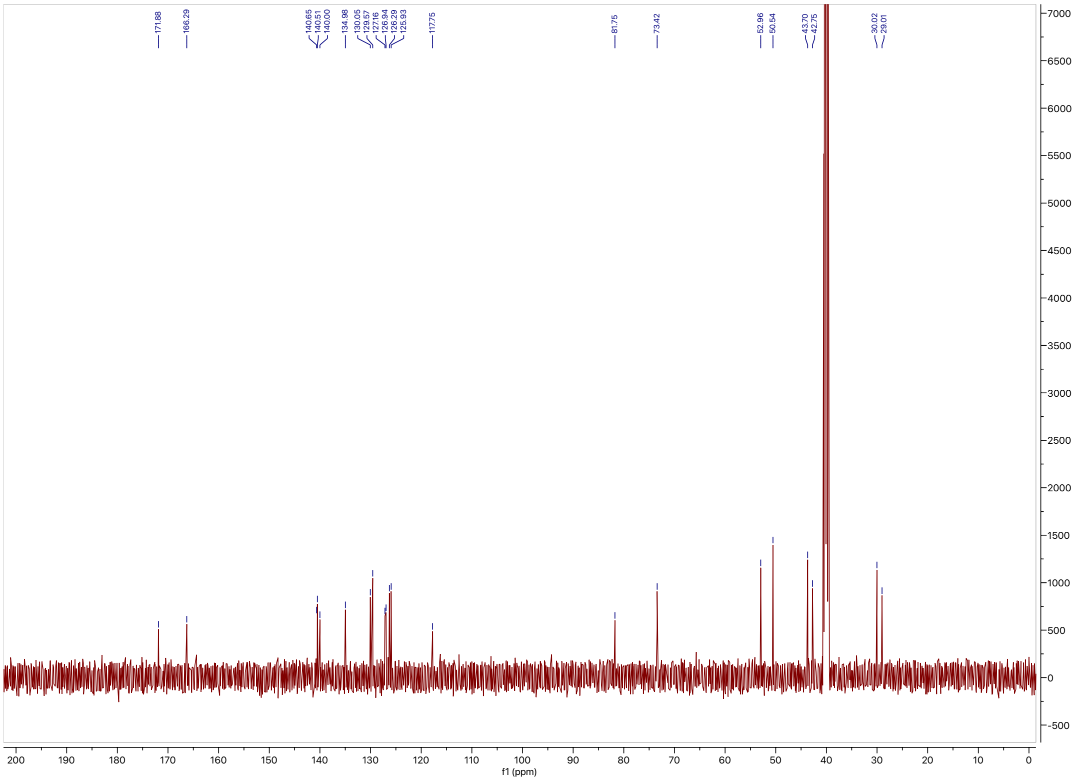

CNP8

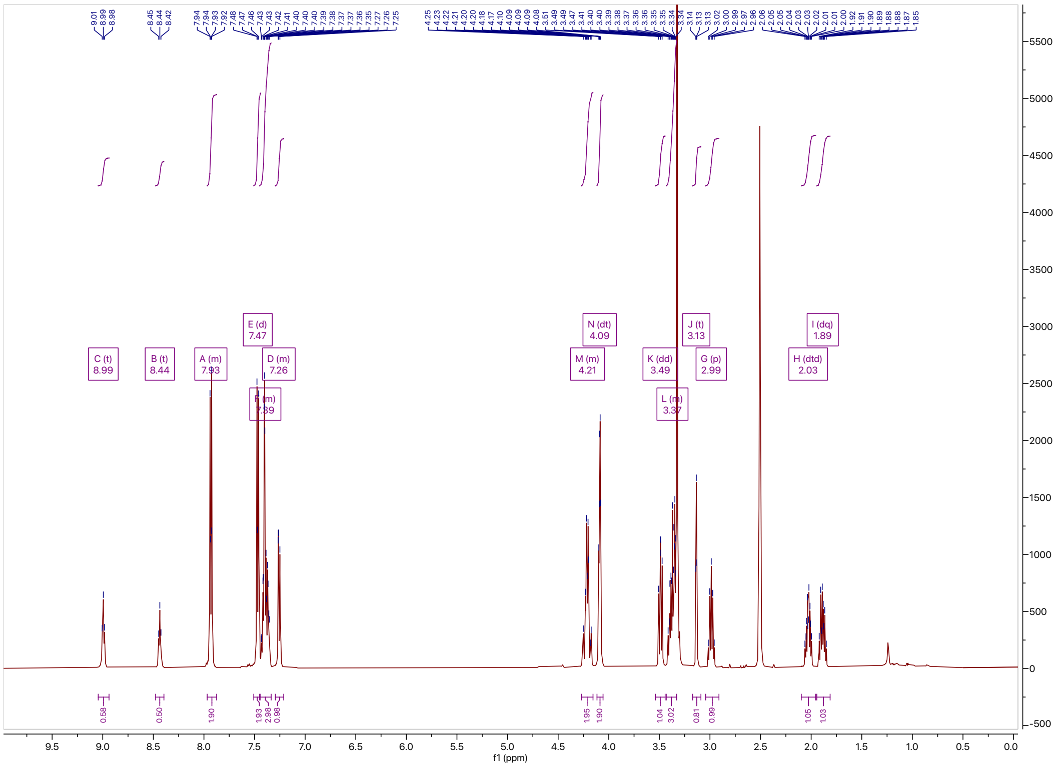

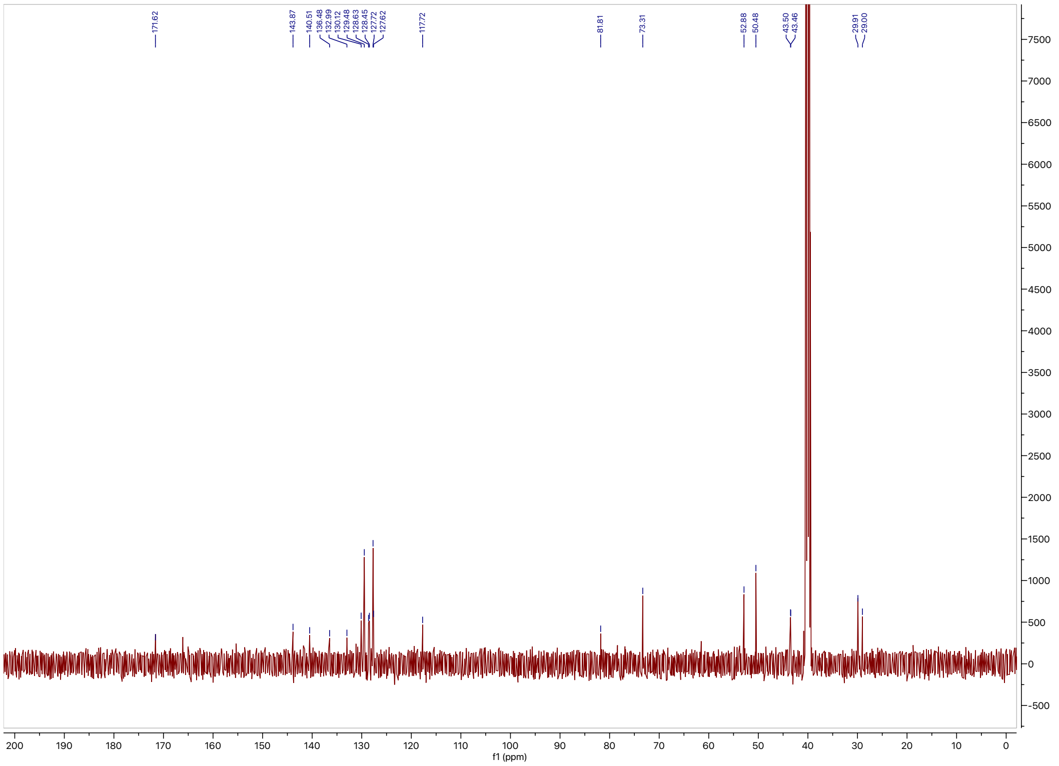

CNP9

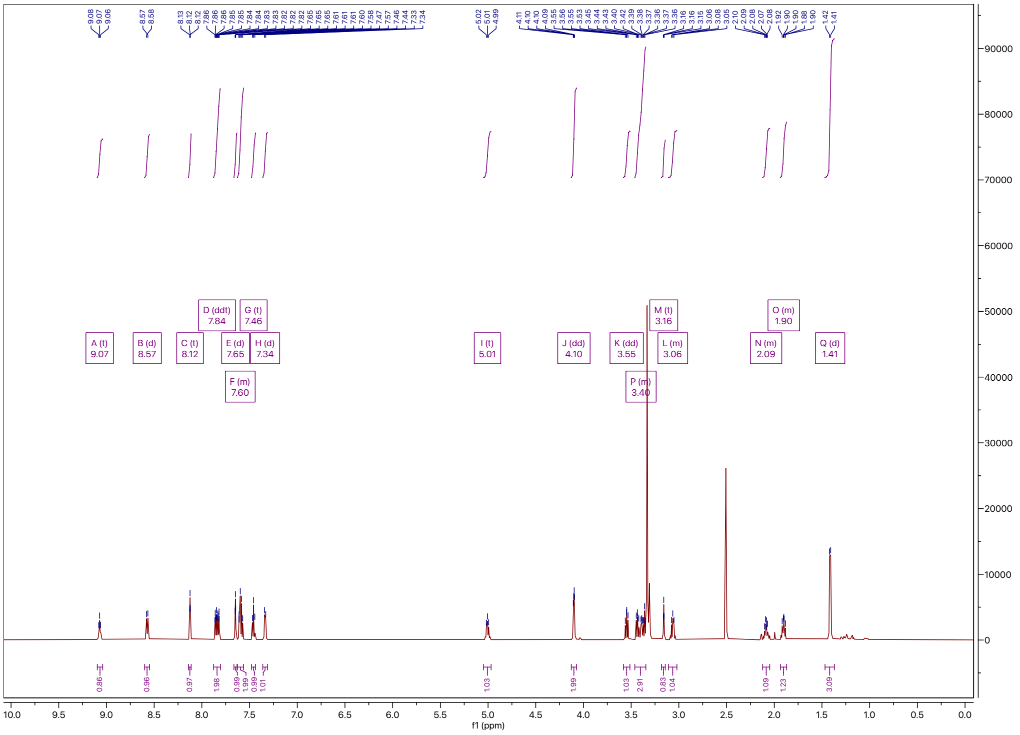

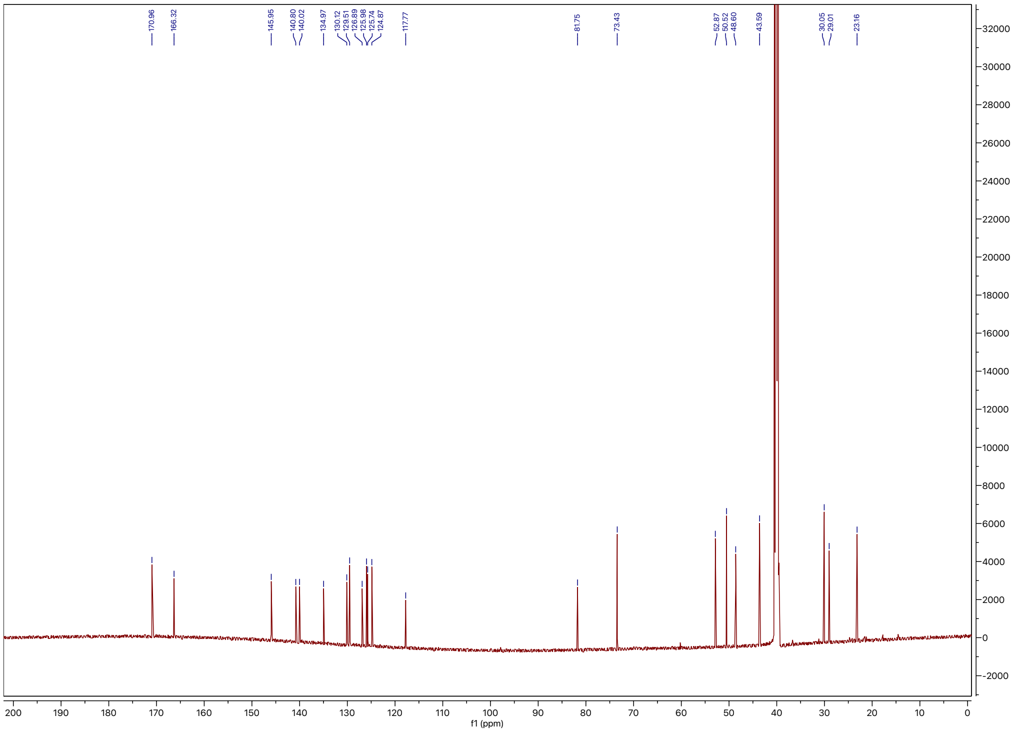

CNP10

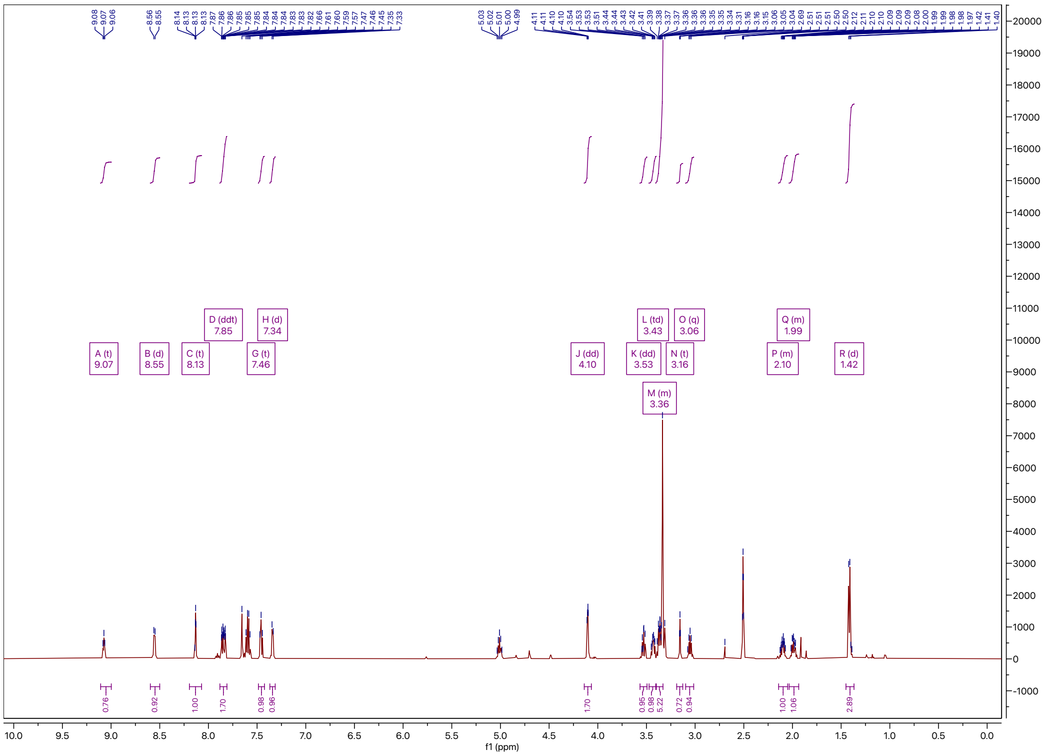

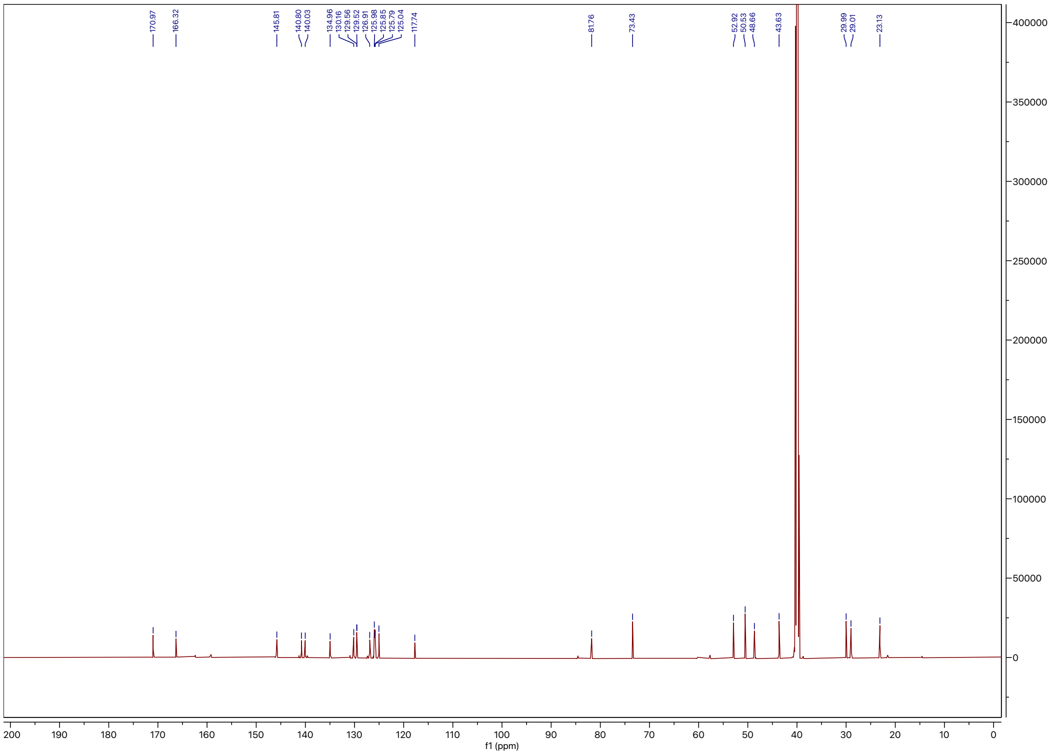

CNP11

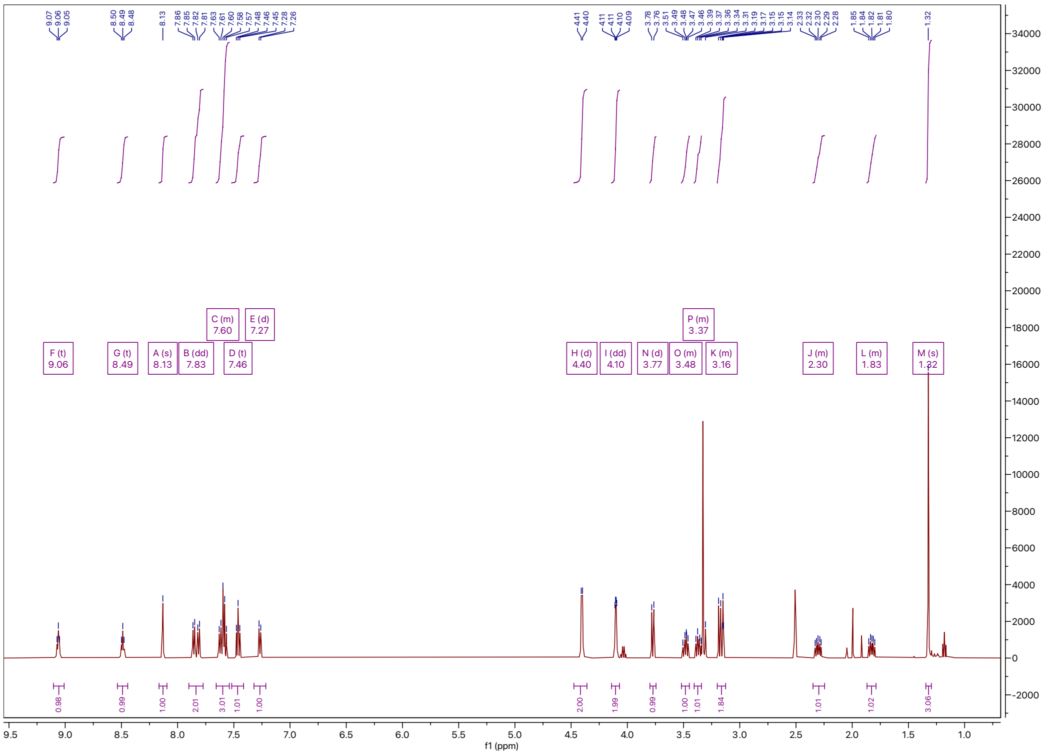
F (t)
9.06
G (t)
8.49
A (s)
8.13
B (dd)
7.83
C (m)
7.60
D (t)
7.46
E (d)
7.27
H (d)
4.40
I (dd)
4.10
N (d)
3.77
O (m)
3.48
P (m)
3.37
K (m)
3.16
J (m)
2.30
L (m)
1.83
M (s)
1.32
f1 (ppm)

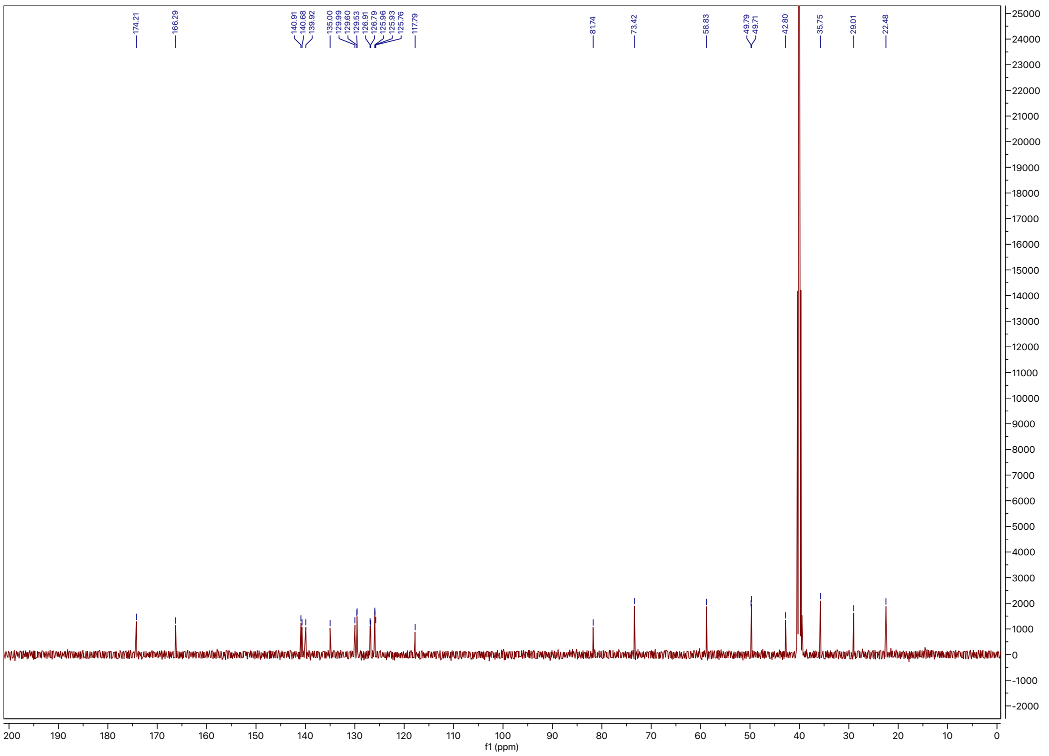
174.21
166.29
140.91
140.68
139.92
135.00
129.99
129.60
129.53
126.91
126.79
125.96
125.93
125.76
117.79
81.74
73.42
58.83
49.79
49.71
42.80
35.75
29.01
22.48
f1 (ppm)

CNP12

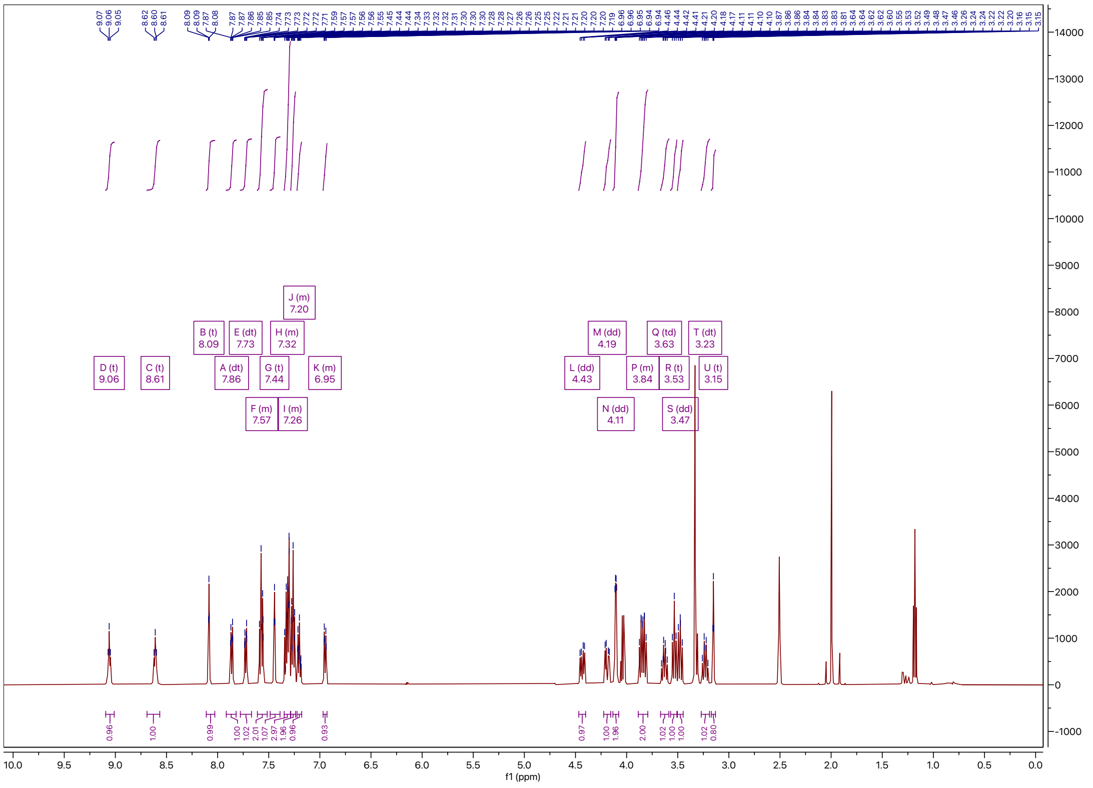

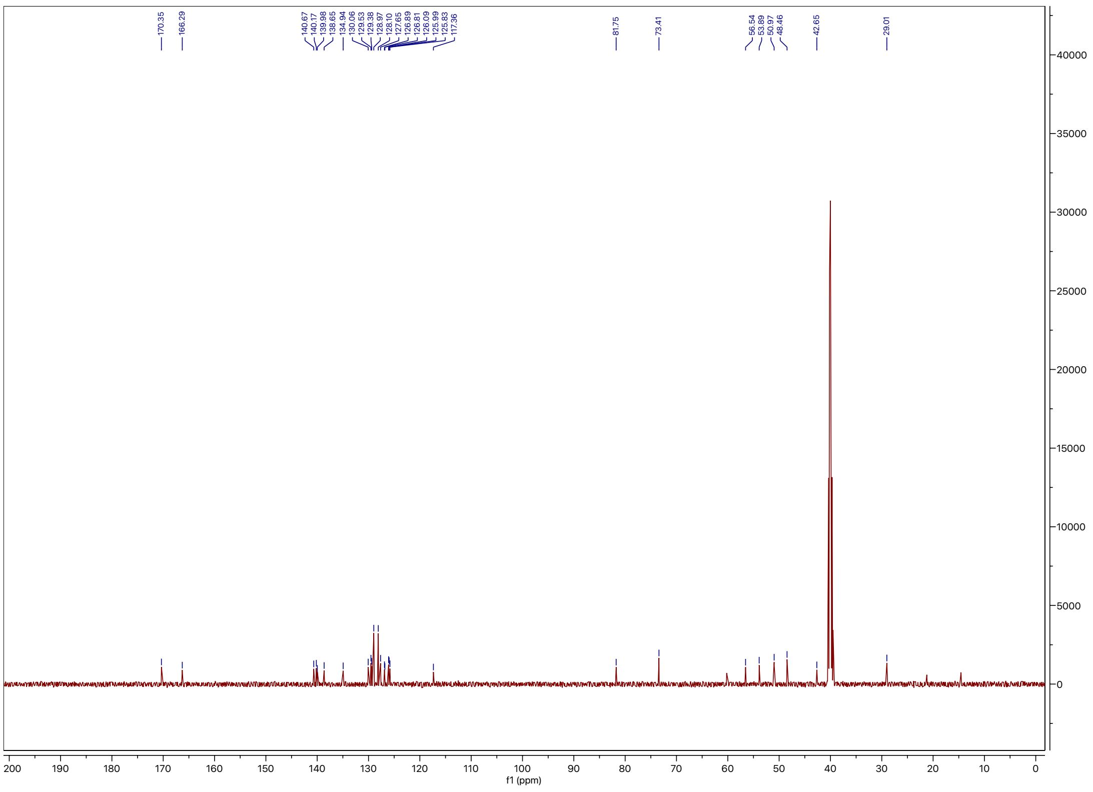

CNP13

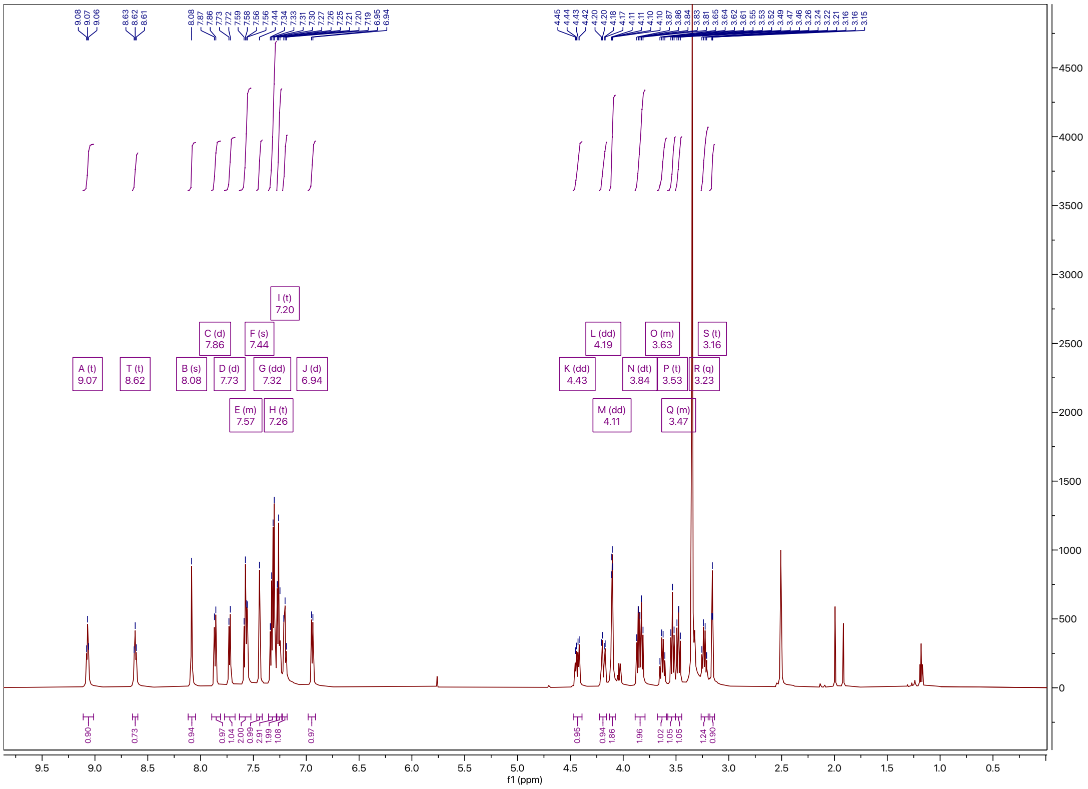

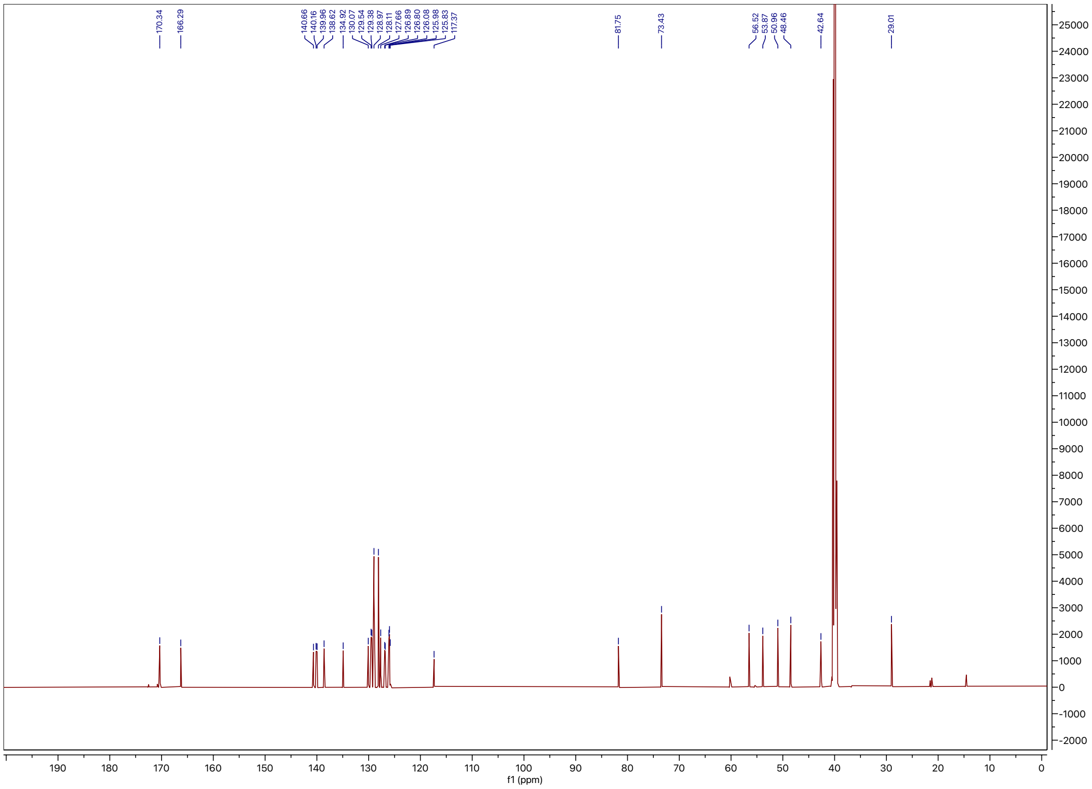

CNP7-Biotin

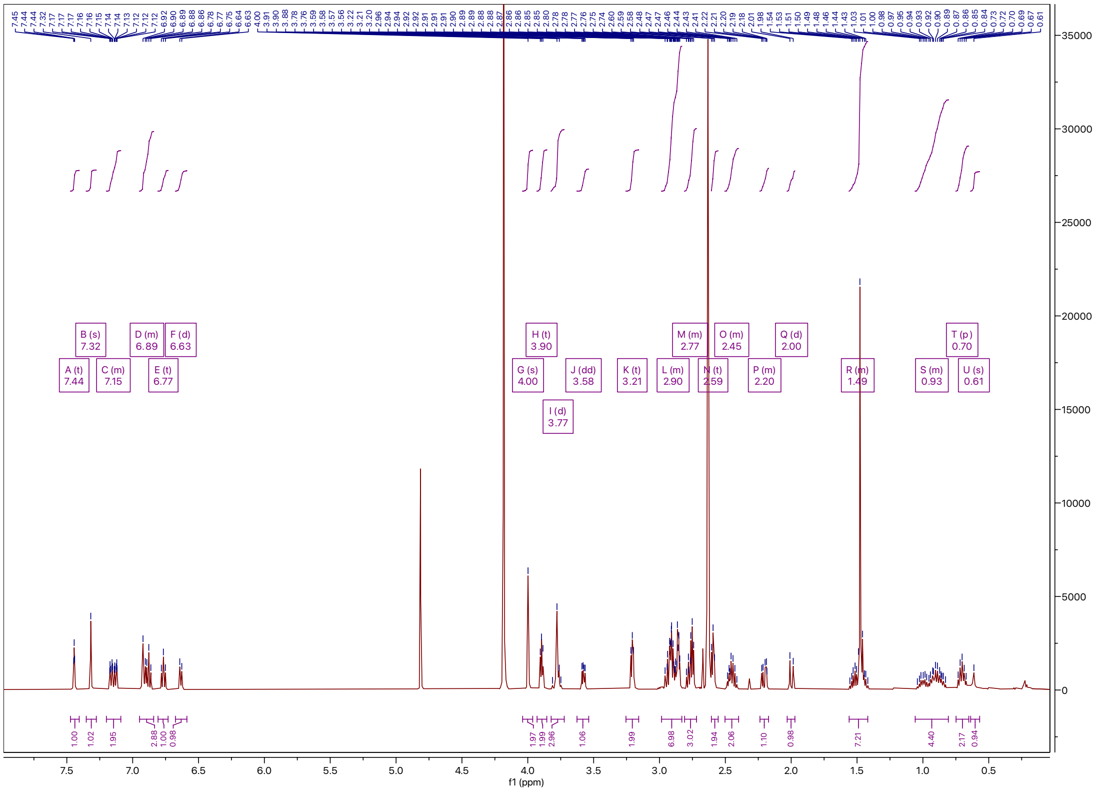

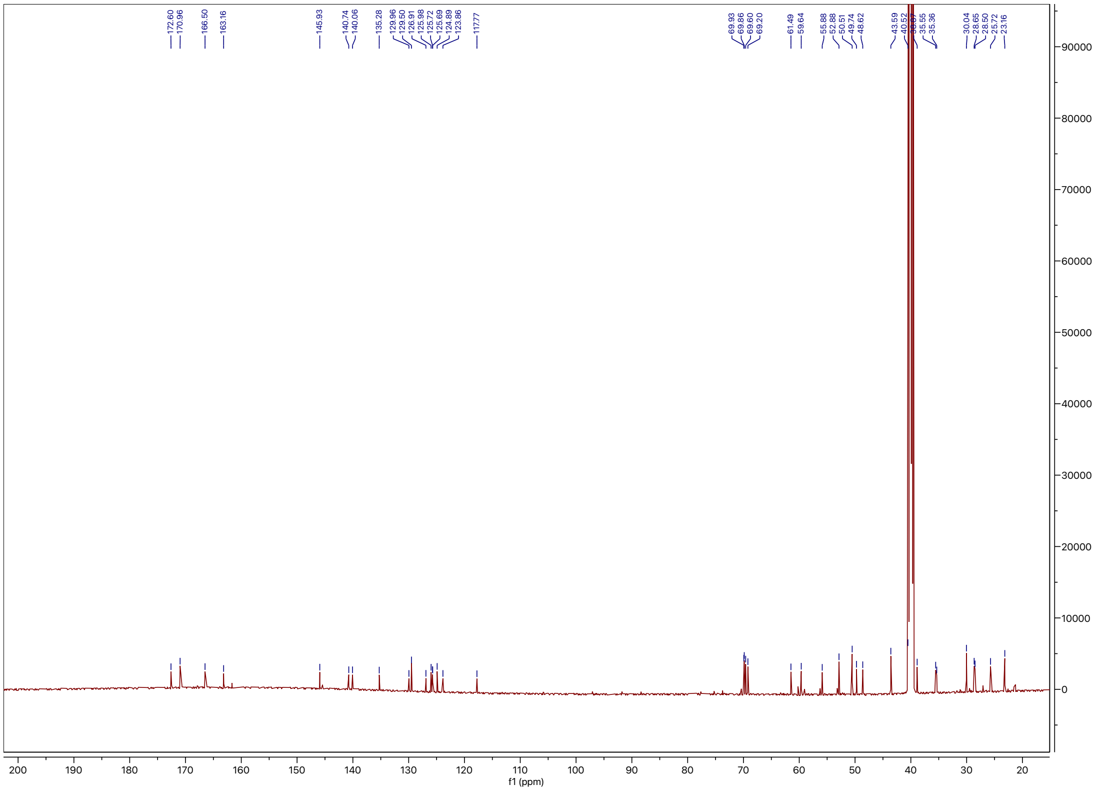

CNP9-Biotin

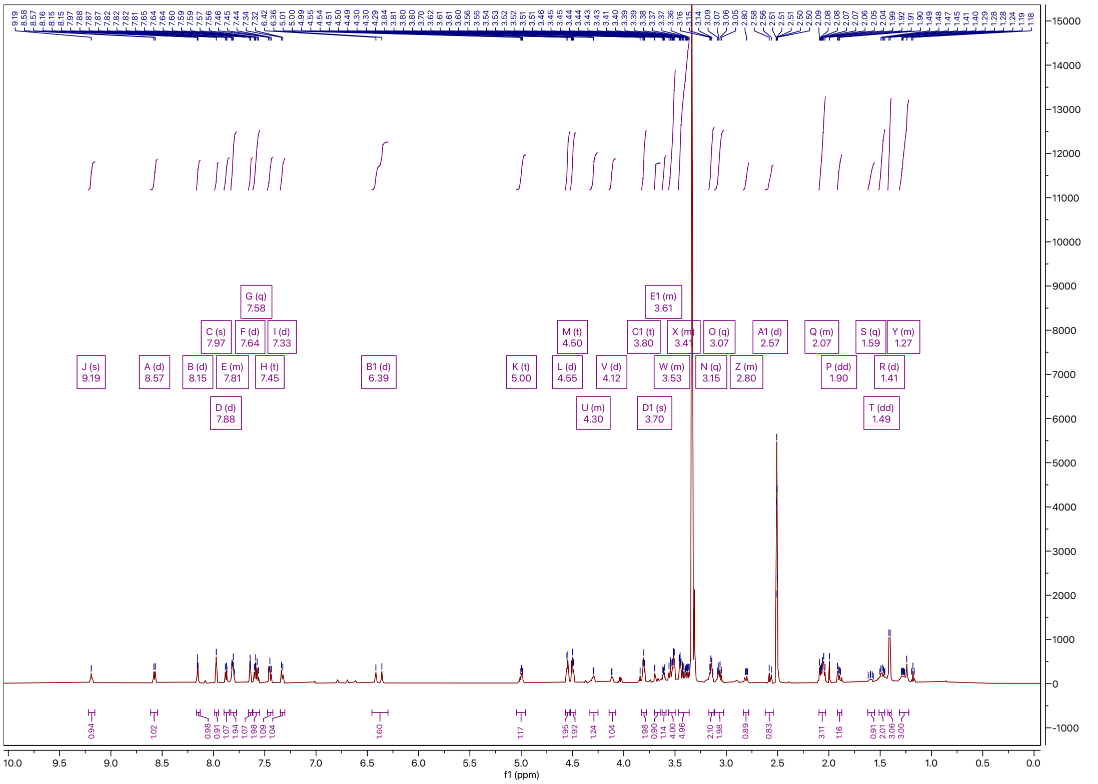

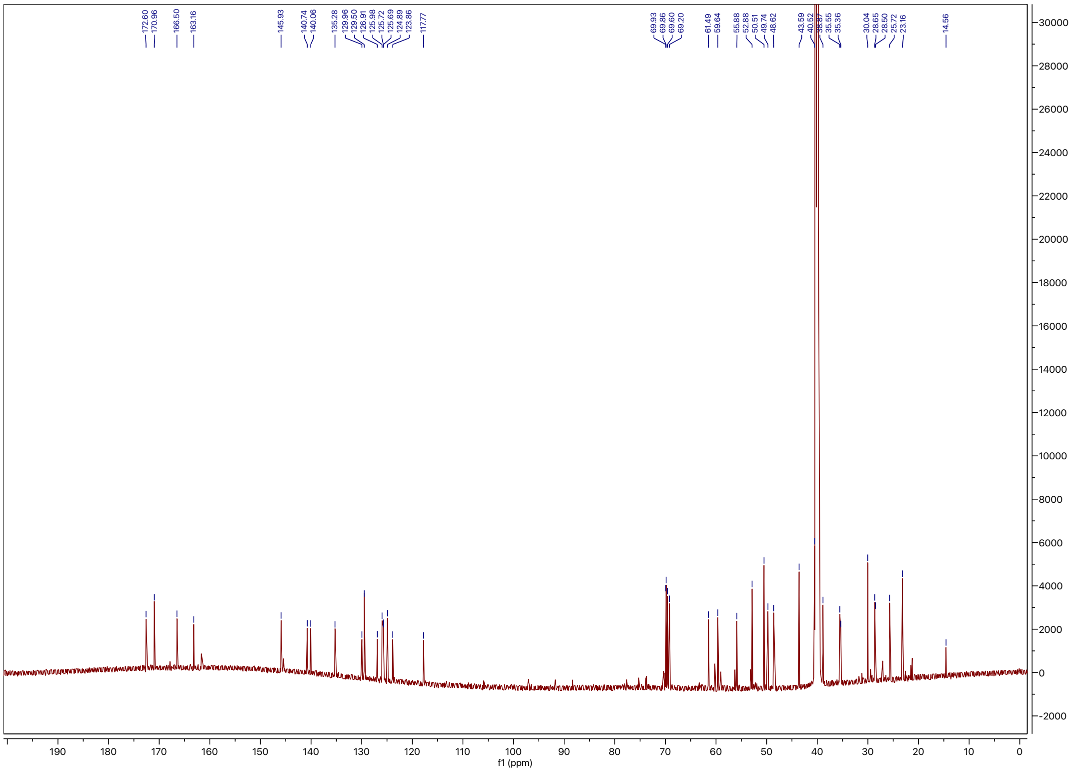

(1) Yi, S. A.; Sepic, S.; Schulman, B. A.; Ordureau, A.; An, H. mTORC1-CTLH E3 ligase regulates the degradation of HMG-CoA synthase 1 through the Pro/N-degron pathway. *Mol Cell* **2024**, *84* (11), 2166-2184 e2169. DOI: 10.1016/j.molcel.2024.04.026 From NLM Medline.
(2) Hughes, C. S.; Foehr, S.; Garfield, D. A.; Furlong, E. E.; Steinmetz, L. M.; Krijgsveld, J. Ultrasensitive proteome analysis using paramagnetic bead technology. *Mol Syst Biol* **2014**, *10* (10), 757. DOI: 10.15252/msb.20145625 From NLM Medline.
(3) Wang, Y.; Yang, F.; Gritsenko, M. A.; Wang, Y.; Clauss, T.; Liu, T.; Shen, Y.; Monroe, M. E.; Lopez-Ferrer, D.; Reno, T.; et al. Reversed-phase chromatography with multiple fraction concatenation strategy for proteome profiling of human MCF10A cells. *Proteomics* **2011**, *11* (10), 2019-2026. DOI: 10.1002/pmic.201000722 From NLM Medline.
(4) Gaetani, M.; Sabatier, P.; Saei, A. A.; Beusch, C. M.; Yang, Z.; Lundstrom, S. L.; Zubarev, R. A. Proteome Integral Solubility Alteration: A High-Throughput Proteomics Assay for Target Deconvolution. *J Proteome Res* **2019**, *18* (11), 4027-4037. DOI: 10.1021/acs.jproteome.9b00500 From NLM Medline.
(5) Locke, T. M.; Fields, R.; Gizinski, H.; Otto, G. M.; MacEwen, M. J. S.; Rusnac, D. V.; He, P.; Shechner, D. M.; McGann, C. D.; Berg, M. D.; et al. High-throughput identification of calcium-regulated proteins across diverse proteomes. *Cell Rep* **2024**, *43* (11), 114879. DOI: 10.1016/j.celrep.2024.114879 From NLM Medline.
(6) Van Vranken, J. G.; Li, J.; Mintseris, J.; Wei, T. Y.; Sniezek, C. M.; Gadzuk-Shea, M.; Gygi, S. P.; Schweppe, D. K. Large-scale characterization of drug mechanism of action using proteome-wide thermal shift assays. *Elife* **2024**, *13*. DOI: 10.7554/eLife.95595 From NLM Medline.
(7) McAlister, G. C.; Nusinow, D. P.; Jedrychowski, M. P.; Wuhr, M.; Huttlin, E. L.; Erickson, B. K.; Rad, R.; Haas, W.; Gygi, S. P. MultiNotch MS3 enables accurate, sensitive, and multiplexed detection of differential expression across cancer cell line proteomes. *Anal Chem* **2014**, *86* (14), 7150-7158. DOI: 10.1021/ac502040v From NLM Medline.
(8) Paulo, J. A.; O'Connell, J. D.; Gygi, S. P. A Triple Knockout (TKO) Proteomics Standard for Diagnosing Ion Interference in Isobaric Labeling Experiments. *J Am Soc Mass Spectrom* **2016**, *27* (10), 1620-1625. DOI: 10.1007/s13361-016-1434-9 From NLM Medline.
(9) Schweppe, D. K.; Prasad, S.; Belford, M. W.; Navarrete-Perea, J.; Bailey, D. J.; Huguet, R.; Jedrychowski, M. P.; Rad, R.; McAlister, G.; Abbatiello, S. E.; et al. Characterization and Optimization of Multiplexed Quantitative Analyses Using High-Field Asymmetric-Waveform Ion Mobility Mass Spectrometry. *Anal Chem* **2019**, *91* (6), 4010-4016. DOI: 10.1021/acs.analchem.8b05399 From NLM Medline.
(10) Erickson, B. K.; Mintseris, J.; Schweppe, D. K.; Navarrete-Perea, J.; Erickson, A. R.; Nusinow, D. P.; Paulo, J. A.; Gygi, S. P. Active Instrument Engagement Combined with a Real-Time Database Search for Improved Performance of Sample Multiplexing Workflows. *J Proteome Res* **2019**, *18* (3), 1299-1306. DOI: 10.1021/acs.jproteome.8b00899 From NLM Medline.
(11) Schweppe, D. K.; Eng, J. K.; Yu, Q.; Bailey, D.; Rad, R.; Navarrete-Perea, J.; Huttlin, E. L.; Erickson, B. K.; Paulo, J. A.; Gygi, S. P. Full-Featured, Real-Time Database Searching Platform Enables Fast and Accurate Multiplexed Quantitative Proteomics. *J Proteome Res* **2020**, *19* (5), 2026-2034. DOI: 10.1021/acs.jproteome.9b00860 From NLM Medline.
(12) Yang, K.; Whitehouse, R. L.; Dawson, S. L.; Zhang, L.; Martin, J. G.; Johnson, D. S.; Paulo, J. A.; Gygi, S. P.; Yu, Q. Accelerating multiplexed profiling of protein-ligand interactions: High-throughput plate-based reactive cysteine profiling with minimal input. *Cell*

Chem Biol **2024**, *31* (3), 565-576 e564. DOI: 10.1016/j.chembiol.2023.11.015 From NLM Medline.
(13) Eng, J. K.; Hoopmann, M. R.; Jahan, T. A.; Egertson, J. D.; Noble, W. S.; MacCoss, M. J. A deeper look into Comet--implementation and features. *J Am Soc Mass Spectrom* **2015**, *26* (11), 1865-1874. DOI: 10.1007/s13361-015-1179-x.
(14) Eng, J. K.; Jahan, T. A.; Hoopmann, M. R. Comet: an open-source MS/MS sequence database search tool. *Proteomics* **2013**, *13* (1), 22-24. DOI: 10.1002/pmic.201200439.
(15) Rad, R.; Li, J.; Mintseris, J.; O'Connell, J.; Gygi, S. P.; Schweppe, D. K. Improved Monoisotopic Mass Estimation for Deeper Proteome Coverage. *J Proteome Res* **2021**, *20* (1), 591-598. DOI: 10.1021/acs.jproteome.0c00563.
(16) Elias, J. E.; Gygi, S. P. Target-decoy search strategy for increased confidence in large-scale protein identifications by mass spectrometry. *Nat Methods* **2007**, *4* (3), 207-214. DOI: 10.1038/nmeth1019.
(17) Huttlin, E. L.; Jedrychowski, M. P.; Elias, J. E.; Goswami, T.; Rad, R.; Beausoleil, S. A.; Villen, J.; Haas, W.; Sowa, M. E.; Gygi, S. P. A tissue-specific atlas of mouse protein phosphorylation and expression. *Cell* **2010**, *143* (7), 1174-1189. DOI: 10.1016/j.cell.2010.12.001.
(18) Savitski, M. M.; Wilhelm, M.; Hahne, H.; Kuster, B.; Bantscheff, M. A Scalable Approach for Protein False Discovery Rate Estimation in Large Proteomic Data Sets. *Mol Cell Proteomics* **2015**, *14* (9), 2394-2404. DOI: 10.1074/mcp.M114.046995.
(19) Tyanova, S.; Temu, T.; Sinitcyn, P.; Carlson, A.; Hein, M. Y.; Geiger, T.; Mann, M.; Cox, J. The Perseus computational platform for comprehensive analysis of (prote)omics data. *Nat Methods* **2016**, *13* (9), 731-740. DOI: 10.1038/nmeth.3901 From NLM Medline.
(20) Punjani, A.; Rubinstein, J. L.; Fleet, D. J.; Brubaker, M. A. cryoSPARC: algorithms for rapid unsupervised cryo-EM structure determination. *Nat Methods* **2017**, *14* (3), 290-296. DOI: 10.1038/nmeth.4169 From NLM Medline.
(21) Scheres, S. H.; Chen, S. Prevention of overfitting in cryo-EM structure determination. *Nat Methods* **2012**, *9* (9), 853-854. DOI: 10.1038/nmeth.2115 From NLM Medline.
(22) Adams, P. D.; Afonine, P. V.; Bunkoczi, G.; Chen, V. B.; Davis, I. W.; Echols, N.; Headd, J. J.; Hung, L. W.; Kapral, G. J.; Grosse-Kunstleve, R. W.; et al. PHENIX: a comprehensive Python-based system for macromolecular structure solution. *Acta Crystallogr D Biol Crystallogr* **2010**, *66* (Pt 2), 213-221. DOI: 10.1107/S0907444909052925 From NLM Medline.
(23) Moriarty, N. W.; Grosse-Kunstleve, R. W.; Adams, P. D. electronic Ligand Builder and Optimization Workbench (eLBOW): a tool for ligand coordinate and restraint generation. *Acta Crystallogr D Biol Crystallogr* **2009**, *65* (Pt 10), 1074-1080. DOI: 10.1107/S0907444909029436 From NLM Medline.
(24) Pettersen, E. F.; Goddard, T. D.; Huang, C. C.; Couch, G. S.; Greenblatt, D. M.; Meng, E. C.; Ferrin, T. E. UCSF Chimera--a visualization system for exploratory research and analysis. *J Comput Chem* **2004**, *25* (13), 1605-1612. DOI: 10.1002/jcc.20084 From NLM Medline.
(25) Goddard, T. D.; Huang, C. C.; Meng, E. C.; Pettersen, E. F.; Couch, G. S.; Morris, J. H.; Ferrin, T. E. UCSF ChimeraX: Meeting modern challenges in visualization and analysis. *Protein Sci* **2018**, *27* (1), 14-25. DOI: 10.1002/pro.3235 From NLM Medline.
(26) Emsley, P.; Lohkamp, B.; Scott, W. G.; Cowtan, K. Features and development of Coot. *Acta Crystallogr D Biol Crystallogr* **2010**, *66* (Pt 4), 486-501. DOI: 10.1107/S0907444910007493 From NLM Medline.

(27) Yi, S. A.; Sun, L.; Rao, Y.; Ordureau, A.; Lewis, J. S.; An, H. Activity-based probes and chemical proteomics uncover the biological impact of targeting HMG-CoA Synthase 1 in the mevalonate pathway. *J Biol Chem* **2025**, *301* (10), 110660. DOI: 10.1016/j.jbc.2025.110660 From NLM Publisher.
