# Supplementary material for: Comprehensive Chemoproteomics Unveils Selective HMG-CoA Synthase 1 Inhibitors for Targeting Mevalonate Metabolism in Cancer
Source: J Am Chem Soc. 2026 May 13;148(20):20705–19. doi: 10.1021/jacs.6c02556 (PMC13220267; doi:10.1021/jacs.6c02556)
Supplement: Supplementary file 10 [file ja6c02556_si_010.pdf]

Cryo-EM data collection, refinement, and validation statistics

	HMGCS1~CNP7<br>(EMDB: EMD-73971)<br>(PDB 9ZAW)
**Data collection and processing**	
Nominal magnification (×)	165000
Voltage (kV)	300
Electron exposure (e^{-} Å^{-2})	60
Energy filter slit (eV)	10
Defocus range (μm)	-0.6 to -1.7
Exposure time (s)	2.93
Pixel size (Å)	0.725
Symmetry imposed	C2
Initial particle images (no.)	1900379
Final particle images (no.)	589925
Map resolution (Å)	2.29
Spherical Aberration (mm)	2.7
**Refinement**	
Initial model used (PDB code)	2P8U
Map sharpening *B* factor (Å^2)	83.6
Model composition	
Non-hydrogen atoms	7138
Protein residues	912
Ligands	2
B factors (Å^2)	
Protein	85.12
Ligand	49.94
R.m.s. deviations	
Bond lengths (Å)	0.004
Bond angles (°)	0.989
Validation	
MolProbity score	1.42
Clashscore	4.22
Poor rotamers (%)	1.33
Ramachandran plot	
Favored (%)	97.36
Allowed (%)	2.64
Disallowed (%)	0.00
